# Guidelines for mouse and human DC generation

**DOI:** 10.1002/eji.202249816

**Published:** 2022-10-27

**Authors:** Manfred B. Lutz, Shafaqat Ali, Cindy Audiger, Stella E. Autenrieth, Luciana Berod, Venetia Bigley, Laura Cyran, Marc Dalod, Jan Dörrie, Diana Dudziak, Georgina Flórez-Grau, Lucila Giusiano, Gloria J. Godoy, Marion Heuer, Anne B. Krug, Christian H. K. Lehmann, Christian T. Mayer, Shalin H. Naik, Stefanie Scheu, Gerty Schreibelt, Elodie Segura, Kristin Seré, Tim Sparwasser, Jurjen Tel, Huaming Xu, Martin Zenke

**Affiliations:** 1 Institute for Virology and Immunobiology, University of Würzburg, Würzburg, Germany; 2 Institute of Medical Microbiology and Hospital Hygiene, University of Düsseldorf, Düsseldorf, Germany; 3 Immunology Division, The Walter and Eliza Hall Institute of Medical Research, Parkville, VIC, 3052, Australia; 4 Department of Medical Biology, University of Melbourne, Parkville, VIC, 3052, Australia; 5 Dendritic Cells in Infection and Cancer (F171), German Cancer Research Center (DKFZ), Heidelberg, Germany; 6 Department of Internal Medicine II, University of Tübingen, Tübingen, Germany; 7 Institute of Molecular Medicine, University Medical Center of the Johannes Gutenberg-University Mainz, Mainz, 55131, Germany; 8 Translational and Clinical Research Institute, Newcastle University, Newcastle upon Tyne, NE2 4HH, United Kingdom; 9 CNRS, INSERM, Aix Marseille Univ, Centre d’Immunologie de Marseille-Luminy, Turing Center for Living Systems, Marseille, France; 10 RNA-based Immunotherapy, Hautklinik, Universitätsklinikum Erlangen (UKER), Friedrich-Alexander-Universität (FAU) Erlangen-Nürnberg, Erlangen, Germany; 11 Comprehensive Cancer Center Erlangen European Metropolitan Area of Nuremberg (CCC ER-EMN), Östliche Stadtmauerstraße 30, 91054, Erlangen, Germany; 12 Deutsches Zentrum Immuntherapie (DZI), Ulmenweg 18, 91054, Erlangen, Germany; 13 Laboratory of Dendritic Cell Biology, Department of Dermatology, University Hospital Erlangen, Hartmannstraße 14, D-91052, Erlangen, Germany; 14 Medical Immunology Campus Erlangen (MICE), D-91054, Erlangen, Germany; 15 Department of Tumor Immunology, Radboud Institute for Molecular Life Sciences, Radboudumc, Nijmegen, the Netherlands; 16 Institute of Medical Microbiology and Hygiene, University Medical Center of the Johannes Gutenberg-University Mainz, Mainz, 55131, Germany; 17 Institute for Immunology, Biomedical Center, Faculty of Medicine, Ludwig-Maximilians-University Munich, Planegg-Martinsried, Germany; 18 Experimental Immunology Branch, Center for Cancer Research, National Cancer Institute, National Institutes of Health, Bethesda, MD, 20892, USA; 19 Institut Curie, PSL Research University, INSERM, U932, 26 rue d’Ulm, Paris, 75005, France; 20 Institute for Biomedical Engineering, Department of Cell Biology, RWTH Aachen University Medical School, Aachen, Germany; 21 Helmholtz Institute for Biomedical Engineering, RWTH Aachen University, Aachen, Germany; 22 Laboratory of Immunoengineering, Department of Biomedical Engineering, Eindhoven University of Technology, Eindhoven, The Netherlands; 23 Institute for Complex Molecular Systems (ICMS), Eindhoven University of Technology, Eindhoven, The Netherlands

**Keywords:** Dendritic cells, Generation, Isolation, In vitro

## Abstract

This article is part of the Dendritic Cell Guidelines article series, which provides a collection of state-of-the-art protocols for the preparation, phenotype analysis by flow cytometry, generation, fluorescence microscopy, and functional characterization of mouse and human dendritic cells (DC) from lymphoid organs and various non-lymphoid tissues. This article provides protocols with top ticks and pitfalls for preparation and successful generation of mouse and human DC from different cellular sources, such as murine BM and HoxB8 cells, as well as human CD34^+^ cells from cord blood, BM, and peripheral blood or peripheral blood monocytes. We describe murine cDC1, cDC2, and pDC generation with Flt3L and the generation of BM-derived DC with GM-CSF. Protocols for human DC generation focus on CD34^+^ cell culture on OP9 cell layers for cDC1, cDC2, cDC3, and pDC subset generation and DC generation from peripheral blood monocytes (MoDC). Additional protocols include enrichment of murine DC subsets, CRISPR/Cas9 editing, and clinical grade human DC generation. While all protocols were written by experienced scientists who routinely use them in their work, this article was also peer-reviewed by leading experts and approved by all co-authors, making it an essential resource for basic and clinical DC immunologists.

## 1 Mouse DC

### 1.1 Preparation of source

#### 1.1.1 Isolation of murine BM cells by syringe or centrifugation

##### 1.1.1.1 Introduction.

We describe a step-by-step protocol for the isolation of mouse BM. We aim to supply researchers a consensus protocol, which is extensively used in our labs. The BM isolated with this method can be applied for direct analysis in flow cytometry, reconstitution of BM of lethally irradiated mice, purification of pluripotent progenitor and precursors, as well as for the development of different cell populations using different cell culture conditions. The preparation of sterile single cell suspensions from mouse BM includes an unsterile bone preparation step followed by a sterile step to flush the marrow cells.

##### 1.1.1.2 Materials and equipment.

- Ice-cold PBS
- Cell culture medium, RPMI 1640 without supplements
- RPMI 1640 medium supplemented with 10%FCS, 0.2mM 2-Mercaptoethanol, 2mM l-Glutamin).
- Isopropanol 70% or Ethanol 70% for bone disinfection
- Erythrocyte lysis buffer (ACK lysing buffer)
- Sterile 20 ml-syringe for flushing
- Cannulas, 24–27G for flushing
- Sterile 10 ml-syringe with 20-gauge needle for making a hole in the Eppendorf tubes
- Surgical forceps (straight)
- Surgical scissors
- Pipettes
- Pipette boy
- Pipette tips
- Filter mesh
- 50 ml tubes
- Eppendorf tubes 1.5 ml and 0.5 ml (make hole in the bottom of the 0.5 ml tube using a 20G needle)
- Laminar flow hood for cell culture with vertical laminar flow
- Centrifuge (no temperature control is needed)
- Phase-contrast microscope
- Neubauer Chamber/Hemocytometer to count cells

##### 1.1.1.3 Step-by-step sample preparation.

1. carefully remove femur and tibia of both hind limbs without breaking the bones.
2. prepare three petri-dishes:
  - Containing 10–20 ml ice cold sterile PBS for bone preparation
  - Containing isopropanol 70% or ethanol 70% for disinfection
  - Containing ice cold sterile PBS for rinsing bones after disinfection
3. remove muscles and tissue by using cleansing tissue (unsterile) and put the clean bones in PBS. After this step all work is conducted in a Laminar flow hood using proper sterile conditions.
4. disinfect intact bones by incubation in 70% isopropanol or 70% ethanol for 3–4 min.
5. transfer the sterilized bones in the sterile ice-cold PBS until further use.

For harvesting BM by flushing using a syringe, continue with step 6. Alternatively, for harvesting BM by centrifugation continue directly with step 12.

###### Harvesting BM by flushing

- from now on sterile work with sterile tools: cut off ends of femur/tibia and flush out BM with PBS or RPMI 1640 medium without FCS in syringe in a fresh Petri dish from both cut ends until the bones appear white.
- dissect the clumps by sucking and streaming out the suspension several times through a needle attached to the syringe (from previous step). Pipetting up and down with a serological pipette will give the same result. Pipet up and down about 10 times to disintegrate the biggest clumps.
- collect cells and transfer all cells afterward in a 50 ml falcon tube.
- centrifuge (5 min, 300–500 × *g* = 1200–1500 rpm), at room temperature or 4°C.
- if erythrocyte lysis is required, proceed to step 17.
- resuspend cell pellet in 10 ml RPMI medium or PBS containing FCS.

###### Harvesting BM by centrifugation

- from now on sterile work with sterile tools: make a hole at the bottom of a sterile 0.5 ml Eppendorf tube using a sterile 20G needle attached to a syringe and place it in the 1.5 ml Eppendorf tube.
- hold the bones one by one with sterile forceps, cut the knee joint ends of the tibia and femur just below the end of the marrow cavity using a sterile sharp scissor.
- place the bones with cut ends downside in the 0.5 ml Eppendorf tube with a hole that was pre-placed in a 1.5ml Eppendorf tube.
- carefully close the cap of the 0.5 ml Eppendorf tube and put the whole assembly in the centrifuge.
- centrifuge the bones for 15 s at 10,000 × *g*. The BM will be collected at the bottom of the 1.5 ml Eppendorf tube. The bones should be white at this stage.

Discard the 0.5 ml tubes with empty bones and proceed with the BM in the 1.5 ml Eppendorf tube. The BM cells can be resuspended in PBS or medium containing FCS for any application if erythrocytes lysis is not required. If erythrocyte lysis is needed proceed with the following steps.

###### Erythrocyte lysis

- resuspend well the BM in 3 ml of the erythrocyte lysis buffer, transfer the cell suspension into the 50 ml tube, and incubate at room temperature for 3 min.
- stop the erythrocyte lysis by adding 10 ml of medium or PBS.
- centrifuge (5 min, 300–500 × *g*, RT or 4°C) and resuspend the cell pellet in 10 ml of FCS containing medium.
- to remove any bone, muscle and cell clumps filter the cell suspension through a 70 mm filter mesh.
- determine the yield and viability of cells by Trypan blue exclusion and counting using a hemocytometer.

##### 1.1.1.4 Expected yield.

From a 6–12-wk-old C57BL/6 mouse the following number of the BM cells are expected:

- harvesting BM by flushing method: 25–40 million
- BM by centrifugation method: 50–70 million

##### 1.1.1.5 Pitfalls.

When dissecting the mice, make sure that legs do not get in contact with the fur (e.g., soak the fur with 70% ethanol before opening the skin, then pull the skin down and off the legs, avoiding to get hairs onto the legs). This is important to avoid contamination.

Make sure that during the excision of the leg or during the removal of the musculature, the bone remains intact. In case of the cut or broken bones, the alcohol disinfection will enter the inner part of the bone and kill the marrow cells. If the bone breaks avoid using ethanol, use only buffer for washing the bone.

##### 1.1.1.6 Top tricks.

Related to step 5: the bones can be kept in ice-cold PBS or medium if longer transport is required before proceeding.

Related to step 12: One may make holes in the 0.5 ml Eppendorf tubes for many experiments at once and sterilize them by autoclaving before the start of experiment.

Related to step 13–14: Up to four bones may fit in one 0.5 ml Eppendorf tube. Add one tibia followed by a femur and repeat it again to fit all four bones in one 0.5 ml Eppendorf tube.

Related to 15: The cap of the holder 1.5 ml Eppendorf tube will need some space. Therefore, do not use the full place capacity of the centrifuge. Always leave one place empty between the tubes. Alternatively, one can cut off the caps of the 1.5 ml Eppendorf tubes.

Related to step 16: If there is no BM at the bottom of the 1.5 ml tube then confirm the presence of the hole in the bottom of 0.5 ml tube. If there is, still some BM left in the bones then repeat the centrifugation step.

Related to step 21: In order to increase the yield one can also collect the BM from the hipbones (iliac bone).

#### 1.1.2 HoxB8 cell generation from mouse BM cells and their differentiation into cDC1, cDC2, and pDC subsets

##### 1.1.2.1 Introduction.

Current developments in gene editing, such as the CRISPR/Cas9 technology, provide particularly appealing opportunities for targeted deletion of DNA sequences to study gene regulation and gene function. However, often the limited life span of somatic cells represents a major roadblock for CRISPR/Cas9 application. For example, the precise deletion of cis-acting elements in promoter and enhancer sequences requires clonal cell populations, which are hardly obtained from primary mouse cells due to their limited life span. In this context, the conditional immortalization of BM stem/progenitor cells with HoxB8 provides an attractive solution [1, 2]. Such conditionally immortalized HoxB8 cells exhibit an extended lifespan and robust clonality, and can differentiate into a large array of cell types *in vitro* and *in vivo.*

**Figure 1.**
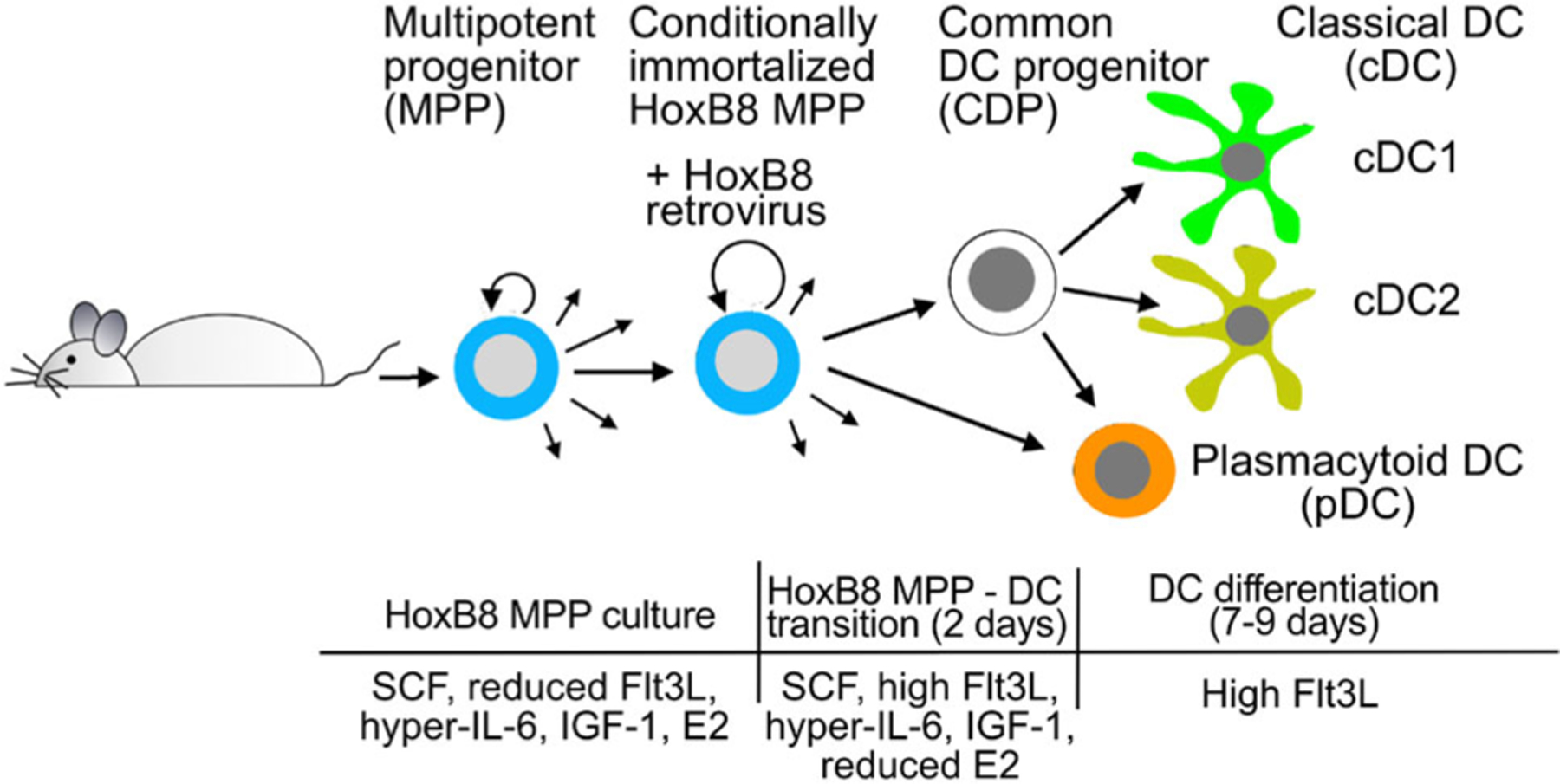
Schematic representation of HoxB8 cell generation and their differentiation into DC. Multipotent progenitors (MPP) from mouse BM are infected with HoxB8 retrovirus to yield conditionally immortalized HoxB8 MPP. HoxB8 MPP are grown with SCF, reduced Flt3L, IL-6/soluble IL-6 receptor fusion protein (hyper-IL-6), IGF-1, and β-estradiol (E2). HoxB8 MPP are induced to differentiate into DC via a HoxB8 MPP – DC transition phase with high Flt3L and reduced E2, and subsequent high Flt3L culture to obtain the cDC1, cDC2, and pDC subsets. Modified after [5].

Here we extend previous work on HoxB8 cells [1–5] and describe a protocol for mouse BM HoxB8 cells, referred to as HoxB8 multipotent progenitors (HoxB8 MPP). HoxB8 MPP can be expanded to large cell numbers, and clonal cell populations are readily obtained by limiting dilution. In addition, HoxB8 MPP yield all three major DC subsets (cDC1, cDC2, and pDC) and faithfully recapitulate the sequel MPP-CDP-cDC1/cDC2. HoxB8 MPP are obtained from BM by retroviral transduction with the β-estradiol (E2) inducible HoxB8 estrogen receptor fusion HoxB8-ER and cells are grown with a four-cytokine cocktail of SCF, Flt3 ligand (Flt3L), IGF-1, and IL-6/soluble IL-6 receptor fusion protein (hyper-IL-6) (Fig. 1) [6, 7]. Upon E2 withdrawal and addition of DC directing cytokines, HoxB8 MPP differentiate into DC.

##### 1.1.2.2 Materials.

###### 1.1.2.2.1 Reagents.

A complete list of reagents is provided in Table 1.

###### 1.1.2.2.2 Equipment.

Necessary equipment is listed in Table 2.

##### 1.1.2.3 Step-by-step sample preparation.

###### 1.1.2.3.1 Preparation of stocks, solutions and culture media.

####### Solutions and buffers for calcium phosphate precipitation

- Prepare 2 M CaCl_2_ solution in water, sterile filter, and store at +4°C.
- Prepare 2× Hepes-buffer saline (2×HBS)
  280 mM NaCl
  50 mM HEPES
  1.5 mM Na_2_HPO_4_
  Adjust to pH 7.12, store at +4°C.

####### Solutions for retrovirus concentration

- Prepare Chondroitin sulfate C (CSC) sodium salt, 80 mg/ml in water, sterile filter through 45 μm membrane filter (Whatman), store at +4°C.
- Prepare Polybrene (PB), 80 mg/ml solution in water, sterile filter through 45 μm membrane filter (Whatman), store at −20°C.
- Dissolve β-estradiol (E2) in ethanol at 10^−6^ M and store at −20°C.

####### Flow cytometry staining buffer

- Prepare cell staining buffer for flow cytometry analysis:
  246.25 ml PBS
  1.25 ml FBS (Gibco 10270106)
  2.5 ml 500 mM EDTA

####### Culture media for HEK293T cells and HoxB8 MPP

- HEK293T were from ATCC (https://www.atcc.org)
- Prepare HEK293T culture medium:
  450 ml DMEM
  50 ml FBS (PAA A01125–499)
  2 mM l-glutamine
  100 U/ml penicillin/streptomycin
- Prepare basic HoxB8 culture medium:
  450 ml RPMI 1640
  50 ml FBS (Gibco 10270106)
  2 mM l-glutamine
  100 U/ml penicillin/streptomycin
- Prepare fresh HoxB8 growth medium (see Fig. 1) for immediate use:
  Basic HoxB8 culture medium
  10^−6^ M E2
  Growth factors (see references [5] and [6]):
  - Recombinant murine SCF (Peprotech), 100 ng/ml (1:1000 from stock solution) or 1:100 from supernatant of CHO KLS C6 cells*
  - Recombinant human Flt3L (Peprotech), 25 ng/ml (1:1000 from stock solution) or 1:100 from supernatant of Flt3L producing B16F1 cells**
  - Recombinant murine IL-6/IL-6R alpha protein chimera (R&D Systems), 25 ng/ml (1:1000 from stock solution), or IL-6/soluble IL-6 receptor fusion protein (hyper-IL-6) (1:1000 from stock; kind gift from Dr. Stefan Rose-John, University of Kiel, Germany [8]
  - Human IGF-1 long range 40 ng/ml (1: 1000 from stock solution; Sigma)
- Prepare fresh DC differentiation transition phase medium (see Fig. 1) for immediate use:
  Basic HoxB8 culture medium
  10^−8^ M E2 (1:100 dilution from HoxB8 growth medium)
  Growth factors:
  - Recombinant murine SCF (Peprotech), 100 ng/ml (1:1000 from stock solution) or 1:100 from supernatant of CHO KLS C6 cells*
  - Recombinant human Flt3L (Peprotech), 25 ng/ml (1:1000 from stock solution) or 1:100 from supernatant of Flt3L producing B16F1 cells**
  - Recombinant murine IL-6/IL-6R alpha protein chimera (R&D Systems), 25 ng/ml (1:1000 from stock solution), or IL-6/soluble IL-6 receptor fusion protein (hyper-IL-6) (1:1000 from stock; kind gift from Dr. Stefan Rose-John, University of Kiel, Germany [8]
  - Human IGF-1 long range 40 ng/ml (1: 1000 from stock solution; Sigma)
  - Flt3L 50 ng/ml (1:500 from stock solution; Peprotech)
- Prepare fresh DC differentiation medium (see Fig. 1) for immediate use
  Basic HoxB8 culture medium
  Growth factors:
  - Flt3L 50 ng/ml (1:500 from stock solution; Peprotech)

* Note: Murine SCF is produced by the stably transfected CHO KLS C6 cell line expressing soluble murine SCF (kit ligand soluble; Genetics Institute, Cambridge, USA).

**Note: Murine Flt3L is produced by the stably transfected B16F1 cells melanoma cell line expressing murine Flt3L [9]. CHO-Flt3L-FLAG (Tracy Willson, The Walter and Eliza Hall Institute, WEHI, Melbourne, Victoria, Australia) can also be used.

Supernatants of cytokine producing cell lines need to be titrated to achieve the desired concentration for optimal use.

###### 1.1.2.3.2 Generation of HoxB8 MPP from mouse BM cells.

####### 1.1.2.3.2.1 Isolation and culture of mouse BM cells

See section 1.1.1

####### 1.1.2.3.2.2 Preparation of MSCV-ERHBD-HoxB8 retroviral particles

The MSCV-ERHBD-HoxB8 retroviral vector encoding the estrogen-inducible ERHBD-HoxB8 [1], the gag-pol plasmid pVPack_GP, and envelope plasmid pVPack_Eco (Agilent Technologies) are transfected into HEK293T cells by calcium phosphate co-precipitation for generation of MSCV-ERHBD-Hoxb8 retroviral particles (in the following referred to as HoxB8 retroviral particles). Retroviral particles are then concentrated with polybrene/chondroitin sulfate C (PB/CSC) precipitation [10] and used for infection of BM cells. The detailed procedure is as follows:

1.1.2.3.2.2.1 Transfection of HEK293T cell by calcium phosphate precipitation

1. Seed 0.75×10^6^ HEK293T cells in 6 cm tissue culture dish with 5 ml medium/dish about 16–24 h prior to transfection.
2. Refresh medium 1 h before transfection*.
3. Prepare DNA mix:
  5 μg MSCV-ERHBD-HoxB8 retroviral vector
  2.5 μg pVPack_GP gag-pol plasmid
  2.5 μg pVPack_Eco envelop plasmid
  31.25 μl 2 M CaCl_2_
4. Adjust volume with sterile water to 250 μl.
5. Add the 250 μl DNA-mix into 250 μl 2×HBS in a 15 ml Falcon tube while vortexing.
6. Incubate at room temperature for 15 min.
7. Pipette the calcium phosphate DNA precipitate dropwise to HEK293T cells while gently shaking the culture dish.
8. Check the quality of the calcium phosphate DNA precipitate on HEK293T cells by microscopy. The precipitate should be fine grained and stick to cells.
9. Refresh medium (5 ml) 16–24 h after transfection.

*Note: Confluency of HEK293T cells should be 70–80% before transfection.

1.1.2.3.2.2.2 Concentration of HoxB8 retrovirus by PB/CSC precipitation and BM cell infection

1. Harvest HoxB8 retrovirus from transfected HEK293T cells and concentrate retrovirus by PB/CSC precipitation [10] for BM cell infection.
2. Collect virus supernatant from transfected HEK293T cells on days 2 and 3 after transfection by pipetting and transfer into 15 ml Falcon tube. Add 5 ml fresh medium to HEK293T cells for the second harvest of virus supernatant.
3. Remove residual HEK293T cells from virus supernatant by centrifugation (1400 rpm, 5 min; perform this centrifugation step twice)*.
4. Add 5 μl PB and 5 μl CSC to 5 ml retrovirus supernatant (80 μg/ml PB and 80 μg/ml CSC final concentration, respectively)
5. Mix well by vortexing and incubate for 20 min at 37°C in a water bath.
6. Centrifuge (4,000 rpm, 20 min) to spin down retrovirus (check pellet by visual inspection).
7. Discard supernatant.
8. Resuspend the pellet with 50–100 μl basic HoxB8 culture medium by vigorously pipetting up and down at least 50 times.
9. Infect BM cells twice (day 2 and 3 after HEK293T cell transfection) with concentrated HoxB8 retrovirus**.
10. One to 2 days after retroviral infection, dead cells and PB/CSC retrovirus complexes are removed by Ficoll density centrifugation (2000 rpm, 20 min, low break).
11. Cells of the interface are harvested and resuspended in five times the volume of PBS and centrifuged (1400 rpm, 5 min).
12. Cells are cultured at 1.5×10^6^ cells/ml cell density in basic HoxB8 cell culture medium supplemented with growth factors and E2 in cell culture dishes of the appropriate size (see below 1.1.2.3.3, step 1).

*Note: Optional, filter retrovirus supernatant through 45 μm cellulose acetate filter (Whatman) to ensure complete removal of HEK293T cells. This might, however, reduce virus titer when retrovirus particles get trapped in cellulose acetate filter.

**Note: HoxB8 retrovirus from one 6 cm tissue culture dish (equivalent to 10 ml HEK293T cell supernatant from two harvests) is sufficient for infection of 3×10^6^ mouse BM cells. Infection efficiency is routinely 10–20%.

###### 1.1.2.3.3 Growth of multipotent HoxB8 progenitors (HoxB8 MPP).

**Figure 2.**
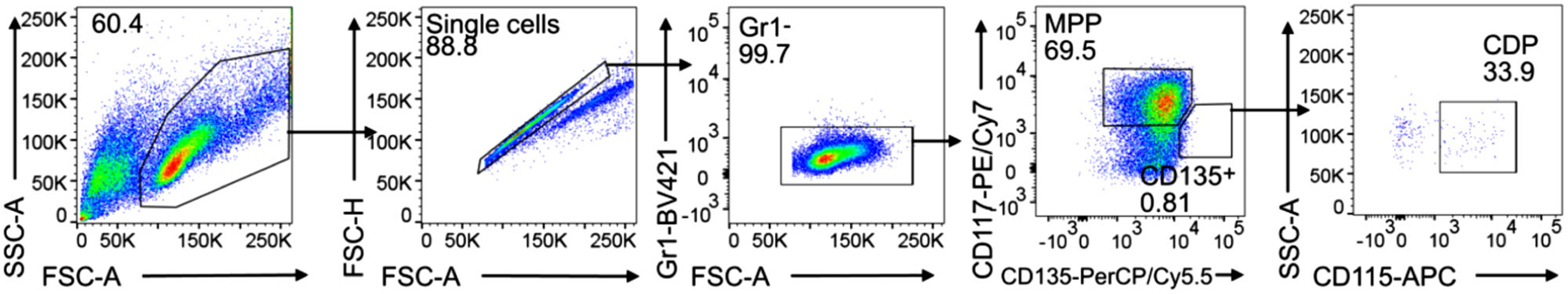
Representative flow cytometry analysis of HoxB8 MPP. HoxB8 MPP were cultured in basic HoxB8 culture medium and subjected to flow cytometry analysis with specific antibodies. MPP: Gr1^−^ CD117^+^ CD135^−^; CDP: Gr1^−^ CD117^int/low^ CD135^+^ CD115^+^.

1. HoxB8 MPP are cultured at 1.5×10^6^ cells/ml cell density in basic HoxB8 cell culture medium supplemented with growth factors (100 ng/ml SCF, 25 ng/ml Flt3L, 25 ng/ml hyper-IL-6, and 40 ng IGF-1) and 10^−6^ M E2 (referred to as HoxB8 growth medium) in cell culture dishes of the appropriate size ***. The major cell population in HoxB8 growth medium is Gr1^−^ CD117^+^ CD135^−^ MPP, and a minor population of Gr1^−^ CD117^int/low^ CD135^+^ CD115^+^ CDP (Fig. 2).
2. HoxB8 MPP can be cryopreserved in FCS: DMSO = 9:1 (v/v) and stored at liquid nitrogen. Following cryopreservation and thawing HoxB8 MPP are kept for one day at high cell density (1.5–2.5 × 10^6^ cells/ml) in HoxB8 growth medium as above, and cells are then further cultured at 1.5 × 10^6^ cells/ml cell density.
3. Perform full medium change every 24 h and adjust cell density to 1.5×10^6^ cells/ml. Briefly, count cells (CASY cell counter and analyzer system), centrifuge at 1400 rpm for 5 min, and resuspend cell pellet in fresh HoxB8 cell growth medium at 1.5×10^6^ cells/ml (e.g. 9 × 10^6^ cells in 6 ml per 6 cm tissue culture dish).
4. To induce HoxB8 MPP commitment toward common dendritic cell progenitor (CDP), HoxB8 cells are cultured in HoxB8 culture medium with growth factors (SCF, Flt3L, hyper-IL-6, and IGF-1) but without E2 at 1.5×10^6^ cells/ml cell density.
5. HoxB8 MPP and CDP are analyzed by flow cytometry. Prior to flow cytometry analysis of MPP/CDP, cells are cultured in basic HoxB8 culture medium (without growth factors and E2) for 1.5–2.0 h to prevent Flt3 and Kit receptor internalization. Then 1 × 10^5^ cells are harvested and stained with specific anti-bodies and 5 × 10^4^ cells are measured. Stain with specific anti-bodies (Table 1) for 20–30 min at +4°C protected from light. The antibodies for flow cytometry are used as 1:400 dilution for cell staining, or alternatively the optimal concentration is determined by antibody titration.
  - HoxB8 MPP grow vigorously, and attention should be paid to regular medium changes to avoid high cell density and exhaustion of culture medium. Intervals of 30–40 h are fine but avoid medium changes beyond 40 h.
  - Discard cells that are not needed!! Avoid high cell densities!!
  - Growth factors are kept at +4°C for daily use and should not be stored longer than 4 weeks. Vortex gently and centrifuge before use. Alternatively, growth factors are snap frozen in liquid nitrogen immediately after use and stored at −80°C. Avoid multiple thawing/freezing cycles (more than 5–6), since this will result in loss of growth factor activity.
  - Cells can be maintained under growth conditions for more than 2–3 months and are essentially immortal with E2. However, long culture and multiple passages might affect their differentiation capacity.
  - HoxB8 MPP are larger than BM cells. Initially, early after the retrovirus infection cultures are a mixed cell population of BM cells and HoxB8 MPP and an extended culture period (at least 20 days) is necessary to get HoxB8 MPP only.
  - HoxB8 MPP cultures contain only a very minor cell population of CDP. A higher CDP frequency is obtained by removing E2 from HoxB8 growth medium (also called spontaneous differentiation; see below Fig. 5A). CDP frequencies increase with time of spontaneous differentiation [5].
  - HoxB8 MPP can be cryopreserved at low passage number (5–7 passages) as polyclonal cell lines. These early passage aliquots are then thawed, expanded, and used for further studies, such as CRISPR/Cas9 genome editing and DC differentiation.

###### 1.1.2.3.4 Sub-cloning of HoxB8 MPP by limiting dilution.

Frequently, HoxB8 MPP are maintained and studied as polyclonal lines, which is sufficient for addressing many scientific questions. Other studies require clonal HoxB8 MPP populations in order to obtain clones with for example optimal differentiation potential. In addition, frequently gene-targeted modification of HoxB8 MPP by CRISPR/Cas9 genome editing requires clonal cell populations, where all cells contain the very same gene modification.

To obtain clonal HoxB8 MPP, it is recommended to sub-clone HoxB8 MPP early (low passage number, 5–7 passages) after their establishment by single-cell culture. Routinely, the singlecell cloning efficiency of HoxB8 MPP by fluorescence-activated cell sorting (FACS) can be rather low, and thus clonal HoxB8 MPP are obtained with higher efficiency by conventional limiting dilution cloning. Aim at the isolation of 24–48 HoxB8 MPP clones and use 3–5 HoxB8 MPP clones for further and detailed analysis.

####### 1.1.2.3.4.1 Sub-cloning of HoxB8 MPP by limiting dilution

**Figure 3.**
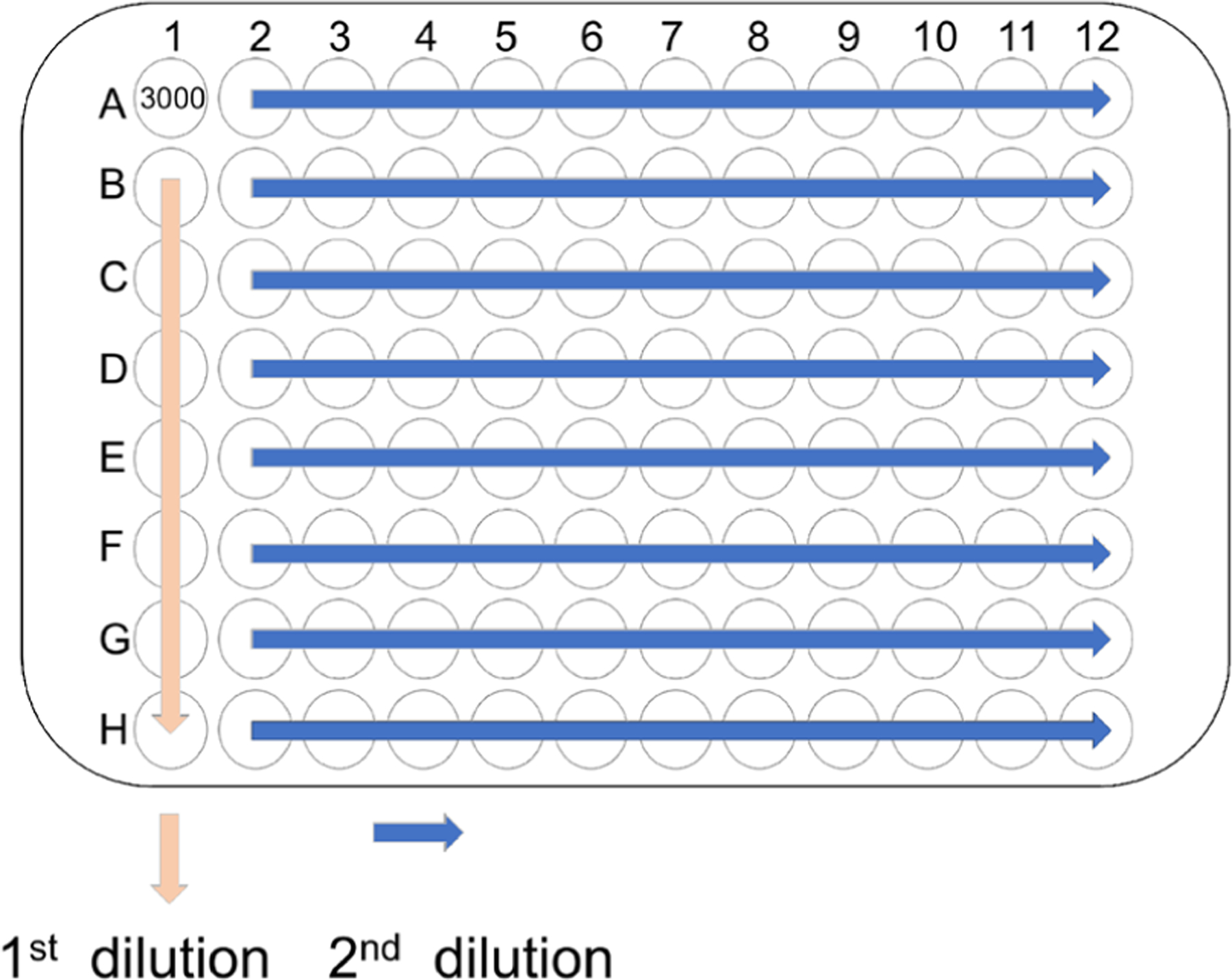
Schematic representation of HoxB8 MPP cloning by limiting dilution.

The schematic representation of the limiting dilution assay is shown in Fig. 3.

1. Prior to setting up the limiting dilution assay perform Ficoll purification of HoxB8 MPP to remove cell debris and dead cells.
2. Add 100 μl HoxB8 growth medium to each well of a U-bottom 96-well plate, except to well A1.
3. Prepare 15,000 cells/ml HoxB8 MPP suspension.
4. For the first series of dilutions add 200 μl cell suspension (3,000 cells in total) to A1, mix gently and transfer 100 μl from A1 to B1, mix gently and transfer 100 μl from B1 to C1, and so on, discard the final 100 μl cell suspension from H1.
5. For the second dilution use a 100 μl multiple-channel pipette to transfer 100 μl cell suspension from line 1 to line 2, gently mix line 2 and transfer 100 μl cell suspension from line 2 to line 3, and so on. Return the final 100 μl of cell suspension from line 12 to line 1.
6. Centrifuge 96-well plate at 1400 rpm for 4 min.
7. Monitor 96-well plate by phase-contrast microscopy and mark wells, which contain single cells.

####### 1.1.2.3.4.2 Expansion of HoxB8 MPP clones

**Figure 4.**
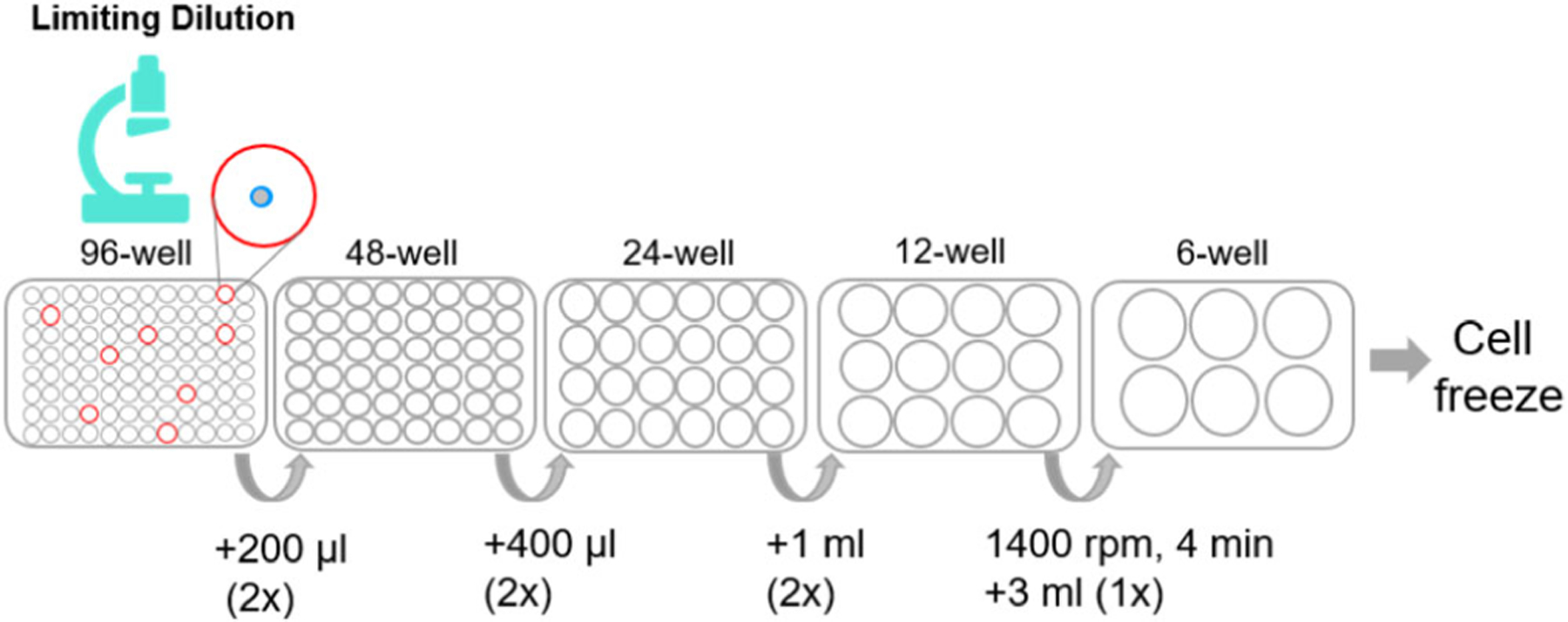
Schematic representation of HoxB8 MPP expansion after cloning by limiting dilution.

The schematic representation for HoxB8 MPP expansion is shown in Fig. 4.

1. Add 50 μl HoxB8 culture medium with 3× growth factors and E2 to single-cell wells on day 4 after limiting dilution. Mark cells that do not grow.
2. Add 50 μl HoxB8 culture medium with 4× growth factors and E2 to the wells that have viable cells on day 7.
3. Check the cells every day, transfer cells from 96-well plate to 48-well plate, 24-well plate, 12-well plate, and 6 well-plate for cell expansion*.
4. Measure HoxB8 MPP numbers in regular time intervals when clonal cell populations expand.
5. Frequently, individual HoxB8 MPP clones show differences in growth and differentiation potential, and thus HoxB8 MPP clones with the desired properties need to be selected. Clonal variation in an inherent property of HoxB8 MPP clones and thus several individual HoxB8 MPP clones (3–5) need to be isolated and studied.
6. Cryopreservation of HoxB8 MPP clones follows standard procedures (FCS: DMSO = 9:1 (v/v)).

*Note: Transfer cells to larger wells as soon as possible by washing the well with HoxB8 cell growth medium. The recommend volumes of HoxB8 MPP culture medium for transfer (Fig. 3) are as follows:

96-well to 48-well, 200 μl basic HoxB8 culture medium with 2× growth factors and E2;

48-well to 24-well, 400 μl basic HoxB8 culture medium with 2× growth factors and E2;

24-well to 12-well, 1 ml basic HoxB8 culture medium with 2× growth factors and E2;

12-well to 6-well, take out cell suspension, centrifuge (1400 rpm, 4 min), and resuspend cell pellet in 3 ml HoxB8 growth medium.

###### 1.1.2.3.5 HoxB8 MPP differentiation into DC.

Multipotent progenitors (MPP) differentiate into DC committed common DC progenitors (CDP) and further into the classical DC subsets cDC1 and cDC2, and into pDC (Fig. 1). HoxB8 MPP differentiation into DC is induced in two ways: (i) by spontaneously DC differentiation upon withdrawal of E2 and (ii) by withdrawal of E2 and addition of Flt3L, referred to as Flt3L-driven DC differentiation.

####### 1.1.2.3.5.1 Spontaneously DC differentiation of HoxB8 MPP

**Figure 5.**
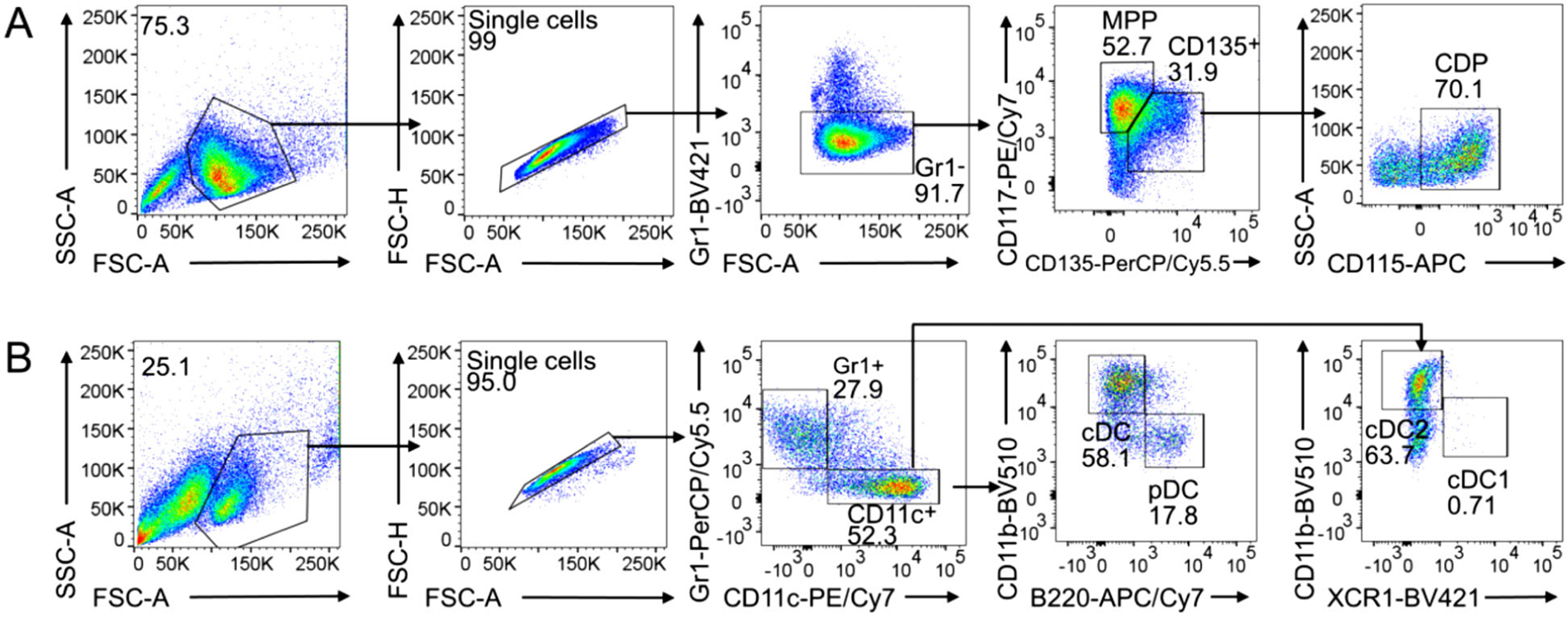
Representative flow cytometry analysis of HoxB8 MPP differentiation into CDP and DC subsets. (A) HoxB8 MPP are cultured in HoxB8 growth medium without E2 for 3 days and analyzed by flow cytometry (section 1.1.2.3.3, step 5). MPP: Gr1^−^ CD117^+^ CD135^−^; CDP: Gr1^−^ CD117^int/low^ CD135^+^ CD115^+^. (B) HoxB8 MPP are cultured in HoxB8 growth medium without E2 for 8 days and analyzed by flow cytometry (see below section 1.1.2.3.5.2, step 7). cDC1: Gr1^−^ CD11c^+^ CD11b^low/−^ XCR1^+^; cDC2: Gr1^−^ CD11c^+^ CD11b^+^ XCR1^−^; pDC: Gr1^−^ CD11c^+^ CD11b^−^ B220^+^.

HoxB8 MPP differentiate spontaneously simply by removing E2 from HoxB8 growth medium and further culture with growth factors (100 ng/ml SCF, 25 ng/ml Flt3L, 25 ng/ml hyper-IL-6, and 40 ng IGF-1) but without E2 at 1.5 × 10^6^ cells/ml cell density. HoxB8 MPP-derived CDP (Fig. 5A) and DC subsets cDC1, cDC2, and pDC are obtained (Fig. 5B).

Spontaneous DC differentiation yields MPP and CDP after 3 days and cDC1, cDC2, and pDC after 8 days (Fig. 5A and B, respectively). For cDC1, cDC2, and pDC generation Flt3L-drive differentiation is much more efficient (see 1.1.2.3.5.2).

####### 1.1.2.3.5.2 Flt3L-driven DC differentiation of HoxB8 MPP

Routinely, HoxB8 MPP are induced to differentiate into cDC1, cDC2, and pDC by removing the growth-promoting cytokines and E2, and adding high concentration of Flt3L (50 ng/ml, Fig. 1). Flt3L-driven DC differentiation is the same for both HoxB8 MPP bulk cultures and for individual HoxB8 MPP clone. HoxB8 MPP clones can differ in DC differentiation capacity and DC subset composition and thus multiple HoxB8 MPP clones need to be analyzed for consistent results. This is particularly important when comparing HoxB8 MPP clones from KO and WT mice or following genetic manipulations, e.g., by CRISPR/Cas9 gene editing.

The protocol includes a transition phase with full growth factors (SCF, Flt3L, hyper-IL-6, and IGF-1) but reduced E2 and high Flt3L (Fig. 1) to ensure a smooth transition from HoxB8 MPP cell proliferation to cell cycle arrest and DC differentiation. This is important, since omitting the transition phase can cause massive cell death and thus compromises DC yield.

1. Collect HoxB8 MPP by centrifugation (1400 rpm, 5 min), count cells, and adjust cell density to 0.75×10^6^ cells/ml.
2. Resuspend cells in HoxB8 medium with growth factors (SCF, Flt3L, hyper-IL-6, and IGF-1) plus Flt3L (Peprotech, 50 ng/ml final concentration) and 10^−8^ M E2 (1:100 of E2 used for growth of HoxB8 MPP), referred to as DC differentiation transition phase medium.
3. Culture cells in DC differentiation transition phase medium in 12-well plates (2 ml/well) for 2 days for DC differentiation in kinetics studies*.
4. Collect HoxB8 MPP by centrifugation (1400 rpm, 5 min), count cells, and wash once with PBS.
5. Resuspend cells in HoxB8 medium without growth factors and without E2, and culture in 12 well plate (2 ml per well) with Flt3L (50 ng/ml, Peprotech) (also called DC differentiation medium), referred to as DC differentiation day 0.
6. Perform partial medium change at days 3 and 6 of differentiation and culture until day 9. Therefore, carefully remove about 1.5 ml old medium from 12-well plate without disturbing cells. Replace with 2 ml fresh medium containing 50 ng/ml Flt3L (DC differentiation medium).
7. DC subsets are analyzed by flow cytometry. Briefly, 1 × 10^5^ cells are harvested and stained with specific antibodies and 5 × 10^4^ cells are measured. Stain with specific antibodies (Table 1) for 20–30 min at +4°C protected from light. The antibodies for flow cytometry are used with 1:400 dilution and MHCII staining is with 1:4000 dilution, or optimal concentrations as determined by antibody titration.

**Figure 6.**
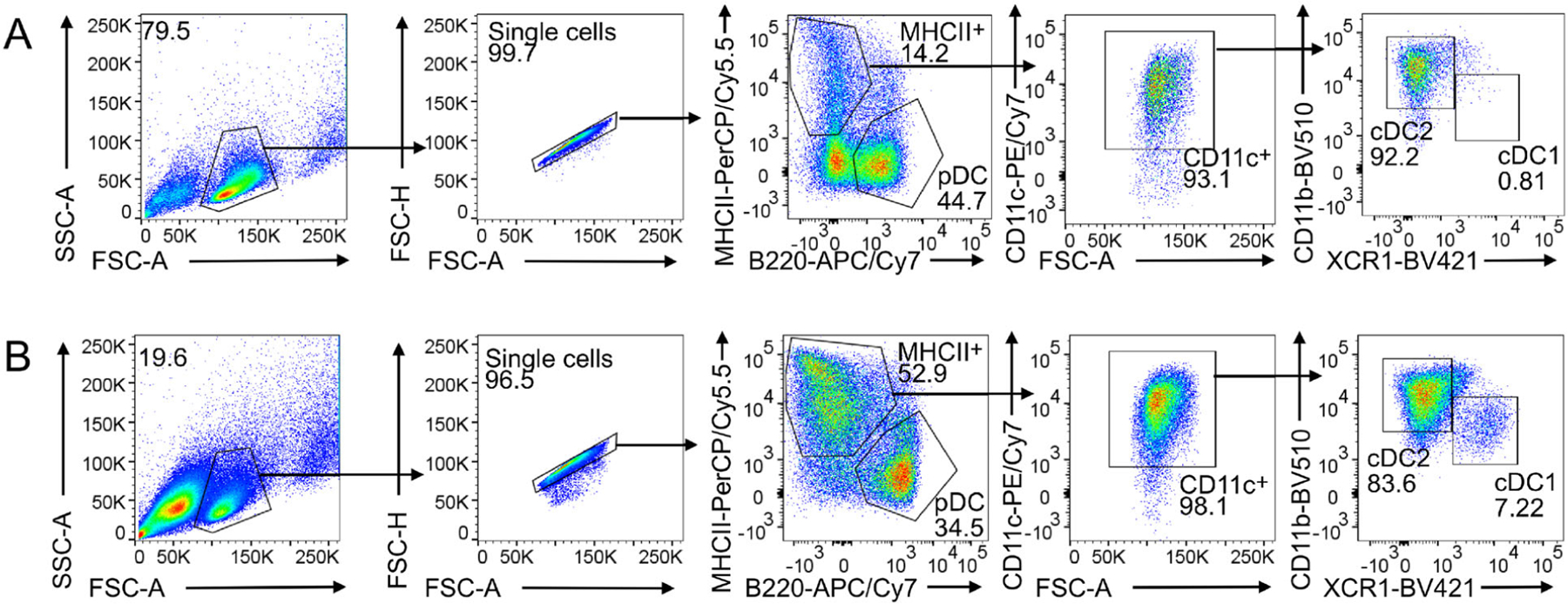
HoxB8 MPP differentiate into cDC1, cDC2, and pDC. HoxB8 MPP were differentiated into DC with Flt3L for 5 and 9 days (panels a and B, respectively) and analyzed by flow cytometry. cDC1: CD11c^+^ CD11b^low/−^ XCR1^+^; cDC2: CD11c^+^ CD11b^+^ XCR1^−^ and MHCII^low/−^ B220^+^ pDC. Representative flow cytometry analysis is shown. MHCII^high^ B220^−^ CD11c^+^ cDC and MHCII^low/−^ B220^+^ pDC were separated and MHCII^high^ CD11c^+^ cDC were further divided into CD11b^low/−^ XCR1^+^ cDC1 and CD11b^+^ XCR1^−^ cDC2.

**Figure 7.**
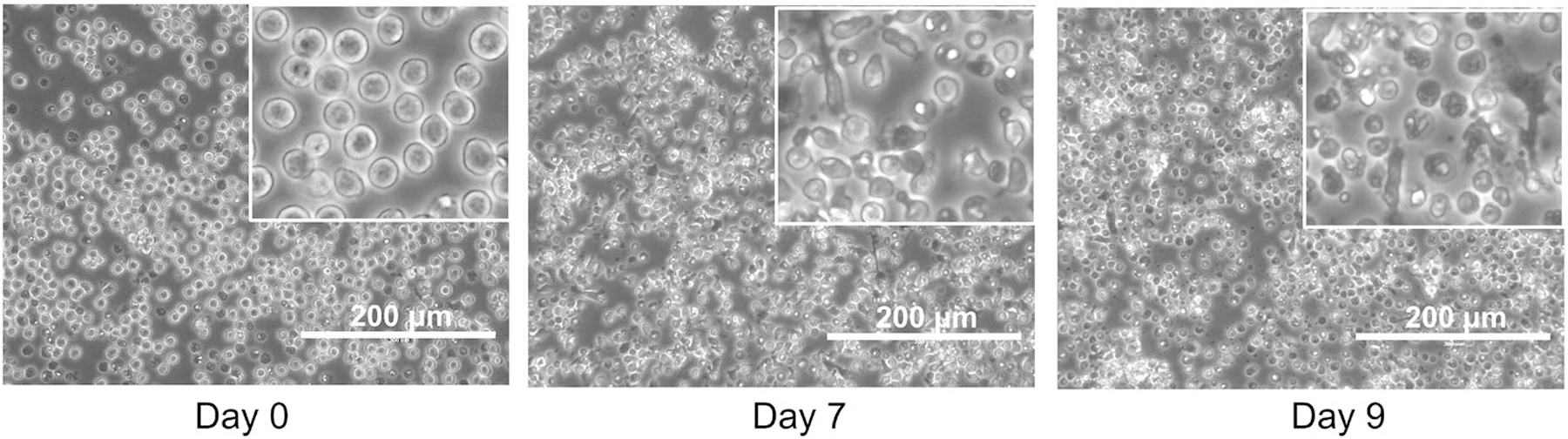
Representative phase contrast image of Flt3L-driven DC differentiation of HoxB8 MPP on days 0, 7, and 9. Scale bar: 200 μm.

Routinely, at day 9 of differentiation, cDC1, cDC2, and pDC frequencies are 3–7%, 37–44%, and 29–48%, respectively [5]. A representative flow cytometry analysis of DC subsets of Flt3L-driven DC differentiation at days 5 and 9 is shown in Fig. 6. Representative phase contrast images at days 7 and 9 are in Fig. 7.

*Notes: Prepare individual wells for following up on DC differentiation in kinetics studies e. g. by flow cytometry. Cells will keep on growing in the first two days of reduced E2 culture and thus if e.g. 4 measurements are required in a kinetics study, start from 2 wells of HoxB8 MPP (0.75×10^6^ cells/ml, 1.5×10^6^ cells in total per well of 12-well plate), expand the cells and adjust cell density to 0.75 × 10^6^ cells/ml until differentiation day 0, then you should have enough cells for four measurements.

For scaling up DC production larger tissue culture plates are used (e.g., 6-well plates, 10 cm dishes).

##### 1.1.2.4 Data analysis.

Flow cytometry data are analyzed using FlowJo V10 (BD Biosciences).

##### 1.1.2.5 Pitfalls.

###### Problem: Low DC differentiation potential

####### Potential solutions:

HoxB8 MPP were cultured for too long (2–3 months) and accumulated high passage numbers. Use HoxB8 MPP with a lower passage number.

###### Problem: Low yield of the DC subset of interest

####### Potential solutions:

Perform kinetics for bulk HoxB8 MPP differentiation culture to determine the optimal time point for your DC subset of interest. For example, pDC are obtained at day 5 of DC differentiation and cDC1 require 8–9 days of DC differentiation.

Analyze various HoxB8 MPP clones, since their propensity for DC differentiation can vary, and determine their DC differentiation kinetics.

###### Problem: Low number of single cell colonies per 96-well plate by limiting dilution.

####### Potential solutions:

To increase the number of single-cell colonies, mark also the wells that have no cell, 1, 2, 3, and 4 cells, then mix the wells that have more than 1 cell with the no cell wells, spin down the cells and recheck for single-cell wells and select those for further study.

##### 1.1.2.6 Top tricks.

HoxB8 MPP are sensitive to low temperature, and cell viability will dramatically decrease when HoxB8 MPP are put on ice. HoxB8 MPP should be kept at room temperature. However, there is no problem with cryopreservation of HoxB8 MPP.

### 1.2 Mouse DC generation and quality control

#### 1.2.1 Generation of murine BM-derived MoDC with GM-CSF (BM-MoDC)

##### 1.2.1.1 Introduction.

The method to generate DC from bulk BM cell suspensions using the GM-CSF growth factor that carries the terms granulocyte-macrophage in its name indicates the expansion of these cells, more specifically neutrophils and macrophages as initially described, but DC were not known at this time [11]. Years later, the additional appearance of DC was observed in such murine GM-CSF cultures [12]. We then modified the classical BM-DC Inaba protocol to reveal higher cell yields [13].

The generation of DC from BM cells has been shown for mice [12], rats [14], and humans [15] and meanwhile for many more species [16]. The original protocols all used combinations of high doses GM-CSF plus IL-4 for their cultures. For rat and human BM-DC generation the use of IL-4 appears mandatory because in its absence only macrophages develop [13]. When we investigated the role of IL-4 for murine DC cultures, we found that in cultures with high doses of GM-CSF the addition of IL-4 does not have an effect on cell yield but generated an additional subset resembling epidermal Langerhans cells [17]. The macrophage and DC composition of such GM-CSF BM cultures has been further dissected by others and indicated that mainly expanding MDP and cMoP generated CD11c^+^ MHC II^+^ cells in GM-CSF cultures. Gating only on the CD11c^+^ CD11b^+^ MHC II^high^ cells resulted in 3 populations that were segregated by their differential CD115 (M-CSFR) and CD135 (Flt3) expression as CD115^−^ CD135^−^ DN-DC and CD115^−^ CD135^+^ GM-DC and a CD115^+^ CD135− bona fide DC population that was not further investigated. Few CDP generated also few cDC1 in their cultures, which we can confirm here by XCR1 staining (Fig. 10 below). Moreover CD11c^+^ MHC II^low^ cells were identified as macrophages without the potential to develop into CD11c^+^ MHC II^high^ mature DC [18]. The latter is in contrast to our experiments where CD11c^+^ MHC II^low^ cells showed bipotent capacity to develop into macrophages but also into CD11c^+^ MHC II^high^ mature DC [17].

**Figure 8.**
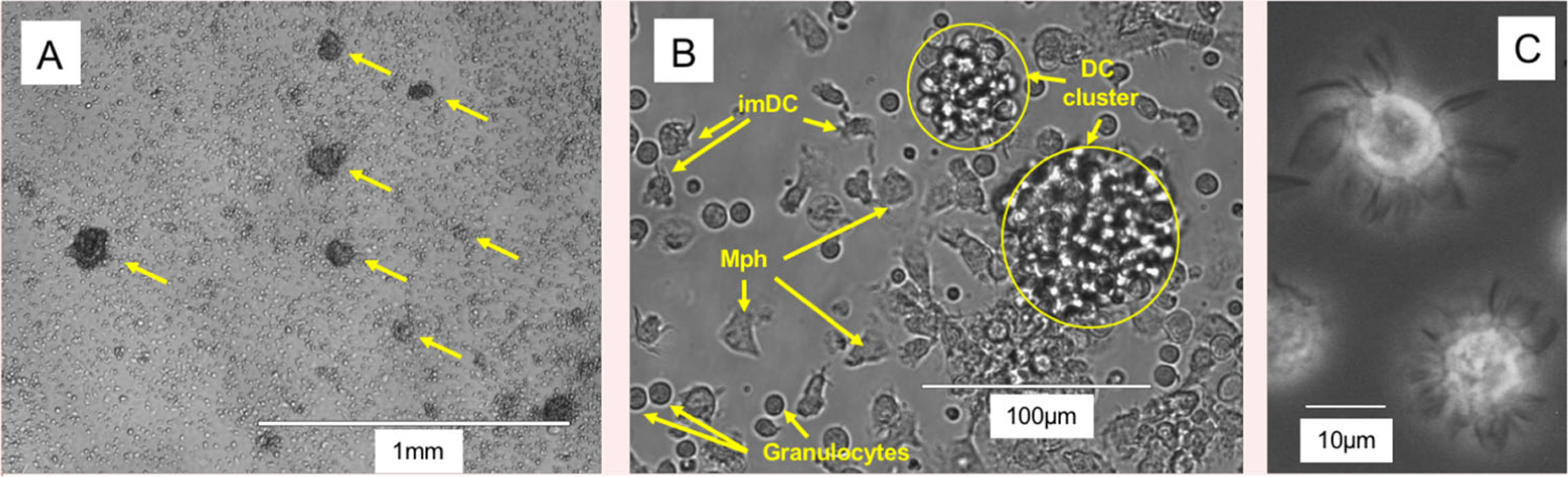
Phase contrast microscopy images of BM-MoDC cultures with GM-CSF at day 8. (A) Cultures show cluster formation of proliferating progenitors and developing immature DC (arrows). (B) Cluster formation besides adherent macrophages, round cells with smooth surface presumably representing neutrophilic granulocytes, and immature DC appearing with few spine-like surface protrusions and mature DC with several protrusions. (C) Suspension cells representing spontaneously matured BM-MoDC with many veil-like surface protrusions.

**Figure 9.**
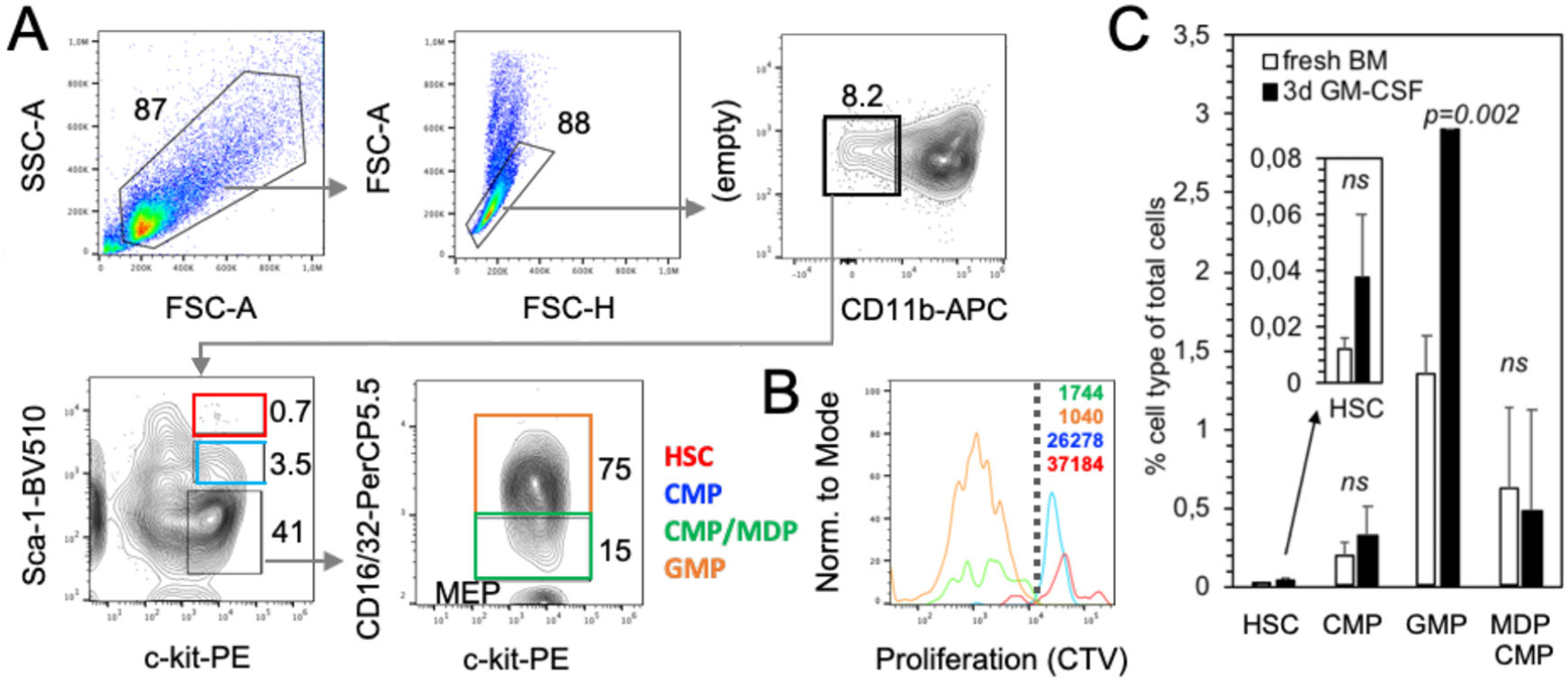
GMP are the major expanding cell type in GM-CSF cultures. Fresh BM cells were labeled with the proliferation dye Cell Trace Violet (CTV) and either analyzed directly or after 3 days of GM-CSF culture by flow cytometry. (A) Gating strategy of GM-CSF cultured cells. Staining with Sca-1, c-kit (CD117) and CD16/32 allowed further gating for hematopoietic stem cells (HSC), common DC progenitors (CDP), granulocyte macrophage progenitors (GMP) or monocyte DC progenitors (MDP). Percentages of gates are indicated. (B) Example staining of CTV fluorescence after 3 days of GM-CSF culture indicates preferential GMP proliferation. CTV dilution was analyzed by FACS and GEO-MFI values are indicated. Dotted line separated proliferating from non-proliferating cells. (C) Frequencies of fresh BM or 3d GM-CSF cultured cells were calculated on the basis of FACS analyses and gated as in (A). *n* = 4 independent experiments. Insert shows rare HSC at a different scale.

Adherent DC cluster formation in the cultures was observed microscopically (Fig. 8), while neutrophilic granulocytes cluster earlier during culture (day 4–6) and remain in suspension. As suggested by the granulocyte and macrophage stimulating capacity of GM-CSF, we find that GMP are the major expanding progenitor, but not HSC, CDP, or MDP (Fig. 9), while GMP were not investigated in the previous study [18]. MDP generation of monocytes was supported by a recent study [19], while others found that GMP are the major cell type generating monocytic cells [20]. Moreover, also the GMP appear to be a heterogeneous cell population with pre-formed and restricted developmental potential at least for neutrophils [21]. Thus, there is still controversy on the progenitors generating MoDC in such cultures [22, 23], which requires further investigations.

**Figure 10.**
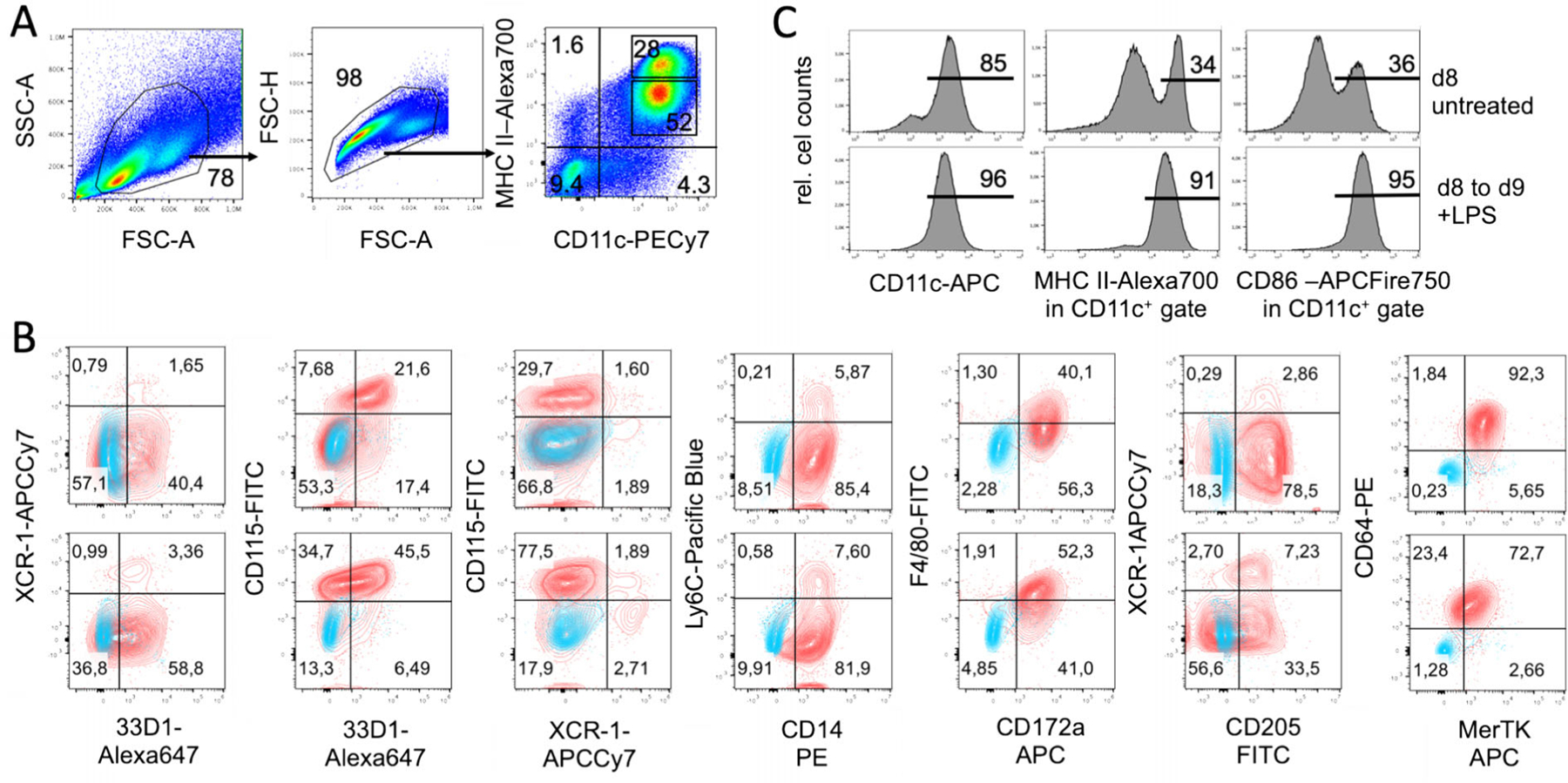
Cultures of d8 GM-CSF BM cultures contain immature and spontaneously matured MoDC. Further maturation by LPS. (A) D8 GM-CSF BM cultures were analyzed by flow cytometry and gated according to FCS and SSC parameters as indicated and stained for MHC II and CD11c. (B) Cells were pre-gated as shown in (A). Upper row represent data from CD11c^+^ MHC II^high^ gated mature DC and lower row CD11c^+^ MHC II^low^ gated immature DC. Surface staining of the indicated marker with quadrant statistics in red is overlaid with the FMO staining in blue. (C) Separate FACS analysis of the d8 cultures with immature CD11c^+^ MHC II^low^ CD86^low^ and spontaneously matured CD11c^+^ MHC II^high^ CD86^high^ DC. To obtain mature DC transfer at d8 to fresh dishes/wells and addition of proinflammatory cytokines or TLR ligands (here 100 ng/ml LPS) is recommended.

**Figure 11.**
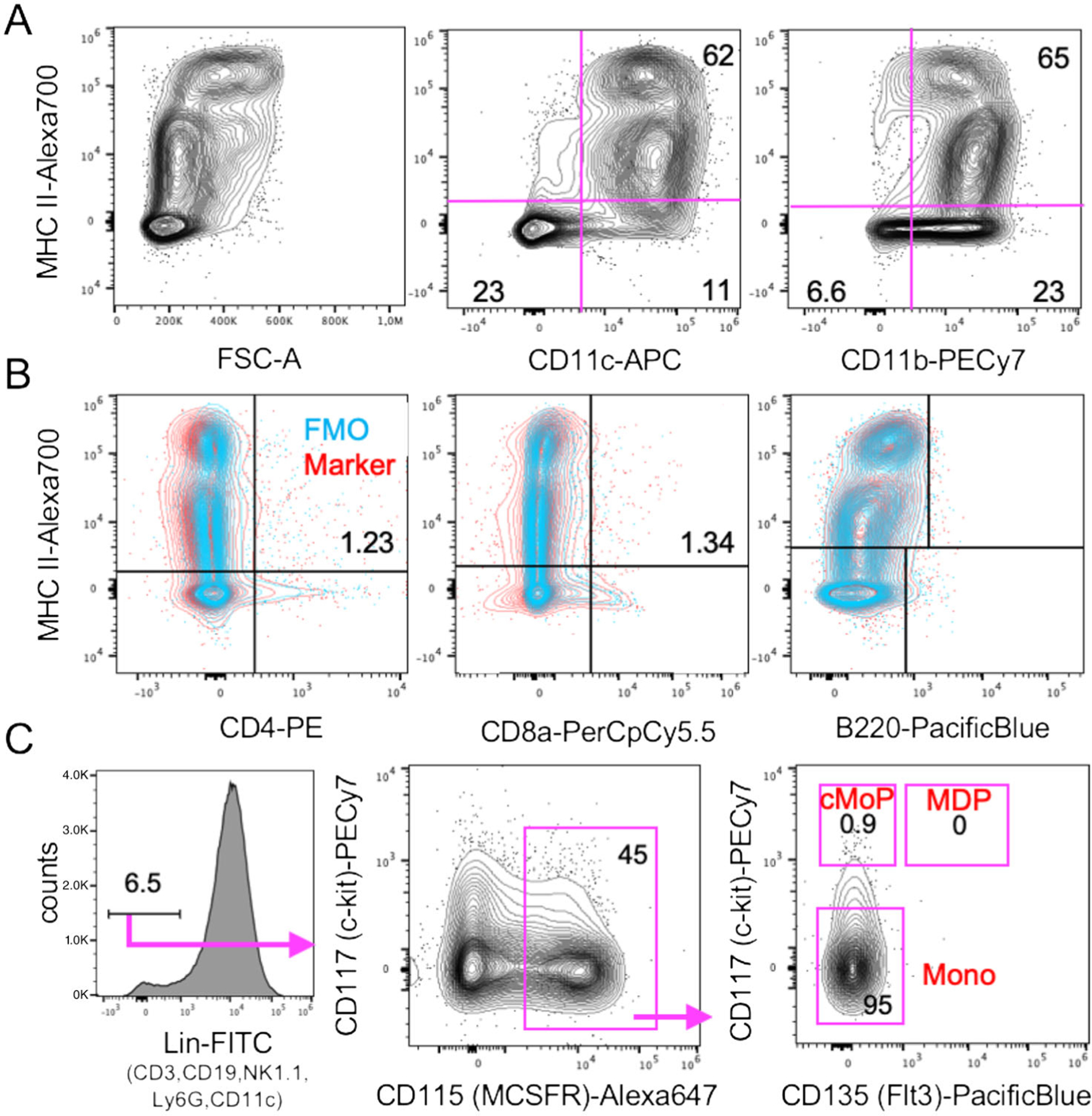
Contaminating monocytes but only a few myeloid progenitors and no T and B cells in day 8 GM-CSF BM cultures. All data pre-gated as shown in Fig. 10A for FSC-A/SSC-A and single cells. (A) Surface staining was performed for the indicated markers. Mature MHC II^high^ cells are larger (FSC-A), express similar levels CD11c but lower CD11b. (B) Surface staining of the indicated markers in red is overlaid with the FMO staining in blue. (C) For surface staining of myeloid progenitor markers, a lineage marker exclusion was performed to identify MDP, cMoP, and differentiated monocyte (Mono) subsets.

Although the generation of cDC in GM-CSF BM cultures from CDP has been proposed [18], their overall contribution to the final DC yield remains low. Only *<*2% XCR1^+^ CD11c^+^ MHC II^+^ cells (of total cells, data not shown) that would refer to cDC1 can be found in the cultures at d8 besides other contaminants (Figs. 10 and 11).

Our data suggest, that most of the DC in GM-CSF cultures develop from expanding GMP (Fig. 9) via Ly6C^high^ classical monocytes [24]. Others found that both sorted and GM-CSF cultured Ly6C^high^ and Ly6C^low^ monocytes developed into CD11c^+^ MHC II^high^ and CD11c^+^ MHC II^low^ cells, although with low efficacy [23]. The low efficacy may result from the fact that differentiated monocytes do not proliferate anymore and only a 1:1 conversion can occur. Recently, a population of XCR1^−^ 33D1^−^ CD115^+^ MoDC was induced in the spleen of mice injected with GM-CSF [25], a phenotype that only partially matches our in vitro generated DC, where the majority among both immature MHC II^low^ and mature MHC II^high^ DC exhibits an XCR1^−^ 33D1^+/−^ CD115^+/−^ phenotype (Fig. 10A–C). The expression of other markers strongly argues for their identity as CD11b^+^ CD172a/SIRPα^+^ F4/80^low^ Ly6C^neg^ CD14^+^ CD64^+^ MerTK^+^ MoDC. Other typical markers for cDC1 (CD205) or cDC2 (33D1) are expressed partially or on a subset (Figs. 10B and 11A).

With these data, and for the fact that "BM-DC" can be also generated with Flt3L [26], we will term the GM-CSF generated DC from GMP and monocytes in the following BM-MoDC. Of note, monocytes and macrophages are also present in GM-CSF cultures and BM-MoDC maintain many markers and characteristics of monocytes and macrophages. However, BM-MoDC also acquire many functional features that are shared with but also different from cDC subsets in vitro and in vivo and as well as in mice and humans [13, 25, 27–32].

##### 1.2.1.2 Materials.

- GM-CSF: We use GM-CSF-containing supernatant in most of the cases (see below). Alternatively, or when specific doses are needed we use murine rGM-CSF from Peprotech at 200 U/ml = 40 ng/ml (not 20 ng/ml as miscalculated in our paper [33]).
- GM-CSF-supernatant: murine GM-CSF producing cells [34] cultured in R10, this supernatant is filtered (to avoid cell transfer) and used at 10% as a source for GM-CSF described in [33]. The produced supernatant contains 400–800 U/ml GM-CSF tested by ELISA. The cell line can be provided by Manfred Lutz.
- Preparation of RPMI + 10% FCS cell culture medium (R10):
  - 500 ml RPMI 1640 medium (various suppliers)
  - + l-Glutamin (2 mM) (Sigma, G-7513)
  - + Penicillin-Streptomycin combined solution (100 U/ml / 100 μg/ml; Sigma, P-4333)
  - + ß-Mercaptoethanol (50 μM) (Sigma, M-7522).
  - + 50 ml fetal calf serum (FCS), heat inactivated for 30 min at 57°C, and then sterile filtered (0.45μm) to remove clump material leading to DC activation.
- Mouse strain: C57BL/6 female, 4–6 weeks old, same for BALB/c and most other strains.
- Petri dishes, 10 cm (e.g., FALCON #351029 or GREINER #664102), NOTE: bacterial quality dishes yield somewhat (≈10%) less macrophages and more DC as compared to tissue culture quality dishes.

##### 1.2.1.3 Step-by-step sample preparation.

###### Culture:

Count BM cells and culture 2–3 × 10^6^ cells per 10 cm dish in 10 ml R10 containing 10% GM-CSF-supernatant or ≥200 U/ml rGM-CSF (R10 + GM-CSF). Incubate at 37°C, in 7% CO_2_.

###### Feeding:

**Day 3**: add fresh medium: 10 ml R10 + GM-CSF

**Day 6**: change half of medium: remove 10 ml old medium (avoiding the aspiration of cells) and add fresh 10 ml R10 + GM-CSF

**Day 8**: use the non-adherent and loosely adherent cells as BM-MoDC (mild rinsing with 10 ml pipets only! Remember that MoDC will mature through pipetting e.g. by disruption of E-cadherin-mediated clustering [35]). Adherent cells remain and can be considered as macrophages.

###### Modifications of the standard protocol for selective immature or mature BM-MoDC generation

The GM-CSF cultures at day 8 also do not generate uniform immature or mature BM-MoDC [33] (Fig. 10A). To obtain higher frequencies of mature BM-MoDC, the cells should be transferred at day 8 from the original culture dish into a fresh dish or well and maturation stimuli, e.g. 100 ng/ml LPS, should be added overnight (16–24 h) (Fig. 10C). Exclusively immature DC can be obtained by adding pharmacological drugs, e.g. dexamethasone [36] or cytokines, e.g. IL-10 [37], throughout the culture period from day 0 to day 8. Many factors and also genetic modifications to obtain *in vitro* generated immature and thereby tolerogenic DC have been reviewed elsewhere [38–41]. Of note, after removal of some of such inhibitors, such as IL-10, from the culture and the subsequent addition of LPS, the DC will still undergo maturation [39]. Other methods or specific factors allow the generation of immature and maturation-resistant BM-MoDC. Since such maturation-resistant DC are also stable *in vivo*, they have been successfully used in tolerogenic therapies in mice, monkeys, and humans [15, 36, 41–45].

##### 1.2.1.4 Data analysis.

Cultures of BM-MoDC should contain 6–10×10^6^ living cells per 10 cm Petri dish after gently rinsing as determined by Trypan Blue staining in a Neubauer counting chamber. Further quality control should include at least a surface flow cytometric staining for CD11c and MHC II to characterize the cells as bona-fide BM-MoDC (CD11c^+^) and MHC II and/or CD86 to determine the relative frequencies of immature (MHC II^low^) and mature (MHC II^high^) BM-MoDC (Fig. 10A,C). More markers help to further identify the cells (Fig. 10B).

##### 1.2.1.5 Pitfalls.

###### Purity

The resulting cells of these bulk BM-MoDC cultures cannot be considered as pure DC populations. Since bulk BM is used as a starting cell source, all hematopoietic lineage cells are present at day 0 and only different stages of GM-CSF responsive myeloid progenitors will proliferate. The GM-CSF responsive cells are also not synchronized in their development into DC, so that different stages of progenitors still remain in the culture at day 8. Due to the use of GM-CSF as a neutrophil and macrophage growth factor these are obvious contaminants. Differentiated macrophages, however, strongly adhere to the tissue culture plastic and neutrophils decline over the culture period. The nonor loosely adherent cells represent mixtures of predominantly immature and mature BM-MoDC but the specialty that the "immature DC" fraction with can still develop into macrophages and not exclusively DC [17, 33] (Figs. 10 and 11).

###### Heterogeneity of "immature DC"

We found that GM-CSF cultures contain MHC II^low^ expressing cells which are regularly termed ‘immature MoDC’ bear the dual capacity to generate both, macrophages and BM-MoDC. Upon sorting of MHC II^low^ cells and seeding to new culture plates, a fraction of MHC II^low^ cells down-regulates surface MHC II and subsequently become adherent macrophages, another fraction remains MHC II^low^ and a third fraction spontaneously matures into MHC II^high^ cells [17]. Moreover, the MHC II^low^ expressing cells are composed of two DC subsets. One appears as typical BM-MoDC, and another showing a Langerhans cell-like phenotype with lower endocytosis capacity and exclusive development into mature DC but not macrophages after sorting and re-culture [17]. Thus, the MHC II^low^ cell fraction is heterogeneous and shows a high plasticity for further development into macrophages.

###### Contaminants

**Figure 12.**
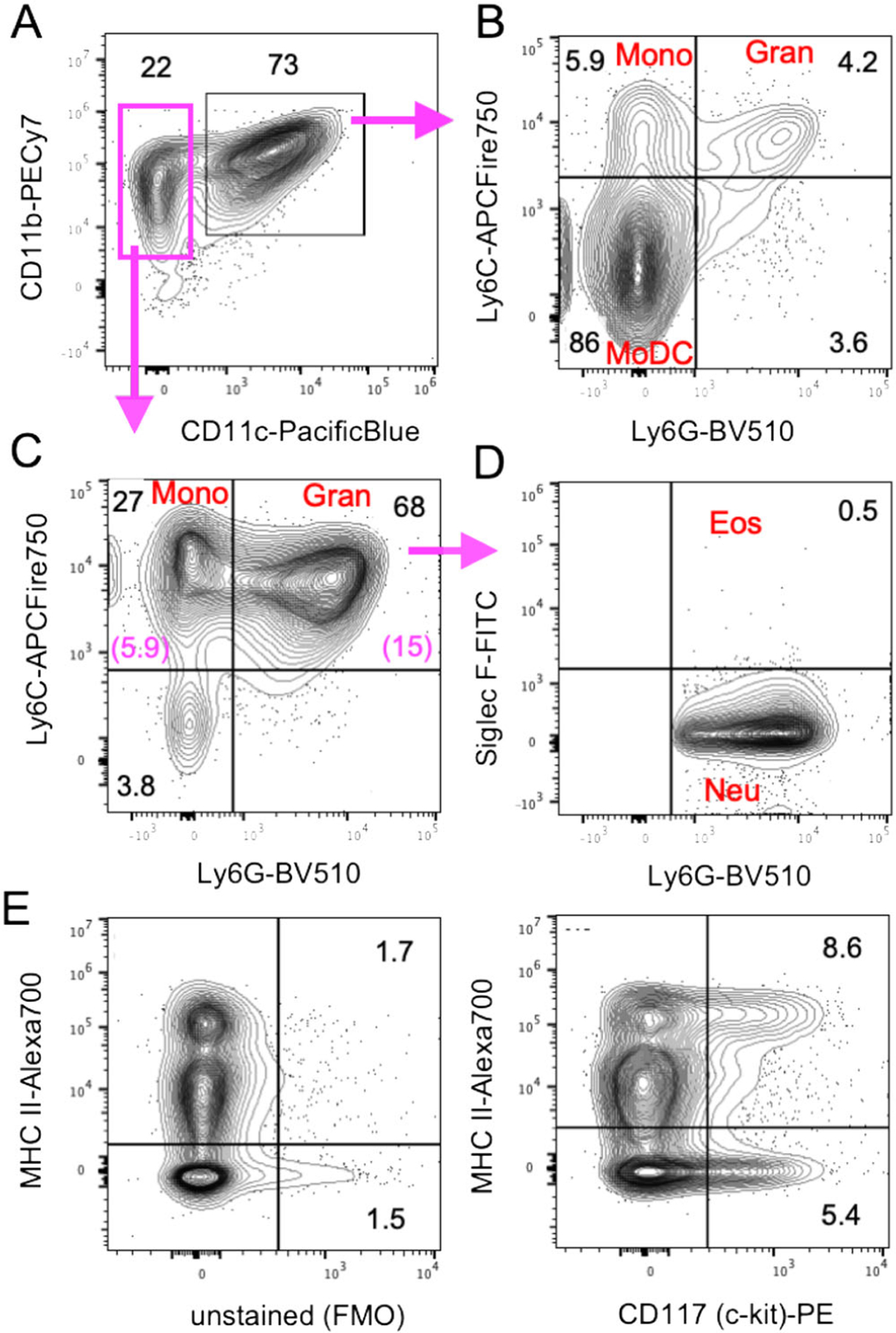
Monocytes and neutrophils represent the major non-DC in the non-adherent fraction of day 8 GM-CSF cultures. (A–D) D8 GM-CSF BM cultures were stained for the indicated markers. Pre-gating was performed like in Fig. 10A. Cell types are indicated in red. Percentages within quadrants are shown in black or are in magenta when calculated for the whole culture cellularity. (E) CD117 expression is detectable on MHC II^neg^ progenitor cells but also on mature MHC II^high^ BM-MoDC.

Macrophages typically adhere and will not be removed by mild pipetting when harvesting the DC at day 8. Thus, contaminants within the suspension cells are as follows. The GM-CSF cultures contain a small fraction of XCR1^+^ cells, indicative for cDC1, as also suggested by others before [18]. In contrast, there is a large overlap of marker expression between BM-MoDC and cDC2, as exemplified here by CD11b, 33D1, and CD172a staining (Figs. 10B and 11A). The day 8 BM-MoDC cultures do not contain CD4^+^ or CD8^+^ T cells, B220^+^ B cells, or B220^+^ pDC as contaminations (Fig. 11C). Due to their GM-CSF responsiveness and high proliferation capacity there are still about 1% of common monocyte progenitors (cMoP) in the cultures and about 5–7% CD11b^+^ CD11c^−^ non-proliferating monocytes (Figs. 11D and 12C). A contamination of BM-MoDC cultures by NK cells has been reported that appeared most prominently in BM cultures from RAG1^−/−^ mice, where minute contaminations of 0.09% NK cells accounted for a substantial IFN-*γ* production after LPS stimulation [46], thus, incorrectly indicating IFN-*γ* production by the BM-MoDC. Neutrophils maximally expand at days 3–5 but slowly die out thereafter. They still represent a major contamination at d8 of around 15% [33] (Fig. 12A–C), but do not contain Siglec F^+^ eosinophils (Fig. 12D).

###### Marker reliability

For analysis of progenitor markers in BM-MoDC cultures it may be of importance to note that CD117 (c-kit) is expressed on MHC II^neg^ cells, and on MHC II^low^ cells and additionally up-regulated upon maturation on MHC II^high^ cells (Fig. 12E) [47].

##### 1.2.1.6 Top tricks.

###### Cytokines

GM-CSF and IL-4 are species-specific cytokines. Therefore, only recombinant mouse cytokines can be used for murine DC generation.

###### Never in the cold

BM-MoDC remains functionally impaired when kept on ice or otherwise in the cold (unlike T and B cells). Thus, to use BM-MoDC for functional assays, they should be kept at room temperature before their in vitro use or before injection.

###### Culture period

With the here presented protocol, optimal DC characteristics are achieved after a culture period of 8 days in the GM-CSF-containing media [33]. After only 3 or 4 days culture period in GM-CSF-containing media, BM-derived granulocytic and monocytic myeloid-derived suppressor cells (MDSC) can be retrieved [48].

###### GM-CSF dose

Doses higher than 200–400 U/ml (depending on supplier quality) do neither lead to a higher yield of DC nor to their improved functionality. However, doses lower than 200 U/ml reduce cell yields and very low doses (5–20 U/ml) generate maturation-resistant immature DC and macrophages [45] accompanied by the co-generation of MDSC in such cultures [48].

###### Addition of IL-4 to murine GM-CSF cultures

The addition of 100 U/ml murine rIL-4 to high doses of ≥200 U/ml murine BM GM-CSF cultures does not alter cell yields or cell quality with respect to MHC II and CD86 expression [45], while human and rat MoDC cultures require addition of IL-4 for DC generation and to prevent macrophage outgrowth [13]. Using IL-4 at lower doses of GM-CSF rescues the otherwise impaired DC maturation potential and antigen presentation [49]. Cultures with high GM-CSF plus IL-4 also support the appearance of Langerhans cell-like DC among the immature and mature DC fractions [17].

###### CO_2_ level

The use of 7% CO_2_ in the 37°C incubator for BM-MoDC culture instead of the usual 5% reveals higher cell yields.

###### FCS

The FCS source can be critical in rare cases and give much higher or lower yields and different rates of spontaneous maturation [50]. Therefore, FCS batches need to be tested before use for BM-MoDC culture. DC grown in FCS also will (cross)-present FCS-peptides on MHC I and II. This is especially important when injected into mice for immunization [51]. Accordingly, the restimulation of T cell responses from such BM-MoDC immunized mice with specific peptides has to be performed in serum-free medium to avoid high background proliferation on FCS. HL-1 medium (BioWhittaker from LONZA, formerly Ventrex) revealed good results for such ex vivo T cell restimulation assays [51]. BM-MoDC can also be generated directly in serum-free medium, however leading to functional abnormalities [51].

#### 1.2.2 Generation of DC from mouse BM using Flt3 ligand

##### 1.2.2.1 Introduction.

In vitro differentiation of DC from murine BM progenitors has been described for decades, primarily by using GM-CSF with or without the addition of IL-4 [12, 52–54]. It is well characterized now that this culture system generates DC subsets that are more like inflammatory or monocyte-derived DC than like splenic steady-state DC, both in terms of phenotype and function [55, 56]. In vivo DC differentiation is dependent on Flt3 ligand (Flt3L) and absence of Flt3L strongly affects their development [57–61]. Therefore, the utilization of Flt3L to differentiate DC in vitro has been a strategy explored. Addition of Flt3L to the culture favors the differentiation of BM cells into immature cDC1, cDC2, and pDC [26, 62–65]. These DC share the expression of surface markers and transcription factors with their steady-state splenic counterpart [26, 64]. Following activation with toll-like receptor (TLR) ligands, these three subsets can mature and initiate an effective immune response [65]. Notably, compared to GM-CSF-derived DC, Flt3L-derived DC are better antigen-presenting cells and have higher migration potential [66].

Differentiation of DC in vitro in the presence of Flt3L is relatively easy and allows the generation of a large number of cDC1, cDC2, and pDC. However, the efficacy and efficiency of this culture system are influenced by several variables. First, it is well appreciated that fetal bovine serum (FBS) can strongly affect Flt3L-induced DC differentiation in terms of numbers generated and of DC subset proportions. Second, Flt3L should be titrated to ensure the use of the optimal concentration to support DC differentiation. Lastly, a kinetic of differentiation should be done. The source of Flt3L and the FBS batch used can change the time point of peak production and the proportion of DC subsets. Moreover, pDC and cDC develop with different kinetics during Flt3L-induced differentiation, with pDC arising faster. Thus, the longer cells are in culture, the less pDC will be harvested [62].

In this section, we provide a detailed protocol on how to generate large numbers of immature cDC1, cDC2, and pDC in vitro that resemble their in vivo steady-state counterparts.

##### 1.2.2.2 Materials.

###### 1.2.2.2.1 Reagents.

A complete list of reagents is provided in Table 3

###### 1.2.2.2.2 Equipment.

Necessary equipment is listed in Table 4.

##### 1.2.2.3 Step-by-step sample preparation.

###### 1.2.2.3.1 Preparation of stocks and solutions.

####### Flt3 ligand stock solution

Dissolve lyophilized powder at a concentration of 25 μg/ml in DMEM containing 0.1% BSA as a stabilizer. Prepare aliquots and store at −80°C. Thaw one aliquot, vortex gently, and centrifuge before use. Aliquots in use can be stored at 4°C for daily use but not longer than 2 weeks.

Remark: human Flt3L can be purchased from alternative vendors and in alternative formats, e.g. recombinant Flt3L, Ig-stabilized Flt3L or supernatant of cell lines stably transfected with Flt3L. Each Flt3L batch should be carefully tested (see below section 1.2.2.5).

####### l-Glutamine and Penicillin/Streptomycin

Thaw 100 ml l-Glutamine and Penicillin/Streptomycin at 37°C in a water bath until all clumps are dissolved. Mix and aliquot in 5 ml aliquots. Store at −20°C. Thaw aliquots right before use.

####### FBS

Thaw the bottle with FBS at 37°C in a water bath. Once completely thawed, mix, prepare 50 ml aliquots, and store aliquots at −20°C. When preparing medium, thaw an aliquot, mix, and then incubate the tube for 30 min in a 56°C water bath (make sure the tube is fully immersed in water) to destroy complement activity by heat inactivation.

####### Complete medium

Prepare complete medium by adding 50 ml heat-inactivated FBS, 5 ml l-Gln, 5 ml Pen/Strep, 0.5 ml 2-mercaptoethanol to 440 ml RPMI 1640.

####### Staining buffer

Prepare PBS containing 0.5% FBS (v/v) and 2.5 mM EDTA.

###### 1.2.2.3.2 Generation of bulk DC subsets from BM with Flt3L.

1. Euthanize mice, harvest BM cells, and determine cell concentration as described in section 1.1.1;
2. Transfer the required number of cells into a 50 ml tube;
3. Spin cells down for 5 min at 300 × *g*;
4. Resuspend the pellet in complete medium supplemented with 50 ng/ml Flt3L, to reach a cell concentration of 2 × 10^6^/ml;
5. Seed cells in 6 well plates, 5 ml per well;
6. Incubate the cells at 37°C and 5% CO_2_;
7. On day 5 perform a partial medium change: replace 4.5 ml old medium by 5 ml warm complete medium supplemented with 50 ng/ml Flt3L;
8. On day 8, repeat step 7;
9. Harvest cells on day 10 by resuspending the non-adherent or loosely adherent cells. Some stromal cells and macrophages may be left in the dish;
10. Determine cell density: mix cell suspension and Trypan Blue solution 1:1 and count using a Neubauer chamber;
11. DC can be analyzed by flow cytometry; DC subsets can be isolated by sorting for mRNA, gDNA or protein isolation, or used in functional assays by culturing them further in complete medium supplemented with 50 ng/ml Flt3L.

General remarks:

- This protocol describes seeding of 10 × 10^6^ cells (5 ml of a 2 × 10^6^/ml cell suspension) per well in a six-well plate, with medium changes on days 5 and 8. To our experience, the number of seeded BM cells can vary without affecting DC differentiation efficiency. However, Flt3L concentration and timing of medium changes should be adapted accordingly. For example, 4.5 × 10^6^ cells per well (3 ml of a 1.5 × 10^6^/ml cell suspension) can be cultured with 20 ng/ml Flt3L for 7 days before performing the first medium change. Cell concentrations higher than 3 × 10^6^/ml should not be used.
- The culture usually generates as many CD11c^+^ DC as the number of total BM cells put in culture at the start. However, the yield will vary in function of the Flt3L and FBS batches used.
- It is important to note that Flt3L and FBS can be obtained from various sources and vendors. These two constituents strongly affect DC differentiation kinetics and efficiency. Hence, they should be carefully tested and Flt3L should be used at optimal concentration (see also section 1.2.2.5 Pitfalls).
- Cells can be harvested from days 7–8 onward until day 14. At earlier time points, the DC differentiation will not be advanced enough; at later points, too many cells will have died. In both cases, DC yield will be low. Importantly, pDC and cDC develop with different kinetics, with pDC being faster in their development. This should be taken into consideration when deciding at what time point to harvest DC.

In conclusion: the exact culture conditions i.e. cell number/well, Flt3L concentration, timing of medium changes, and time point of cell harvest, should be tested carefully and determined based on the purpose of the culture.

**Figure 13.**
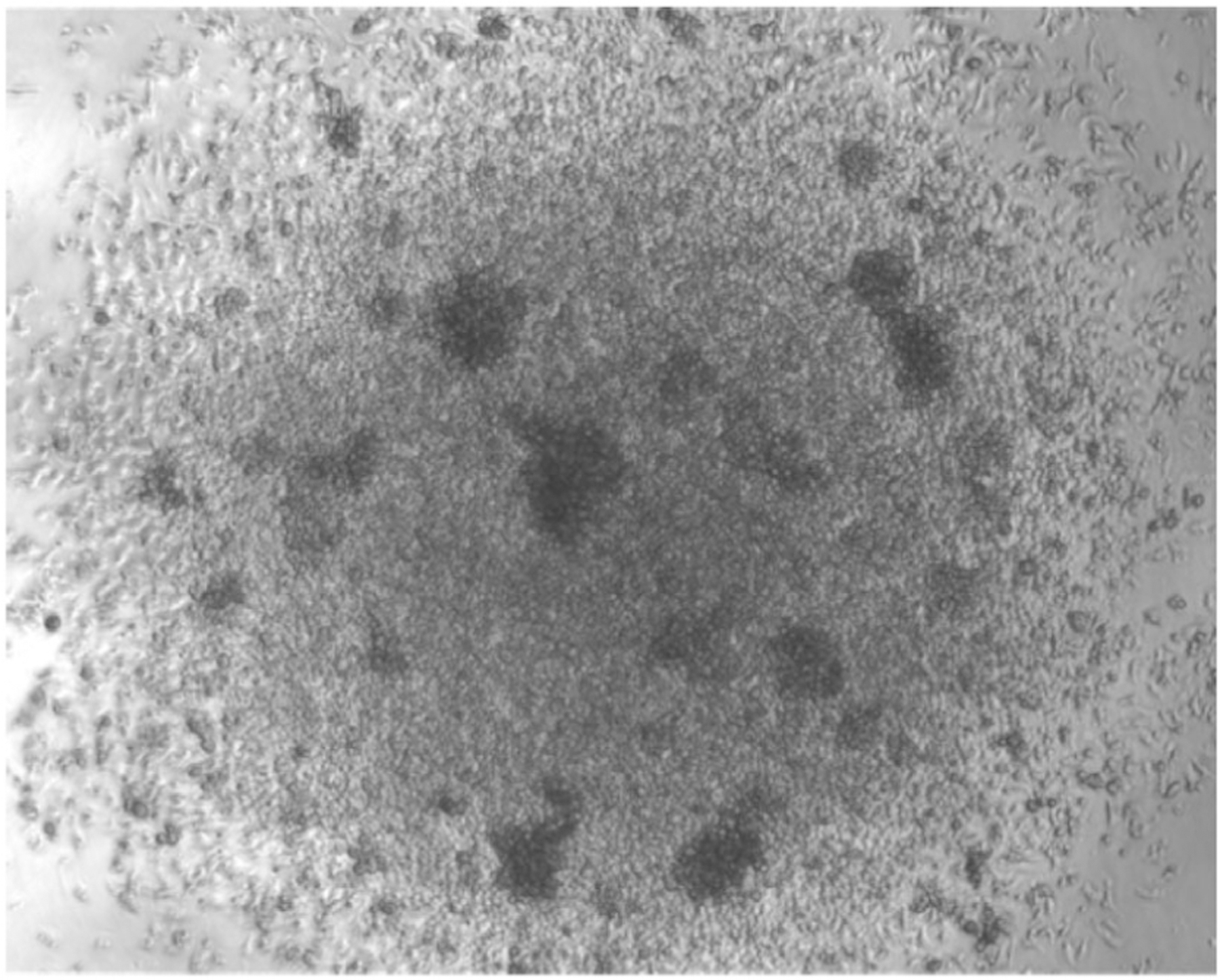
Phase contrast image of DC differentiated from BM with Flt3L. 0.3 × 10^6^ total BM cells were seeded in a well of a 96-well round bottom plate and cultured with Flt3L. The image is acquired after 7 days of culture.

Specific remarks:

- To step 5: Any size of dish can be used, such as 12- or 24-well plates, 6 cm or 10 cm dishes, T25 or T75 flasks. The number of cells seeded, and the volume should be adapted according to the area of the culture dish or flask, keeping in mind what is mentioned under general remarks. Also 96-well plates with flat or round bottom wells can be used (Fig. 13).
- To step 7 and 8: A partial medium change is performed to leave the differentiating cells undisturbed. To our experience, a full medium change leads to increased cell death and thus a lower yield of DC;
- To step 9: to quantify DC by flow cytometry, a precisely determined number of calibration particles, for example 10^4^, can be added to the well before harvesting the cells. This is in particular of interest when cells are cultured on small scale e.g. in 96-well plate format.

Expected yield:

During the first days of culture, cells that are not DC progenitors will die, which causes a significant drop in viable cell numbers. From day 5 onward, small clusters of proliferating cells develop, and cell density increases (Fig. 13). On average, 1 million DC can be obtained per 1 million BM cells seeded, and is an expected yield.

##### 1.2.2.4 Data analysis.

DC differentiation into cDC1, cDC2, and pDC subsets can be verified by flow cytometry. Cells can be stained in flow cytometry tubes or round bottom 96-well plates (e.g. #650180 Greiner Bio-one).

**Figure 14.**
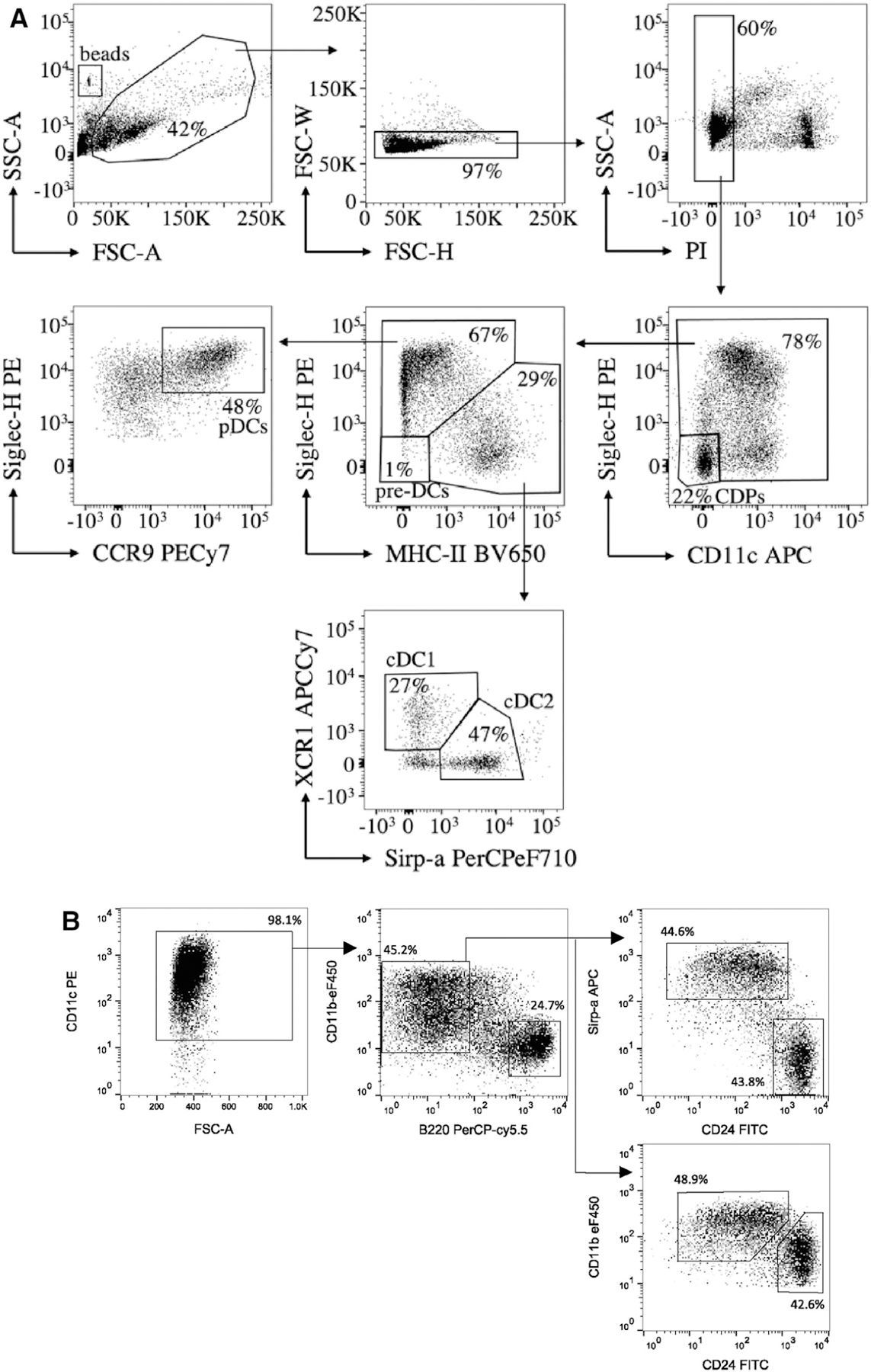
Two gating strategies for DC subsets generated from murine BM with Flt3L. Total BM cells were cultured for 7 (A) or 10 (B) days in a complete medium supplemented with Flt3L. (A) Before harvesting of the cells, calibration beads were added. Cells were then collected and processed for flow cytometry. Cells are first identified based on size and granularity by plotting the forward (FSC-A) versus side scatter (SSC-A). Doublets are then exclude based on the FSC-W and FSC-H, and live cells are identified as PI-negative. On single living cells, populations are determined as follows: CDP are CD11c^−^ Siglec-H^−^; pre-DC are CD11c^+^ MHC-II^−^ Siglec-H^−^; pDC are CD11c^int^ Siglec-H^+^ CCR9^+^; cDC are CD11c^hi^ MHC-II^+^ Siglec-H^low/−^ and can be further divided into cDC1 (XCR1^+^ Sirp-α^−^) and cDC2 (XCR1^−^ Sirp-α^+^). (B) Cells were collected and processed for flow cytometry. Living, single cells are selected (not shown) and analyzed for CD11c expression. CD11c^+^ cells are next split into pDC (B220^hi^ CD11b^−^) and cDC (B220^−^). cDC are further subdivided into cDC1 (Sirp-α^−^ CD11b^lo^ CD24^hi^) and cDC2 (Sirp-α^+^ CD11b^+^ CD24^lo^). Numbers near the gates represent percentage of parent gate.

1. Transfer 10^6^ cells into a flow cytometry tube (or 10^6^ cells into a 96-well plate);
2. Spin cells down for 5 min at 300 × *g*;
3. Remove supernatant and resuspend pellet in 1.5 ml staining buffer (or 200 μl in a 96-well plate);
4. Spin cells down for 5 min at 300 × *g*;
5. Remove supernatant and resuspend pellet in 50 μl staining buffer (or resuspend in the volume remaining in a 96-well plate);
6. Prepare the antibody mastermix;
7. Stain cells by adding 50 μl antibody mastermix per tube (or 30 μl in a 96-well plate);
8. Incubate for 30 min at 4°C;
9. Add 1.5 ml staining buffer and vortex (or 200 μl in a 96-well plate and resuspend by pipetting);
10. Spin cells down for 5 min at 300 × *g*; (repeat this step if using a 96-well plate);
11. Resuspend pellet in 250 μl staining buffer; (when using a 96-well plate: transfer cells to flow cytometry tubes or acquire directly from plate);
12. Right before acquisition, add 1 μl PI and mix well;
13. Acquire cells on flow cytometer;

Specific remarks:

- To step 1: less or more cells can be used for flow cytometry or FACS sorting. Volumes should be adapted accordingly;
- To step 6: Optimal antibody dilutions should be determined in advance. A typical antibody mastermix to determine DC subsets contains antibodies against CD11c, MHC-II, Siglec-H, CCR9, Sirp-α, XCR1 (Fig. 14A). Sirp-α and XCR1 are used to identify cDC2 and cDC1, respectively, but alternative surface markers can be used for this goal, such as CD24 for cDC1 and CD11b for cDC2 (Fig. 14B). CCR9 is used to determine pDC, alternatively CD45R (B220) can be used. Of note, next to pDC, cDC1, and cDC2, DC precursors can be identified at earlier time points during differentiation: common DC progenitors (CDP) and pre-DC as CD11c^−^Siglec-H^−^ and CD11c^+^ Siglec-H^−^ MHC-II^−^, respectively. Cells with pDC phenotype generated in this culture system express very low levels of MHC class II (lower than in spleen).
- To step 13: If beads are used to quantify DC by flow cytometry, FSC-A should be put in logarithmic scale. By acquiring FSC-W/FSC-H and SSC-W/SSC-H, doublets can be excluded. Other reagents can be used to exclude dead cells, such as 7-aad or DAPI. Acquiring 50,000 CD11c^+^ cells is sufficient. We typically use an LSRFortessa equipped with 4 lasers. Obviously, other flow cytometers can be used, but might require alternative fluorochrome combinations.

Expected yield:

At the optimal time point of DC differentiation, at least 90–95% of the cells should stain positive for CD11c (Fig. 14B).

##### 1.2.2.5 Pitfalls.

###### Problem: Low yield, poor DC differentiation

####### Potential solutions:

Efficient DC differentiation strongly depends on the utilization of an optimal FBS batch and Flt3L concentration. If DC numbers generated are low, or DC differentiation (CD11c^+^ cell production) is poor, consider the following:

- Different batches of FBS should be tested in a side-by-side comparison. A kinetic of differentiation should be included, as the optimal duration of differentiation is influenced by the FBS batch. Typically, maximal DC yields are obtained between days 7 and 14 of differentiation.
- Different sources of Flt3L can be used, for example, recombinant Flt3L (Peprotech), Ig stabilized Flt3L (BioXcell), or supernatant of cell lines stably transfected with Flt3L e.g. CHO-Flt3L-FLAG cells (Tracy Willson, The Walter and Eliza Hall Institute (WEHI), Melbourne, Victoria, Australia) or B16-F10 melanoma cells [9]. With each new batch of Flt3L, a titration should be performed to determine the optimal Flt3L concentration, as evaluated by DC numbers obtained. From our experience, the optimal Flt3L concentration might range between 30 and 400 ng/mL. A kinetic of DC differentiation should also be included for Flt3L testing. It was observed that longer culture may decrease the frequency of pDC while increasing the frequency of cDC ([62] and own observations).
- In function of the FBS or with sub-optimal Flt3L concentration, some macrophages may be present in the culture. However, they are adherent whereas DC are semi- or non-adherent. To enrich for DC by flow cytometry, cells expressing F4/80 (clone BM8) and/or auto-fluorescent cells should be excluded.

##### 1.2.2.6 Top tricks.

- Differentiation of DC in the presence of Flt3L in vitro is not complicated. It pays off, however, to spend time on carefully testing the culture conditions, most importantly FBS and Flt3L. For each new batch of FBS and Flt3L, we recommend determining the optimal Flt3L concentration, cell density during cell culture and kinetics of differentiation. Once this is done, DC generation with Flt3L will be highly reproducible.
- The percentage of cells with cDC1 phenotype may be low in FL-DC cultures. To increase the yield of cDC1, BM cells can be cultured on OP9 stromal cells expressing Delta-like 1 (OP9-DL1), which increases their CD8a expression and cross-presentation capacity. The addition of GM-CSF at a late stage during differentiation also improves cDC1 yield and upregulates their CD103 expression. This aspect is covered in section 1.2.3.
- The number of DC generated from BM can be substantially increased by including an amplification step first. BM cells cultured with a specific growth factor cocktail (SCF, Flt3L, IGF-1, and hyper-IL-6) generate large numbers of multipotent progenitors (MPP) [6, 7, 67]. In a second step, the MPP can then be differentiated with Flt3L into cDC1, cDC2, and pDC. Interestingly, this system recapitulates the sequel of MPP-CDP-cDC1/cDC2 development [67]. Additionally, MPP can be immortalized with a conditional HoxB8 estrogen receptor fusion gene (HoxB8-ER) yielding conditionally immortalized HoxB8 MPP for further studies, including CRISPR/Cas9 gene editing (see section 1.1.2).

##### 1.2.2.7 Summary Table.

Surface markers on mouse BM progenitors are summarized in Table 5.

#### 1.2.3 Generation of CD103^+^ conventional type 1 DC (cDC1) from mouse BM

##### 1.2.3.1 Introduction.

Dendritic cells (DC) are professional antigen-presenting cells originally classified as classical or conventional DC (cDC), plasmacytoid DC (pDC), monocyte-derived DC (moDC), and Langerhans cells (LC) [68]. This basic classification, mainly based on phenotype and ontogeny, is subjected to continuous evolution as phenotypic heterogeneity exists within each family. In mice, two different lineages have been identified within the cDC subset: cDC1 and cDC2. The cDC1 lineage includes CD8α^+^ DC resident to lymphoid tissues and its migratory CD103^+^ DC counterpart in non-lymphoid tissues. Most cDC1 across lymphoid- and non-lymphoid tissues express high levels of CD24 and Clec9A but lack CD11b and signal regulatory protein α (SIRP-α) expression. In contrast, the cDC2 lineage is characterized by high levels of SIRP-α and CD11b expression, and the absence of typical cDC1 markers [69].

Due to their low abundance and difficulty to retrieve from peripheral tissues, the study of DC properties and functions has traditionally relied on in vitro cultures of BM progenitors in the presence of distinct growth factors. Thus, a very common strategy for DC generation consists of the use of GM-CSF to differentiate BM cells into a type of moDC [33].

In addition, fms-like tyrosine kinase 3 ligand (FLT3L), a key cytokine to DC ontogeny *in vivo*, has been exploited to induce differentiation of BM precursors into various DC subsets: CD24^+^ cDC1, SIRP-α^+^ cDC2, and pDC [26]. Importantly, these DC subsets were proven to be comparable to splenic counterparts and thereby represent a valuable tool to study spleen DC subset ontogeny and function. However, although FLT3L-DC constitute a better approximation to *bona fide* DC than GM-DC, their practical use is still limited by the high cellular heterogeneity of DC subsets in FLT3L cultures. Moreover, the few cDC1 found within FLT3L-DC cultures express little CD103, a hallmark of migratory cDC1 found in non-lymphoid tissues in vivo.

Considering the hindrance to generate CD103^+^ cDC1 with available methods and their relevance for antigen cross-presentation in the context of antiviral protection, vaccination, and tolerance induction [70], our laboratory developed a novel method for efficient CD103^+^ cDC1 generation [71]. As a result, this culture system (termed iCD103-DC) yields several millions CD103^+^ cDC1 in contrast to the very low numbers that can be obtained from FLT3L-DC cultures or murine tissues. Importantly, iCD103-DC cultures are more homogenous since they produce predominantly CD103^+^ cDC1 with little differentiation of SIRP-α^+^ cDC2 or pDC (Fig. 16 below). Furthermore, iCD103-DC are functionally and phenotypically similar to the CD103^+^ DC found in vivo [70].

Hence, the iCD103-DC culture is an advantageous resource in the process of understanding CD103^+^ DC/cDC1 biology and therapeutic potential.

##### 1.2.3.2 Materials.

###### 1.2.3.2.1 Reagents.

A detailed list of necessary reagents for iCD103-DC generation and antibodies are provided in Tables 6 and 7, respectively.

###### 1.2.3.2.2 Equipment.

Necessary equipment is listed in Table 8.

##### 1.2.3.3 Step-by-step sample preparation.

###### BM cell isolation and differentiation

Day 0:

1. Isolate BM cells from murine femurs and tibias.
2. Seed 15×10^6^ BM cells into a 10 cm-dish in a total volume of 10 mL of complete medium supplemented with 11.5% FLT3L (50 ng/mL) and 1.3% GM-CSF (1.5 ng/mL) supernatants.
  ∗ Note: Henceforth, complete medium consists of RPMI supplemented with 2-mercaptoethanol (50 μM), penicillin (100 u/mL)/streptomycin (0.1 mg/mL) and FCS (10% v/v).
3. Place the petri dish in a 37°C and humified 5% CO2 incubator.

Day 5 or 6:

- Add 5 mL of pre-warmed complete medium without any growth factor.

Day 9:

- Harvest the cells by gently pipetting with a serological pipet and transfer them into a 50 mL tube.
- Homogenize the cell suspension gently and count the cells. At least 10 × 10^6^ cells/dish are expected on day 9 if the first part of the culture is successful.
- Centrifuge at 400 × *g* at 4°C for 7 min to pellet the cells. Discard the supernatant and resuspend the pellet in a suitable volume of complete medium.
- Seed 3 × 10^6^ cells into a 10 cm-petri dish in a total volume of 10 mL of complete medium supplemented with 11.5% FLT3L (50 ng/mL) and 1.3% GM-CSF (1.5 ng/mL) supernatants

**Figure 15.**
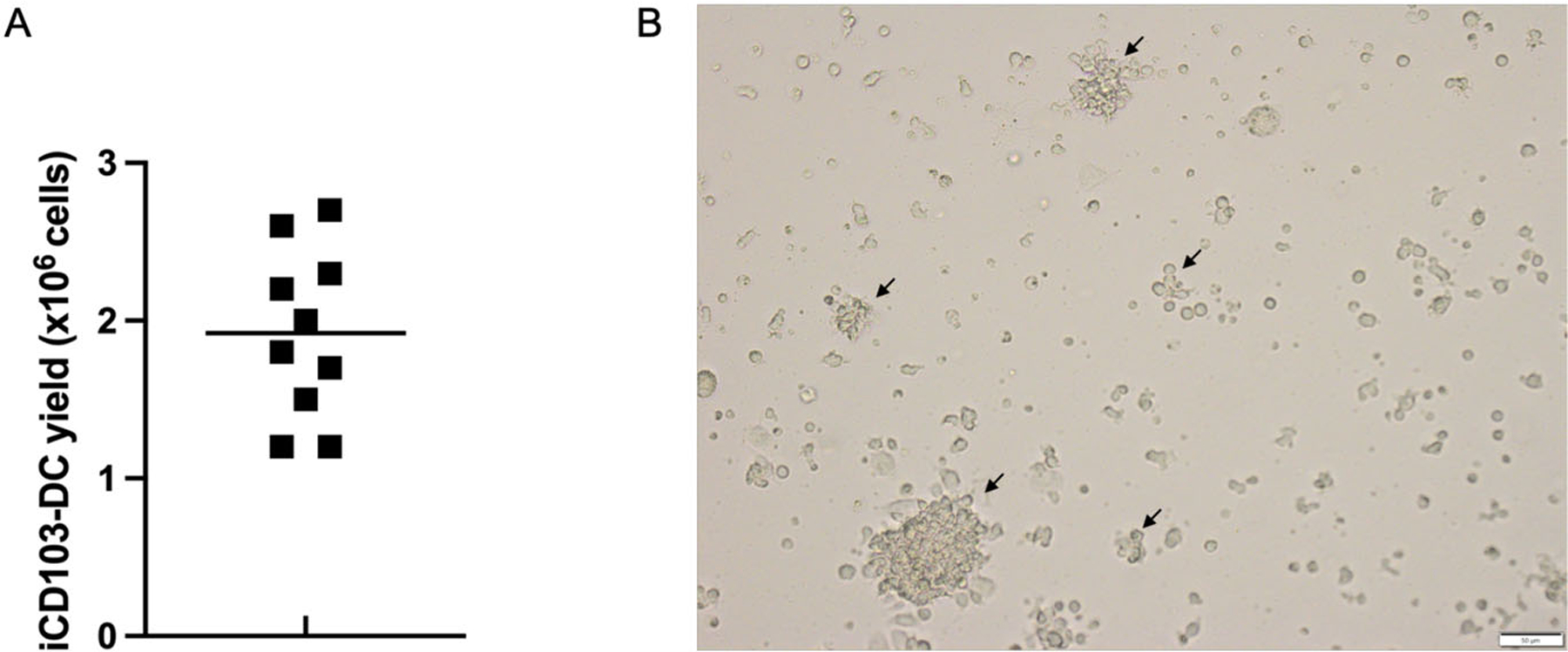
Yield and microscopic appearance of iCD103-DC cultures. **(A)** Scatter plot showing the absolute yield of live CD11c^+^B220^−^Clec9A^+^CD103^hi^ cells obtained per 1×10^6^ input BM cells. iCD103-DC were harvested on d16 and the cell yield evaluation was performed by using Trypan Blue stain. Results represent 10 independent experiments, each involving 1 culture from individual donor mice. (**B)** Typical bright-field micrograph of iCD103-DC cultures. Cells were harvested on day 16. Black arrows indicate the presence of floating cell aggregates. Scale bars represent 50 μm. Image was acquired on an inverted microscope (Olympus).

Day 16:

- Harvest the cells by gently pipetting with a serological pipet and transfer them into a 50 mL tube. 3–6×10^6^ DC/dish and 1–3×10*^6^ DC/1×10*^6^ input BM cells are expected on day 16 (d16) (Fig. 15A).
- Reserve an aliquot of cell suspension (0.5–1 × 10^6^) to assess by flow cytometry that the differentiation occurred efficiently.

###### Assessment of cell culture composition by flow cytometry

**Figure 16.**
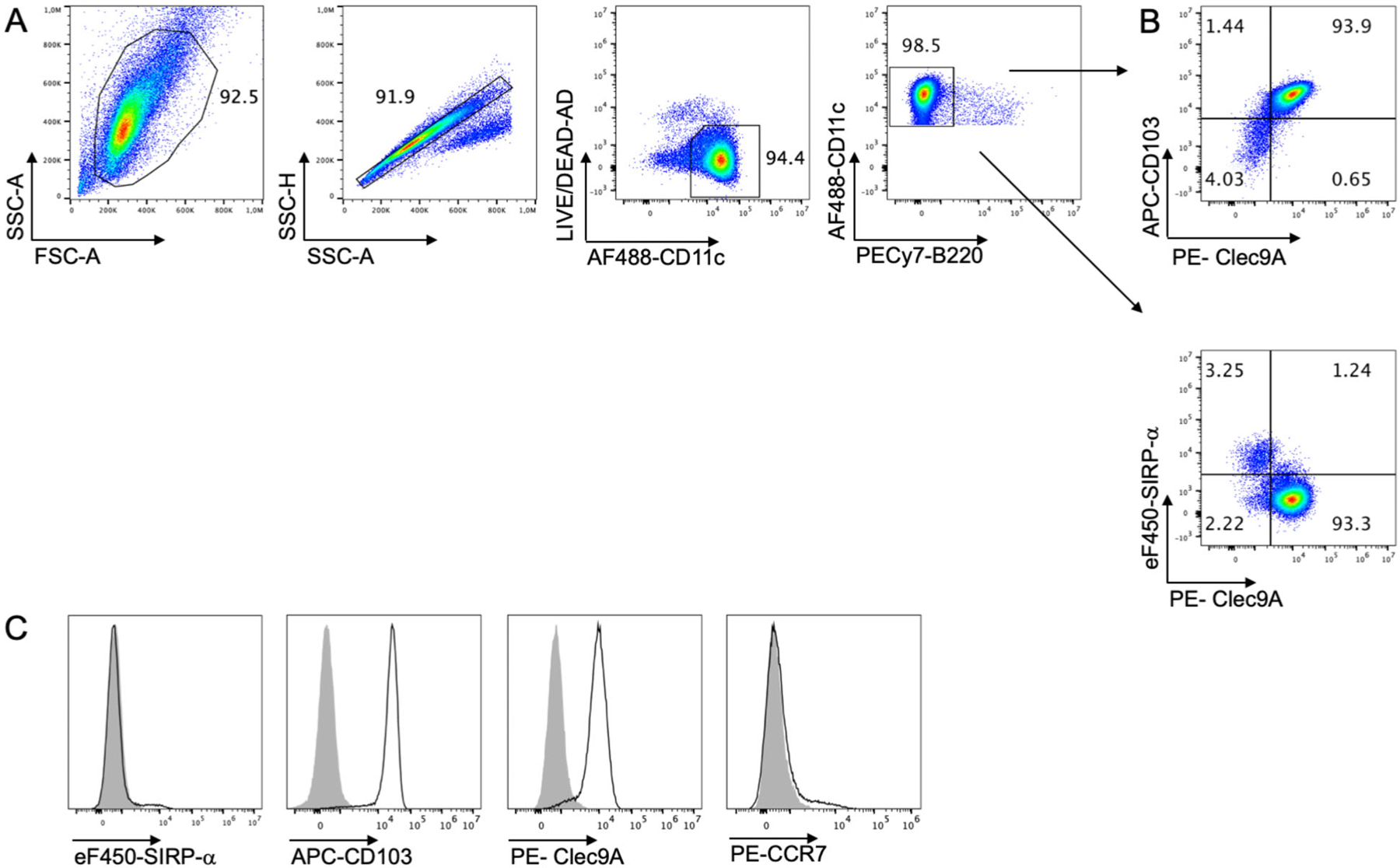
Gating strategy for identification of iCD103-DC. **(A)** Representative flow cytometry (FC) analysis of the gating strategy applied for the identification of iCD103-DC obtained on day 16. BM-DC were first gated based on forward and side scatter area (FSC-A and SSC-A) and doublets and debris were excluded by gating on area vs the height of SSC (SSC-A and SSC-H). Dead cells were excluded using fixable live-dead aqua dead cell dye. (**B)** Representative dot plots showing frequencies of Clec9A^+^CD103^+^ and Clec9A^+^SIRP-α^−^ cells. Gates were performed on live CD11c^+^B220^−^ iCD103-DC as shown in (A). (**C)** Representative histograms were obtained by FC of live CD11c^+^B220^−^ cells. Histograms represent the expression levels of SIRP-α, CD103, Clec9A, and CCR7 on d16 (black lines) and the respective control staining (grey overlay). In this example, 2 different staining panels were used.

- Transfer a proper volume of cell suspension to polypropylene flow cytometry tubes and spin down at 400 × *g* at 4°C for 7 min. Discard supernatant by inversion. Wash twice with DPBS and stain the cells with fixable live/dead exclusion dye in DPBS 20 min at 4°C protected from light.
- Wash the cells with washing buffer and add Fc blocking reagent to minimize non-specific antibody interactions with Fc receptors. Incubate for 10 min at 4°C in the dark.
  ∗ Note: flow cytometry washing buffer is made of DPBS supplemented with 0.2% w/v BSA, 0.02% w/v sodium azide, and 2 mM ethylenediaminetetraacetic acid (EDTA).
- Add the surface antibody mix (see table 7) and incubate for 20 min at 4°C in the dark.
- Wash the cells with washing buffer and centrifuge at 400 × *g* at 4°C for 5 min. Discard supernatant.
- Resuspend stained cells in a proper amount of washing buffer. Acquire samples straightaway in flow cytometer or fix with 2% paraformaldehyde (PFA) for 20 min. The typical phenotype of iCD103-DC is illustrated in Fig. 16.

##### 1.2.3.4 Data analysis.

This method has the benefit of simplicity in addition to being selective and efficient, thus eliminating the need for further purification steps for certain applications. From 2 femurs and 2 tibias, 60–100×10^6^ CD103^+^ iCD103-DC can be easily obtained compared with 0.5×10^6^ CD103^+^ DC that can be isolated from tissues by enzymatic digestion and cell sorting [71]. The absolute iCD103^+^ cDC yield of this method at d16 is shown in Fig. 15A.

The presence of floating cell aggregates can be monitored by using an inverted light microscopy on day 16 and indicates a suitable iCD103-DC differentiation as shown in Fig. 15B.

The cell culture composition of differentiated iCD103-DC can be analyzed by flow cytometry at the end of the culture. After 15–16 days of differentiation, 90% of the cells are CD11c^+^B220^−^ cDC (Fig. 16A). Among the cDC, more than 90% of the cells are Clec9A^+^CD103^(hi)^SIRP-α^−^ DC (Fig. 16B and C), resembling to a high degree the phenotype displayed by *ex vivo* isolated CD103^+^ cDC1. CCR7 expression was low on unstimulated DC as expected [71]. Importantly, Clec9A^+^CD103^(hi)^SIRP-α^−^ iCD103-DC completely rely on Batf3 for development in culture [71], confirming their CD8α-like cDC phenotype.

**Figure 17.**
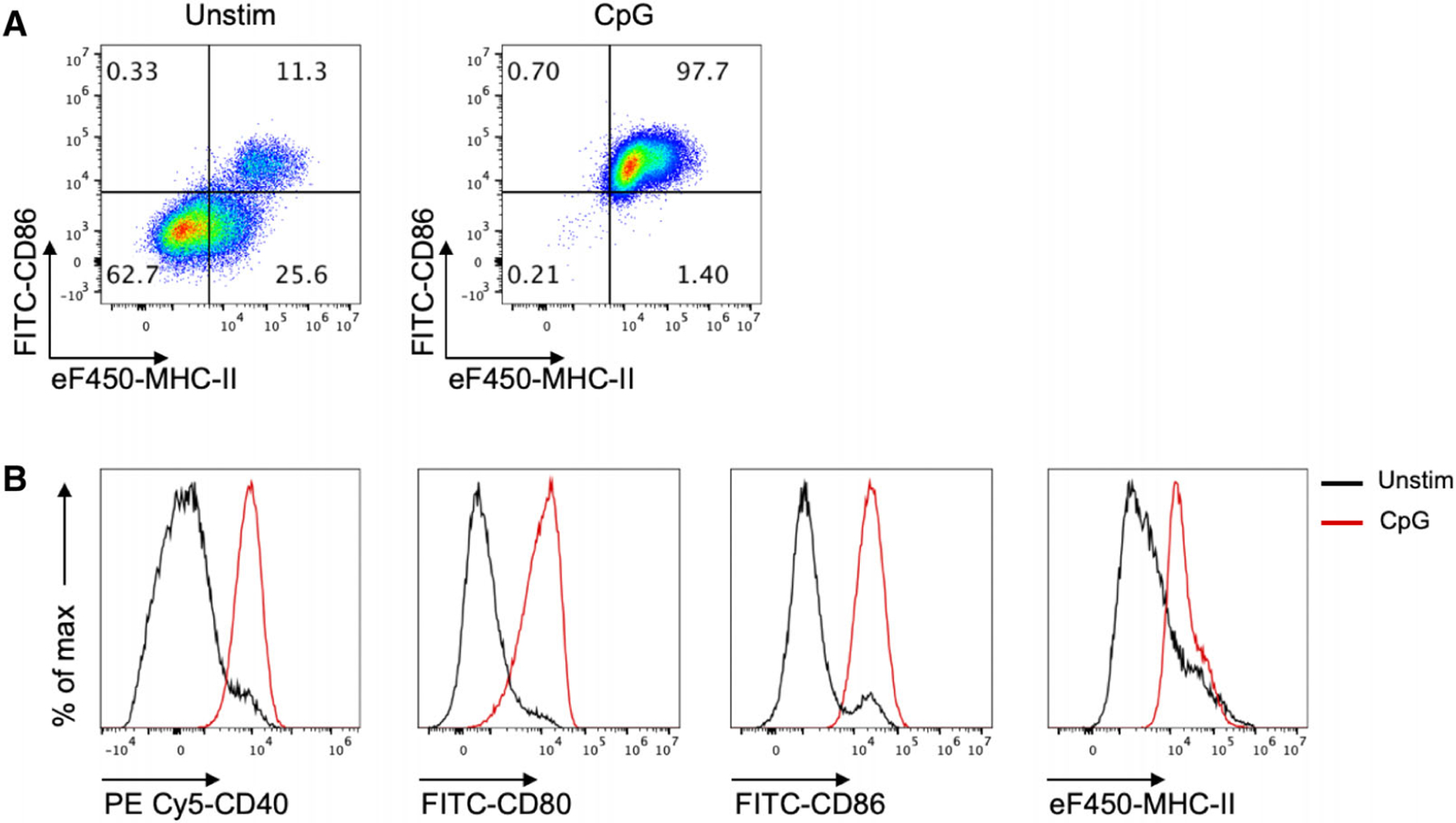
Maturation status of differentiated iCD103-DC. **(A)** iCD103-DC were harvested on d16 and stimulated in a 96-well F-bottom cell culture plate overnight in the presence or absence of CpG-1826 ODN (1μM). Representative flow cytometry dot plots showing the frequencies of MHC-II^+^CD86^+^ cells among live CD11c^+^B220^−^CD103^+^Clec9a^+^ cells. (**B).** The expression of CD40, CD80, CD86, and MHC-II were analyzed by flow cytometry among live CD11c^+^B220^−^CD103^+^Clec9a^+^ cells in the presence (solid red line) or absence (solid black line) of overnight CpG-1826 ODN stimulation. In this example, 2 different staining panels were used.

The maturation status of iCD103-DC was analyzed by flow cytometry in the presence or absence of overnight CpG stimulation (1 μM). As shown in Fig. 17A, a low proportion of iCD103-DC express MHC-II and CD86 molecules under unstimulated conditions. Additionally, they exhibit low expression levels of common co-stimulatory molecules such as CD40 and CD80 (Fig. 17B). Upon CpG activation, iCD103-DC highly express all the maturation markers tested (Fig. 17B), and they also upregulate CCR7 [71]. Moreover, Mayer et al. [71] provided conclusive evidence that activated iCD103-DC are able to initiate specific T cell responses in a similar fashion to their in vivo equivalents.

A summary of typical surface markers for the analysis of iCD103-DC by flow cytometry is included in Table 9 (below).

##### 1.2.3.5 Pitfalls.

The BM differentiation was set up by supplementing the culture medium with culture supernatant containing murine FLT3L-flag and culture supernatant containing murine GM-CSF, both produced in-house as described [33, 63]. The optimal concentration was determined for each batch by ELISA.

As different batches of FLT3L/GM-CSF supernatants can vary substantially, it is recommended that each batch is first tested by ELISA. Afterward, different supernatant concentrations should also be titrated to identify the conditions optimal for iCD103-DC differentiation.

In our own experience, the concentration and quality of growth factors largely affect the proliferation and survival of DC or their precursors. Commercially available recombinant growth factors could also be used yet their respective concentrations must be tested beforehand. We successfully used 200 ng/ml rmFLT3L (Cat. 14–8001, eBioscience) and 2–5 ng/ml rmGM-CSF (Cat. 315–03, Peprotech), yet the yield tends to be lower than with in-house produced supernatants [71]. If GM-CSF and FLT3L are to be produced in-house as we recommend, testing for lipopolysacharide (LPS) presence or mycoplasma contamination should be conducted in the batch stocks. Negative results in these tests are required to assure the generation of immature iCD103-DC.

Even though the purity obtained with this method is suitable, additional methods could be adopted as needed. For instance, live cells enrichment alongside with dead cells/debris removal can be accomplished by using OptiPrep density gradient on days 15 and 16 (end point).

##### 1.2.3.6 Top tricks.

A careful monitoring of the cell culture under a standard light microscope gives an impression of the quality, e.g. viability across the culture protocol and floating cluster formation at day 16. Special care must be taken as well to maintain the sterility in all the steps.

When performing BM isolation, progenitor cell numbers may be decreased if one of the bones is broken or the soft tissue is inadequately removed.

The number of BM cells was set up to obtain maximal numbers of the CD103^+^ cDC1 fraction. Although it might be possible to start the BM differentiation with less than 15×10^6^ cells, it is expected that the yield can drop.

The culture medium addition in the first differentiation step (days 5–6) is critical to minimize cell death since BM precursors proliferate extensively at this stage. If this is not performed, the survival rate and a number of BM cells will be reduced by day 9. The culture medium should be observed for acidity, as a color change to orange/yellow indicates that media addition is necessary. This step appears to be less critical when recombinant cytokines are being used.

During the second differentiation step, high proliferation is not expected while cell differentiation should become dominant. iCD103-DC tends to cluster into floating cell aggregates (Fig. 15B), therefore, the occurrence of it together with the presence of few single cells between days 15 and 16 indicate accurate differentiation. On the contrary, few DC clusters and many single cells together with a high proliferation rate are indicative of poor differentiation. In the latter case, the purity will be negatively affected.

Normally, differentiated iCD103-DC are uniformly immature as shown in Fig. 17 (i.e., MHC-II and CD86 low expression). However, iCD103-DC must be handled carefully to avoid undesirable pre-activation, since the harvesting itself can cause 20–50% of DC expressing high levels of MHC-II and costimulatory molecules. Therefore, leaving the cells in their 10 cm-Petri dishes might avoid their activation. The addition of FLT3L and GM-CSF at the same concentration used for the BM differentiation is recommended when stimulation overnight is required (e.g., CpG or pathogens), as it was shown to increase DC survival.

##### 1.2.3.7 Summary table.

The panel used for the characterization of differentiated iCD103-DC is detailed in Table 9.

#### 1.2.4 Purification of murine pDC from BM-Flt3L cultures by Fluorescence Activated Cell Sorting (FACS)

##### 1.2.4.1 Introduction.

**Figure 18.**
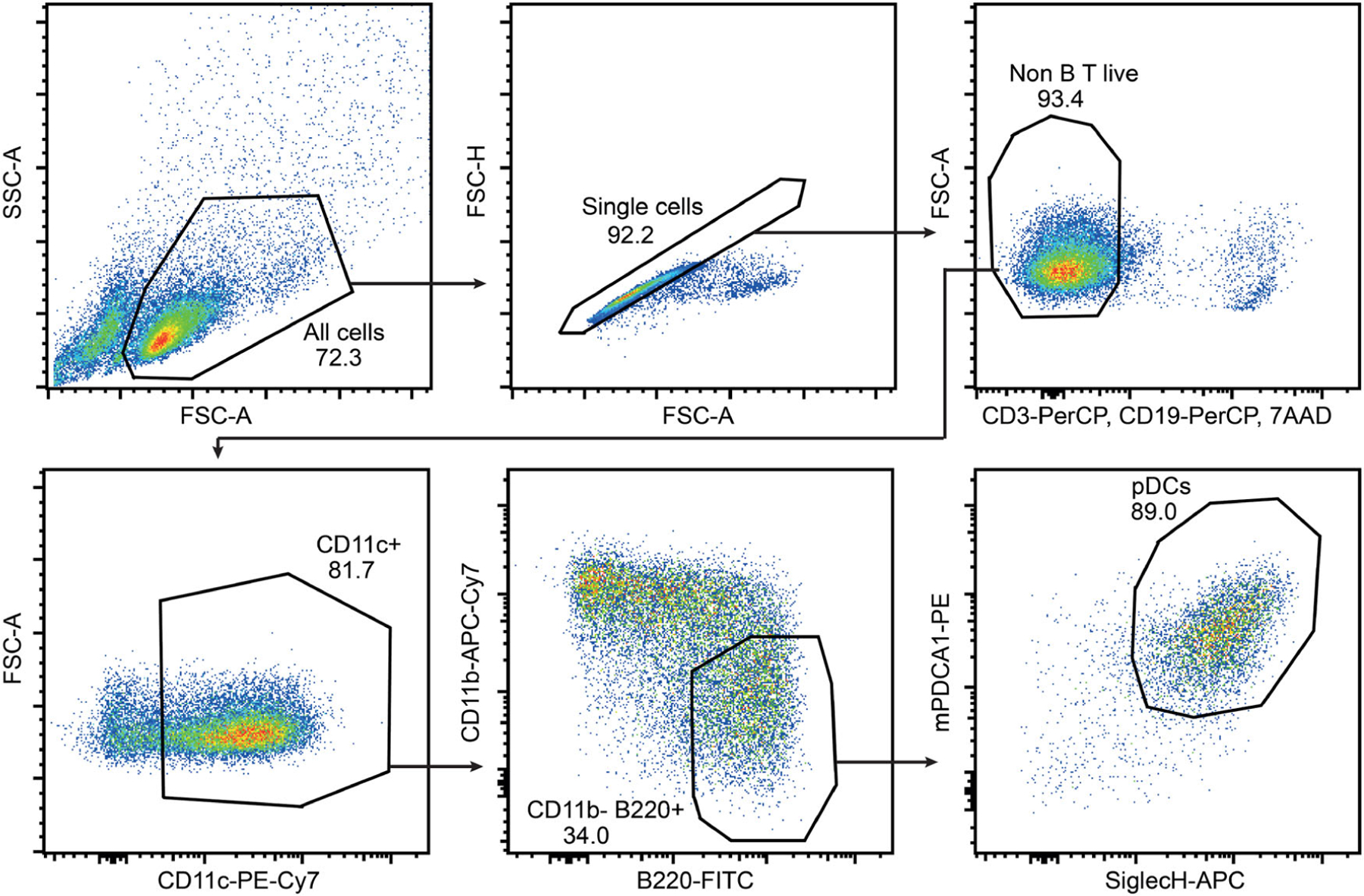
The gating strategy for sorting pDC from BM-Flt3L cultures. The assay was performed following the protocol described in the text. The data was recoded using BD FACS Aria^™^ III. The FACS blots shown above are generated using FlowJo 10. Follow the gating from left to right. Cells were gated as cell-sized events according to FSC-A versus SSC-A profile, followed by single-cell events based on their FSC-A and FSC-H features. From the single events, the cells negative for the expression of B (CD19), T (CD3) cell surface markers and remained unstained for 7AAD (dead cell marker) were gated as live non-B/T cells. From this live non-B/T cell population CD11c^+^ cells were discriminated and analyzed for the expression of CD11b versus B220 (CD45R). pDC were defined as CD11b^−^B220^+^ cells which further express SiglecH and mPDCA1 (CD317, Bst2).

In this protocol, we provide a flow cytometry-based workflow for the detection and purification of BM-derived pDC from Flt3L cultures (Fig. 18). We used BD FACSAria^™^ III to optimize this protocol for purifying pDC. In this protocol, we stained Flt3L cultured cells specifically for pDC. However, total cDC can also be sorted in parallel using the same protocol with slight adaptation of the cDC gating strategy. In order to sort cDC subpopulations like cDC1 and cDC2 we recommend staining of additional subpopulation specific cell surface markers. For the general principles of flow cytometry and cell sorting as well as the technical information about different components of a flow cytometer, follow the guidelines for the use of flow cytometry and cell sorting in immunological studies [72]. Check the lasers and filter configuration of your cell sorter before planning your sort experiment. The protocol described here is optimized using a customized BD FACSAria^™^ III, equipped with the blue, yellow-green, and red lasers. You may need to adopt the protocol according to your cell sorter, laser, and filter configuration.

The resting pDC are approximately 10 μM in diameter. When activated, pDC increase their size and become around 15 μM large cells [73]. Therefore, the 85 μM nozzle (following basic principle, nozzle size *>* 3× cell size) is recommended to sort pDC. pDC FACS purified using this protocol are suitable for various downstream applications such as DNA, RNA, and protein characterization, including gene expression profiling and chromatin landscape analysis etc. Purified pDC are viable and appropriate for imaging, re-culturing, transplantation, IFN production, and/or evaluation of other functions such as migration, antigen presentation, etc. The procedure covers various preparatory steps including harvesting, cell surface staining, sorting, and re-culturing for activation of pDC.

##### 1.2.4.2 Materials.

###### 1.2.4.2.1 Reagents.

Following materials are required to sort pDC from BM-Flt3L cultures.

Medium and Buffers

- RPMI 1640 (PAN Biotech, cat.no. P04–17525) cell culture medium
- l-Glutamine (Gibco, cat.no. 25030–081)
- 2-Mercaptoethanol (Gibco, cat. no. 31350–010)
- PBS (Gibco, cat.no. 14190–094)
- FCS (Sigma-Aldrich, cat. no. F7524)
- UltraPure^™^ 0.5 M EDTA, pH 8.0 (Invitrogen, cat. No. 15575–020)
- Trypan blue (Gibco, cat.no. 15250–061) and hemocytometer (Marienfeld, cat.no. 0640010) for cell counting
- FACS buffer (PBS containing 2%FCS and 2 mM EDTA)
- pDC medium (RPMI 1640 supplemented with 10%FCS, 0.2 mM 2-Mercaptoethanol, 2 mM l-Glutamine and approximately 100 ng/ml Flt3L by adding 5% CHO-Flt3L cells conditioned medium)

Antibodies for staining pDC

- Anti-mouse CD16/CD32, clone#93, eBioscience, cat.no. 14–0161-82
- FITC rat anti-mouse B220 (CD45R), clone#RA3–6B2, BD Bioscience, cat.no. 553088
- PE rat anti- mPDCA-1 (CD317, Bst2), clone# ebio927, eBio-science, cat.no. 12–3172–82
- Percep-Cy5.5 hamster anti-mouse CD3e, clone# 145–2C11, BD Bioscience, cat.no. 551163
- Percep-Cy5.5 rat anti-mouse CD19, clone# ID3, BD Bioscience, cat.no. 551001
- PE-Cy7 hamster anti-mouse CD11c, clone# N418, BioLegend, cat.no. 117318
- APC- rat anti mouse SiglecH, clone# 551, BioLegend, cat.no. 129612
- APC-Cy7 rat anti-mouse CD11b clone# M1/70, BD Bioscience, cat.no. 557657

###### 1.2.4.2.2 Consumables.

- Compensation beads (BD, cat no. 552845)
- Ice
- 5 ml, 10 ml, and 25 ml serological nonpyrogenic pipettes (Costar, cat.no. 4487, 4488, and 4489)
- 50 ml conical tubes (Sarstedt, cat. no. 62.547.254) for collection and staining cells
- 15 ml conical tubes (Falcon, cat. no. 352097) for collection of sorted cells
- 5 ml polystyrene round-bottom FACS tubes (Falcon, cat. no. 352054) with cap for staining single color controls, collection tubes for filtering of cells or collection tubes for sorted cells
- Sterile cell scraper (Greiner Bio-One, cat. no. 541070) for detaching semi-adherent cells
- 96-well flat-bottom plates, tissue culture not treated (Falcon, cat. no. 351172) for culturing cells after sorting
- 24-well flat-bottom plates, tissue culture not treated (Costar, cat. no. 3738) for culturing cells after sorting
- Cell strainer, 50 μm (CellTrics® filters, Sysmex, cat. no. 04–004–2327) for filtering cells before acquisition

###### 1.2.4.2.3 Equipment.

- Laminar flow hood (CleanAir)
- Cell centrifuge (Andreas Hettich GmbH & Co. KG)
- FACS sorter (BD FACS Aria III^™^, BD Biosciences)
- Phase-contrast microscope (Zeiss)
- Fridge (Liebherr) for culturing cells at 4°C
- Humidified CO_2_ cell incubator (Hera cell, Thermoscientific) for culturing cells at 37°C

##### 1.2.4.3 Step-by-step sample preparation and cell sorting.

###### Harvesting BM-Flt3L cultures

Culture mouse BM cells in Flt3L containing medium according to the BM-derived DC generation protocol described in the Section 1.2.2 of this chapter. After 7 days of culturing in Flt3L containing medium cells are ready for harvesting and further activation procedures. To avoid any kind of undesired cell activation and be able to further culture the purified pDC follow and maintain aseptic practices throughout the procedure.

1. Examine the Flt3L culture plates under a phase contrast microscope to exclude any kind of bacterial or fungal contamination. After 7 days of BM-culturing in Flt3L containing medium one should expect mostly non-adherent, round, and bright cells. There are also some semi-adherent flat cells on the bottom of the plate.
2. Put the cell culture plates at 4°C in a fridge for 30 min. Cooling cells helps in detaching the semi-adherent cells from plastic surface.
3. Detach the adherent cells with the help of a sterile cell scraper.
4. Resuspend the cells and transfer the cell suspension to 50 ml conical tubes. Cells from up to three plates (around 50 × 10^6^ cells) can be collected and stained in one 50 ml conical tube.
5. Wash the plates twice with 5 ml PBS to collect the rest of the cells in the same tube. Take an aliquot of the cell suspension for counting. Keep cells at 4°C until further use.
6. Count cells and document the cell number.

###### Cell surface staining for sorting pDC

Take all or one of the tubes of the cells from Step 6. Use around 10–60 × 10^6^ cells for staining. Depending upon the requirement, start with a sufficient number of cells to obtain a required number of pDC after sorting. If only few purified pDC are required for the assay, one can downscale the staining volume. We usually stain 10–50 × 10^6^ BM-Flt3L cells to achieve sufficient pDC for a good downstream application. The expected yield of purified pDC varies between 10% and 15% of the input BM-Flt3L cells. We suggest staining 10 times more BM-Flt3L input cells than the required number of purified pDC for the experiment.

- Centrifuge cells at 1200 rpm (300–500 × *g*) at 4 °C for 5 min in a cell centrifuge.
- Fc-block: Prepare the blocking antibody solution by diluting anti-mouse CD16/32 (1:50) in FACS buffer.
- After centrifugation in step 7, aspirate the supernatant.
- Resuspend the cell pellet in 100 μl Fc-block solution to block Fc receptor on the mononuclear cells.
- Incubate the cells at 4°C for 10 min.
- Prepare the staining mixture by diluting following fluorescent-conjugated staining antibodies: FITC-anti B220 (1:50), PE-anti mPDCA-1 (1:50), PerCP-anti CD3 (1:100), PerCP-anti CD19 (1:100), PE-Cy7-anti CD11c (1:100), APC-anti SiglecH (1:50), APC-Cy7-anti CD11b (1:100). For sorting cDC subpopulations we recommend to stain cells additionally for XCR1 and CD172a (Sirp-a). We recommend titrating antibodies dilutions for optimal staining.
- After completion of 10 min of incubation (step 11), add 200 μl of the staining mixture (from step 12) to the cells without any washing step.
- Resuspend the cells and incubate at 4°C for 30 min in the dark.
- Wash the cells by adding 25 ml FACS buffer in each tube.
- Centrifuge cells at 1200 rpm at 4°C for 5 min in a cell centrifuge.
- Aspirate the supernatant, resuspend the cells pallet in 500 μl FACS-buffer, and keep on ice until further use.
- Add 10 μl 7AAD, a live/dead DNA staining marker in the cell suspension 10 min before analysis.

###### Preparing compensation controls

- For each compensation control, label a FACS tube with the respective fluorescence-dye name.
- Add 100 μl of FACS buffer in each FACS tube.
- Resuspend the positive (anti-Rat and anti-Hamster Ig, κ) and negative (Negative Control) compensation beads thoroughly in the stock tubes before use. For each compensation control, add one drop of positive beads and one drop of negative beads to 100 μl of FACS buffer in a FACS tube.
- For the negative control, do not add any antibody. For the positive single antibody staining FACS controls, add 1 μl of respective fluoresce-conjugated antibody in the pre-labeled tube for each control.
- Incubate the FACS tubes for 10 min in the dark. Add an additional 100 μl FACS buffer in each tube and keep at 4°C in dark until further use.

###### Acquisition and cell sorting

- Follow the standardized instructions to operate the sorting instrument and the software as provided by the sort facility/supplier/producer. Briefly, we recommend stabilizing the flow stream at least 30 min before start of the sorting process. We use a 85 μm nozzle at 45 psi sheath pressure to sort pDC. Perform instrument quality control (baseline and performance check) and optimize drop delay as recommended by the supplier before starting the sort process. Do not forget to direct the side streams into the middle of the collection tube.
- Filter the cell samples through 50 μm sterile disposable cell strainer before starting acquisition. Adjust target cell concentration around 10^6^/ml, and sort pDC at possible lowest sample flow rate to avoid stress on the cells. The event rate 10,000/second may yield an efficiency around 70% for sorting pDC from Flt3L cultures when using BD FACSAria^™^ III. To keep cells cold, adjust the temperature of sample chamber as well as the collection chamber to 4°C. Continuously stir the sample tube at 200 rpm to maintain the uniform cell suspension and avoid cell sedimentation. We use 4way purity precision to sort pure pDC form Flt3L cultures.
- Coat the collection tubes (15 ml conical tubes or 5 ml FACS tubes) by filling the tubes with cell culture grade FCS. Warm up collection tubes to 37°C and keep them on ice until use. Remove the FCS just before use. Leave 1 ml or 500 μl FCS in the 15 ml conical tube or 5 ml FACS collection tube respectively.
- Create an experiment with a sample tube. Load sample tube, start acquisition and adjust PMT voltages.
- Create compensation controls. Run the compensation controls. Calculate the compensation matrix (automatic process, using the operating software defined compensation parameters), link and save to sample tubes.
- Acquire a sample and record 10000 events.
- If needed fine tune compensation manually.
- Determine the gating strategy for pDC as in Fig. 18 (one can expect 30–45% pDC in the BM-Flt3L cultures). For sorting total cDC and DC subpopulations, we recommend the following gating strategies.
  **pDC** = Live, single, CD3^−^, CD19^−^, CD11c^+^, CD11b^−^, B220^+^, SiglecH^+^ and mPDCA-1^+^
  **Total cDC** = Live, single, CD3^−^, CD19^−^, CD11c^+^, CD11b^+^, B220^−^ and SiglecH^−^
  **cCD1** = Live, single, CD3^−^, CD19^−^, CD11c^+^, CD11b^+^, B220^−^, SiglecH^−^, XCR1^+^ and CD172a-
  **cCD2** = Live, single, CD3^−^, CD19^−^, CD11c^+^, CD11b^+^, B220^−^, SiglecH^−^, XCR1^−^ and CD172a^+^
- Load the collection tubes into the collection chamber.
- Open sort layout, insert the pDC population (and/or any other DC population) into sort position, choose sort precision, apply sort tubes and start sorting DC.
- Once required number of purified pDC is achieved or collection tube is filled, stop sorting, remove the collection tube, cap and keep the collection tube on ice until all sample are sorted.

###### Washing and re-culturing pDC

- Fill the collection tubes with complete medium (without Flt3L), cap, and centrifuge the collection tubes in a cell centrifuge at 1200 rpm (300g) for 5 min at 4°C.
- Aspirate the cell-free supernatant, leaving around 200 μl without disturbing the cell pellet.
- Depending upon the downstream application, wash the cells once with 5 ml complete medium (without Flt3L) if the cells will be re-cultured for any bioassay. Alternatively, cells should be washed with ice-cold PBS for direct DNA, RNA, or protein purification.
- Centrifuge the collection tubes as in step 35.
- Aspirate the supernatant, leaving around 100 μl with the cell pellet untouched. Store the sorted pDC on ice until use.
- For re-culturing cells for a bioassay e.g. type I IFN production after TLR9 activation, re-suspend the cells in complete pDC-medium containing Flt3L as 1–2×10^6^ cells/ml.
- Culture pDC in a 96 well plate, 200 μl/well (approximately 1 × 10^5^–2 × 10^5^ cells) or in a 24 well plate 500 μl/well (approximately 1 × 10^6^) and incubate at 37°C in a humidified incubator.
- Stimulate cells as required.

##### 1.2.4.4 Data analysis.

For data analysis, we refer to the protocols described in Section 1.2.2 of the guidelines.

##### 1.2.4.5 Pitfalls.

The major hurdles during FACS-based purification of cells are the viability, constitutive activation, and yield of purified cells. Using the protocol described above, we were able to sort totally viable pDC when analyzed directly after sorting. After 16 h re-culturing in pDC-medium we observed around 90% viable pDC. The sorting process does not constitutively activate FACS-purified pDC. After activation of TLR9 with CpG, sorted pDC produce tremendous amounts of type I IFN. In order to maintain the cell viability, we suggest the following precautions.

- Do not centrifuge cells for longer periods than described in the protocol. In addition, readily resuspend the cells after centrifugation. Keeping cells for longer times in pellet will reduce the cell viability.
- Do not extend the total handling time longer time 8 h for the whole procedure. Keeping cells for longer than 8 h out of culture considerably reduces the cell viability in a time dependant manner. We suggest preparing the sorter and cells in parallel to avoid the additional waiting. Wherever possible, take the help of a second person to reduce the handling time.
- Always keep the cells cold at 4°C throughout the procedure.

Sorting sufficient cells for a reliable experiment in the given time is another challenge. Depending on the culture conditions and sort speed, the expected yield of purified pDC is around 10–15% of the total input events, when sorting pDC from C57BL/6 BM-Flt3L cultures. We suggest diluting the sample to acquire the cells at a speed of around 10,000 events/second at the lowest flow rate. However, if more pDC are required for the experiment and given that the input sample is not the limiting factor, we suggest accelerating the cell acquisition rate to 15,000 events/second at the lowest flow rate. Accelerating acquisition will increase the output yield in a given time. However, it will lower the sorting efficiency.

##### 1.2.4.6 Top tricks.

###### Related to Steps 21–22:

If there is no staining of compensation beads, it may be because of the use of improper beads or anti-bodies. Use CompBeads Anti-Mouse Ig, κ particles, for antibodies generated in mouse and Anti-Rat/Hamster Ig, κ particles for rat and hamster antibodies. However, some hamster antibody clones have a very low affinity for Anti-Rat/Hamster Ig, κ particles. Use an alternate to hamster antibody clones for compensation.

###### Related to Step 23:

A washing step is not required for compensation controls. Prepare the beads on the day of use.

###### Related to step 25:

Always filter the cell samples before acquiring. The cell aggregates result clogging of the sample line while acquiring samples. This happens more frequently when sorting cells containing a higher number of nonviable cells. We recommend good resuspending the cells in the FACS buffer containing EDTA and filtering the cells prior to acquisition. Further diluting cells to 5×10^6^/ml may help to avoid the rebuilding cells aggregates and clogging of the sample line.

###### Related to step 26:

For a large number of cells, collect the sorted cells in FCS coated 15 ml conical-bottom collection tubes. When using 85-micron nozzle, one can collect up to 5×10^6^ cells in a 15 ml pre-coated conical-bottom tube. Up to 2×10^6^ cells could be collected in a 5 ml FACS tubes. If a lower cell number is required one may use the pre-coated, sterile, 5 ml FACS tube containing 500 μl FCS. Further options are using 1.5 ml tubes or multiwell plates.

###### Related to Sep 31:

In stimulated BM-Flt3L cultures, cell surface expression of SiglecH is downregulated whereas cell surface mPDCA-1 expression increases. We suggest sorting resting pDC from Flt3L cultures. If activated pDC must be sorted for any particular application, the gates for SiglecH and mPDCA-1 should be adjusted accordingly.

###### Related to Step 42:

The purified pDC should be incubated at least for one hour at 37°C before the start of the bioassay/activation. This allows the cells to recover from the sort stress and maintain active metabolism before activation. Depending on the time and assay type, this incubation time may be extended up to 24 h. However, after 24 h the number of dying pDC may influence the outcome of the experiment.

## 2 Human DC

### 2.1 Preparation of source

#### 2.1.1 Isolation of monocytes from human peripheral blood

##### 2.1.1.1 Introduction.

The mammalian cell type which is specialized in initiating all adaptive immune response was described in 1973 by Ralph Steinman and Zanvil Cohn and termed dendritic cells (DC) [74]. These cells are sentinels of the immune system [75, 76], watching over the induction of immune responses and the maintenance of tolerance. They are also the link between innate and adaptive immunity [77]. However, ex vivo-isolation of human DC is demanding and often only small numbers of living DC can be generated (see below under 2.2.3). An alternative source to generated DC in larger numbers are blood monocytes, which were shown to differentiate toward DC in presence of granulocyte/macrophage colony-stimulating factor plus IL-4 already in 1994 [78]. A practical protocol to generate these DC in sufficient numbers was published two years later [79]. Although these DC are differentiated in vitro, they have an in vivo counterpart [80]. Different approaches exist for isolating the monocytes, and differentiating them into DC. Two methods have emerged as feasible and practical: A) antibody-based isolation with magnetic beads and columns, which separates the monocytes from all other cells via their expression of different surface antigens or B) plastic adherence, which employs the monocytes capability to adhere to tissue culture-treated plastic. Other means, like e.g. counter flow elutriation exist, but are usually only used for clinical production due to scale and requirement of equipment [81]. In this section, both methods (A and B), which partially overlap, are described. Note that protocol B requires purer cells (i.e. fewer platelets) and hence a more complex washing procedure

##### 2.1.1.2 Materials.

###### 2.1.1.2.1 Reagents.

- Human Peripheral Blood from healthy donors
- Density gradient medium: Lymphoprep (Stemcell) or Ficoll-Paque.
- PBS
  A Human CD14^+^ Isolation Kit (Miltenyi Biotec or Stemcell).
  A Staining buffer: PBS, 0.5% human serum, and 2 mM EDTA. A LS columns if using the Miltenyi kit (Miltenyi Biotec).
  B PBS-EDTA (PBS with 1 mM EDTA) B RPMI1640
  B DC-Medium (RPMI with 1 % heat-inactivated human plasma, 2 mM L-glutamine, and 20 mg/l gentamycin)
  B Cell culture dishes (10 cm)

###### 2.1.1.2.2 Equipment.

- Cell culture centrifuge
- Cell culture incubator
- Cell counter
  A Separator magnet (Miltenyi Biotec or Stemcell).

##### 2.1.1.3 Step-by-step sample preparation.

###### Protocol A

####### PBMC purification

1. Add 16 mL of density gradient medium to a 50 mL tube.
2. Slowly pour 30 mL of blood on top of the density gradient medium in each tube. Top up to 45 mL with PBS if necessary.
3. Centrifuge the tubes 15 min at 800 × *g* at room temperature, without brake.
4. Remove the leukocyte ring with a 5 mL pipette. If using several tubes for the blood of the same donor, pool all the leukocytes in one 50 mL tube.
5. Fill up the 50 mL tube with PBS.
6. Centrifuge 5 min at 500 × *g* at 4°C.
7. Discard the supernatant carefully. Resuspend the cells in 50 mL of PBS.
8. Repeat steps 6 and 7 twice, for a total of three washes.
9. Count the PBMC.

####### Monocyte isolation

- Estimate the number of required PBMC. Monocytes represent 5–10% of PBMC.
- Centrifuge the PBMC at 450 × *g* for 5 min. at 4°C.
- Follow the manufacturer’s instructions for the isolation of monocytes.
- Count the cells.

###### Protocol B

####### Before you start:

Allow RPMI, PBS, and DC-Medium to reach room temperature. Have PBS-EDTA at 4°C

####### PBMC purification

1. Add 16 mL of density gradient medium to a 50 mL tube.
2. Slowly pour 30 mL of blood on top of the density gradient medium in each tube. Top up to 45 mL with PBS if necessary.
3. Centrifuge the tubes at 22°C, 30 min at 530 × *g*, without brake.
4. Remove the leukocyte layer with a 10 mL pipette. If using several tubes for the blood of the same donor, pool leukocytes from 2 tubes into one 50 mL tube.
5. Fill up the 50 mL tubes with PBS-EDTA.
6. Centrifuge at 4°C, 15 min at 285 × *g* (low brake).
7. Discard the supernatant carefully. Resuspend the cells in 5 ml of PBS-EDTA and again pool leukocytes from two tubes into one tube and top up to 50 ml with PBS-EDTA.
8. Centrifuge at 4°C, 10 min at 190g (low brake).
9. Discard the supernatant carefully. Resuspend the cells in 5 mL of PBS-EDTA, pool leukocytes from 2 tubes into one tube, and top up to 50 ml with PBS-EDTA.
10. Centrifuge at 4°C, 12 min at 115 × *g* (no brake).
11. Discard the supernatant carefully. Resuspend the cells in 5 mL of RPMI, pool all leukocytes into one tube, and top up to 50 ml with RPMI.
12. Remove sample for counting
13. Centrifuge at 22°C, 10 min at 150 × *g*.
14. Count the PBMC.
15. Resuspend cells at 4 × 10^6^ cells/ml in DC-medium, round volume up to the next multiple of 10 mL.
16. Seed 10 ml of cells to tissue culture dishes
17. Transfer to incubator for and allow cells to adhere for 1 to 2 h
18. Remove the supernatant. This contains the non-adherent fraction (NAF) of the cells consisting mainly of T, B, and NK cells, which can be used for further experiments
19. Rinse the dishes firmly with RPMI. The monocytes have now adhered to the surface, and will not come off.
20. Add 10 ml of DC-medium per dish You now have adherent monocytes, which can be differentiated into DC

#### 2.1.2 Isolation of human stem cells from peripheral blood, cord blood, or BM

##### 2.1.2.1 Introduction.

As cDC1, cDC2, and pDC originate from CD34^+^ HSC it is possible to use these cells to generate the DC subpopulations. The isolation of CD34^+^ HSC is currently carried out in 3 different ways, which are described in detail below. Peripheral blood (PB), umbilical cord blood (CB), and BM serve as sources for the isolation of CD34^+^ HSC. Peripheral blood has the disadvantage that the number of CD34^+^ HSC is very low, i.e. *<*0.1%. The percentage of CD34^+^ HSC is significantly higher in CB with 0.5–1.5% and highest in BM with 0.5–5% [82, 83]. The possibility of obtaining PB or CB from healthy donors is significantly higher than BM. BM from patients undergoing hip replacement surgery is a good option for this purpose. However, one should bear in mind that in the latter case, the subjects are usually older and may have co-morbidities, whereas CB HSC likely have skewed differentiation potentials compared to adult BM HSC. In all three cases, the material should be processed as quickly as possible and requires enrichment of the CD34^+^ HSC via density gradient centrifugation, magnetic beads, and additional FACS as previously described by many groups [83, 84].

BM is a semi-solid tissue located in the cancellous parts of bones and is the main site of adult hematopoiesis. Besides HSC and mesenchymal stem cells (MSC) the BM contains leukocytes, granulocytes, stromal cells, and marrow adipose tissue. BM mononuclear cells (BM MNC), including stem and progenitor cells as well as leukocytes, are separated from erythrocytes and granulocytes by density gradient centrifugation. The PB, CB, or BM sample is layered in a tube on a solution of high molecular weight sucrose polymers called density gradient medium. Following centrifugation, the sample is separated into its components according to their densities resulting in erythrocytes at the bottom due to their higher density, leukocytes and platelets as buff-colored layer in the middle, and plasma as pale-yellow fluid on top.

Here, we describe the purification of CD34^+^ HSC from PB, CB, or BM for downstream simultaneous *in vitro* generation of e.g. cDC1, cDC2, and pDC.

##### 2.1.2.2 Materials.

###### 2.1.2.2.1 Reagents.

- Natrium-Heparin (Ratiopharm, Ulm, Germany)
- Cell culture Dulbecco’s PBS (DPBS; Life Technologies, Carlsbad, USA)
- Biocoll cell-separation solution (Merck Millipore, Billerica, USA)
- FBS (Sigma-Aldrich, St. Louis, USA) heat-inactivated
- Freezing-medium: FBS containing 10% DMSO (Honeywell, Morristown, USA)
- Thawing medium: RPMI-40 (Sigma–Aldrich, St. Louis, USA), 10 % FCS (Sigma-Aldrich, St. Louis, USA) containing 50 KU DNaseI (Merck-Millipore, Billerica, USA)
- MACS-buffer: DPBS w/o Mg^2+^, Ca^2+^ (Life Technologies, Carlsbad, USA) + 2 mM EDTA (Merck-Millipore, Billerica, USA) + 0.05 % BSA (Biomol, Hamburg, Germany)
- CD34+-UltraPure-Kit (Miltenyi Biotec, Bergisch Gladbach, Germany)
- FACS-buffer: DPBS w/o Mg^2+^, Ca^2+^ (Life Technologies, Carlsbad, USA) + 1 % FCS (Sigma-Aldrich, St. Louis, USA) + 0.09 % NaN_3_ (Sigma-Aldrich, St. Louis, USA) + 2 mM EDTA (Merck-Millipore, Billerica, USA)
- human IgG (100 μg/ml in FACS buffer; Sigma-Aldrich, St. Louis, USA)
- MS-5-Medium: Alpha-Medium (Merck-Millipore, Billerica, USA), 10 % FCS (Sigma-Aldrich, St. Louis, USA), 100 U/ml Penicillin-Streptomycin (Thermo Fisher Scientific, Waltham, USA), 2 mM l-Glutamine (Thermo Fisher Scientific, Waltham, USA), 2 mM Natrium-Pyruvate (Sigma-Aldrich, St. Louis, USA).

###### 2.1.2.2.2 Equipment.

- 100 μm cell strainer (Greiner Bio-One, Kremsmünster, Austria)
- 70 μm cell strainer (Greiner Bio-One, Kremsmünster, Austria)
- Mr. Frosty^™^ freezing container (Thermo Scientific, Waltham, USA)
- LS-MACS-column (Miltenyi Biotec, Bergisch Gladbach, Germany)
- MACS-separator (Miltenyi Biotec, Bergisch Gladbach, Germany)
- Aria IIIu cell-sorter (Becton Dickinson Biosciences, Franklin Lakes, USA) or equivalent
- 5-ml polypropylene round-bottom tube with a cell-strainer cap (Becton Dickinson Biosciences, Franklin Lakes, USA)

##### 2.1.2.3 Step-by-step sample preparation.

###### Isolation of BM mononuclear cells (BM-MNC).

Gain BM of HDs as residual material out of the opened bone shaft during joint replacement operations.

Transfer the BM into a 50 ml tube containing 5.000 IU of Natrium-Heparin. For the purification of BM-MNC, dilute the sample 1:2.5 fold with Dulbecco’s PBS, mix the suspension thoroughly, and pass cells through a 100 μm cell strainer to remove bone fragments and cell clumps. Next, perform the density gradient centrifugation by carefully and slowly layering 35 ml of BM cell suspension onto 15 ml of Biocoll cell-separation solution in a 50-ml conical tube. This is best achieved by tilting the 50-ml tube at a 45°C angle and slowly let the blood run down on the side of the tube onto the Biocoll layer. Centrifuge the tube at 850 × g, 30 min, RT in a swinging-bucket rotor without brake. Aspirate the plasma (upper layer) without disturbing the mononuclear cell layer. Use a sterile transfer pipette to carefully transfer the mononuclear cell layer to a new 50-ml conical tube and wash the BM-MNC twice by adding up to 40 ml of PBS, mix gently, and centrifuge at 300 × *g* and RT for 10 min RT. At this step, the BM-MNC can be cryopreserved for later use.

###### Cryopreservation of PBMCs and BM-MNC.

For Cryopreservation resuspend PBMCs and BM-MNC in ice-cold freezing-medium at a concentration of 1×10^7^ cells/ml in a sterile cryogenic micro tube and immediately freeze the cells in a freezing container for 12–24 h at −80°C before transferring it to −150°C or liquid nitrogen tank for long-term storage.

###### Thawing of PBMCs and BM-MNC.

Add 10 ml of cold thawing medium in a 15-ml conical tube to thaw one vial. Remove the vial from the liquid nitrogen tank, and hold it at room temperature until the sides are thawed but the center remains frozen. Transfer the contents of the vial into a 15-ml conical tube containing cold thawing medium. Centrifuge the cells at 480g for 5 min at 20°C. Resuspend the cells in 5 ml of thawing medium containing 200 KU DNase I staining buffer and incubate for 20 min at 4°C protected from light. Thereafter, filter (100 μm cell strainer) the cells and count them using a hemocytometer or other cell counter, and determine the viability by trypan blue stain exclusion.

###### Purification of CD34+-progenitors using magnetic beads and FACS.

Enrich CD34^+^-HSC and progenitors using a column-based cell positive separation method like CD34^+^-UltraPure-Kit according to the manufacturer’s protocol.

For further purification, the cells should be sorted by FACS. Therefore, resuspend the cells in 1 ml human IgG at a density of 3 × 10^6^ cells/ml and incubate for 20 min at RT protected from light to prevent unspecific antibody-binding. Centrifuge 350g for 10 min at 4°C and then stain the 3×10^6^ cells/100 μl in sorting buffer according to Table 10 for 30 min at 4°C protected from light. Wash the cells by adding 2 ml of sorting buffer. Centrifuge the cells at 350g for 10 min at 4°C. Resuspend the cells in sorting buffer at a density of 1 × 10^7^ cells per ml, and transfer them to a new 5-ml polypropylene round-bottom tube with a cell-strainer cap and add DAPI. Keep the sample in the dark on ice, and then sort the sample on a cell sorter using a 85 μm nozzle in a timely manner (within 1 h). Collect the CD34^+^-cells in MS-5-Medium and perform a reanalysis to determine the purification rate.

##### 2.1.2.4 Data analysis.

**Figure 19.**
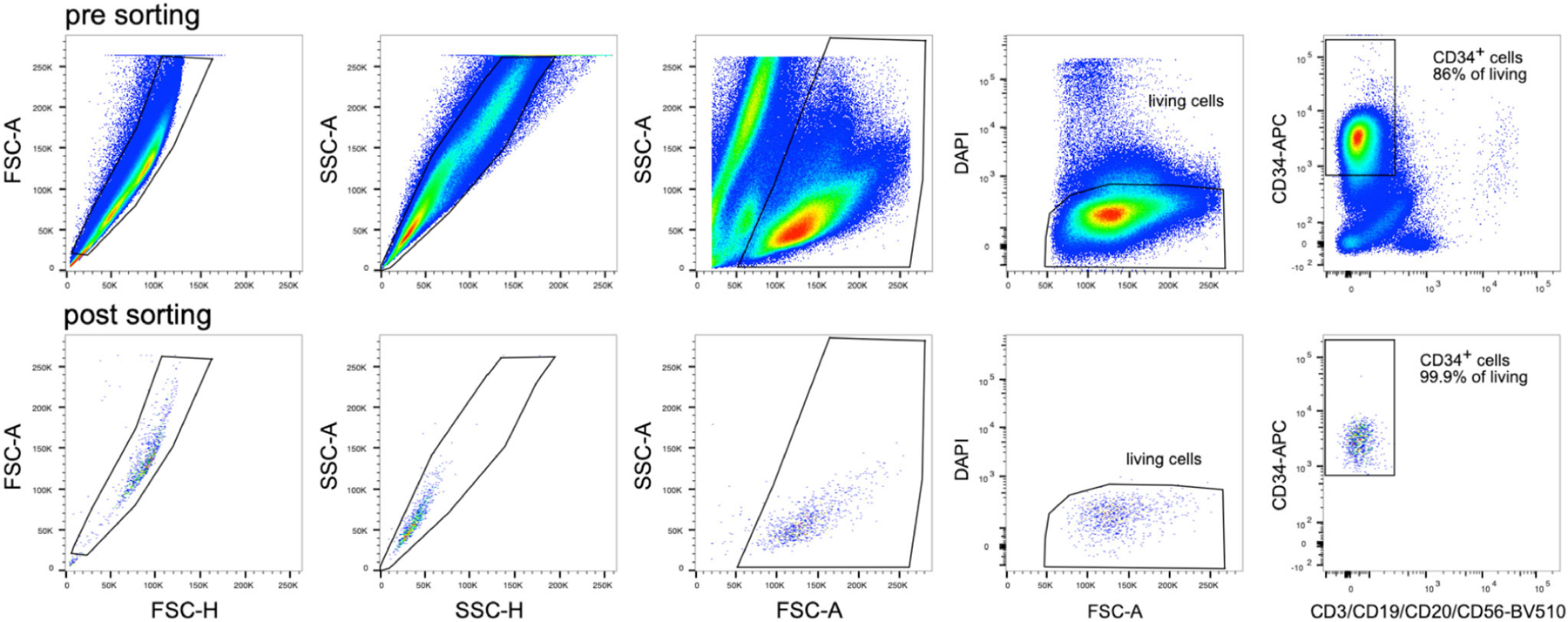
Flow cytometry data of BM-MNC after CD34^+^ bead separation/pre-sort (upper row) and after sorting CD34^+^ HSC (lower row; reanalysis).

The BM CD34^+^ HSC purity after the magnetic bead separation differs between 70 – 90% and 98–100% after the sorting (Figure 19). After sorting the yield of CD34^+^ BM HSC is between 1–5×10^5^/ml BM.

##### 2.1.2.5 Top tricks.

###### Be fast:

It is essential that the BM is stored at room temperature and processed within 24 h at the maximum. As described above, the BM-MNC can be stored at −150°C for a longer period of time.

###### Sorting conditions:

To ensure successful differentiation of CD34^+^ HSC into DC subpopulations, cell sorting should be performed under sterile conditions and not take too long, which is why we recommend pre-purification with magnetic beads. In addition, an optimized setting of sorting parameters such as sheath fluid pressure is essential for the viability of the cells.

###### CD34^+^ HSC purity:

A CD34^+^ HSC purity of *>*80% is sufficient for the generation of DC subsets in particular if there is an HSC expansion step or the DC differentiation protocol is longer than 14 days as residual leukocytes will not survive these procedures.

### 2.2 Human DC generation and quality control

#### 2.2.1 DC generation from monocytes

##### 2.2.1.1 Introduction.

Different cytokine combinations can be used to differentiate monocytes into dendritic cells [79]. The classical protocol uses GM-CSF and IL-4, but alternatively, M-CSF, IL-4, and TNFα can be used. The former reproducibly generates a very homogenous population, while the latter produces a more heterogeneous but more in-vivo-like population [85]. If clinical application is envisioned fetal bovine serum-free medium should be used.

##### 2.2.1.2 Materials.

###### 2.2.1.2.1 Reagents.

Human monocytes

- DC-medium (RPMI-Glutamax with 10% decomplemented FBS, Penicillin, and Streptomycin) or (RPMI with 1% heatinactivated human plasma, 2 mM l-glutamine, and 20 mg/l gentamycin)
- Human recombinant cytokines, premium grade: GM-CSF and IL-4, or M-CSF, IL-4, and TNF-α
- Plates treated for cell culture (10 cm)

###### 2.2.1.2.2 Equipment.

- Cell culture incubator

##### 2.2.1.3 Step-by-step sample preparation.

1. Resuspend monocytes from protocol A at 2 × 10^6^ cells/ml in DC-medium and distribute on plates (10 ml/plate), or add 10 ml of DC-medium to the adherent monocytes from protocol B.
2. Add the required cytokines: GM-CSF (1.000 IU/ml) and IL-4 (40–250 IU/ml, see Pitfalls for comment), or M-CSF (7000 IU/mL), IL-4 (40 IU/mL), and TNF-α (300 IU/mL)
3. Plate monocytes in culture plates.
4. Incubate for 5–6 days. (If your cells do not seem to develop well, you can add fresh medium and cytokines every 2 days)
5. Collect the cells in suspension. Collect the remaining cells by flushing them with PBS.

If you used adherent monocytes, the resulting immature DC are usually also quite adherent, so you may add the maturation stimulus directly to the plates and then later harvest the mature DC, which are not adherent anymore.

**Figure 20.**
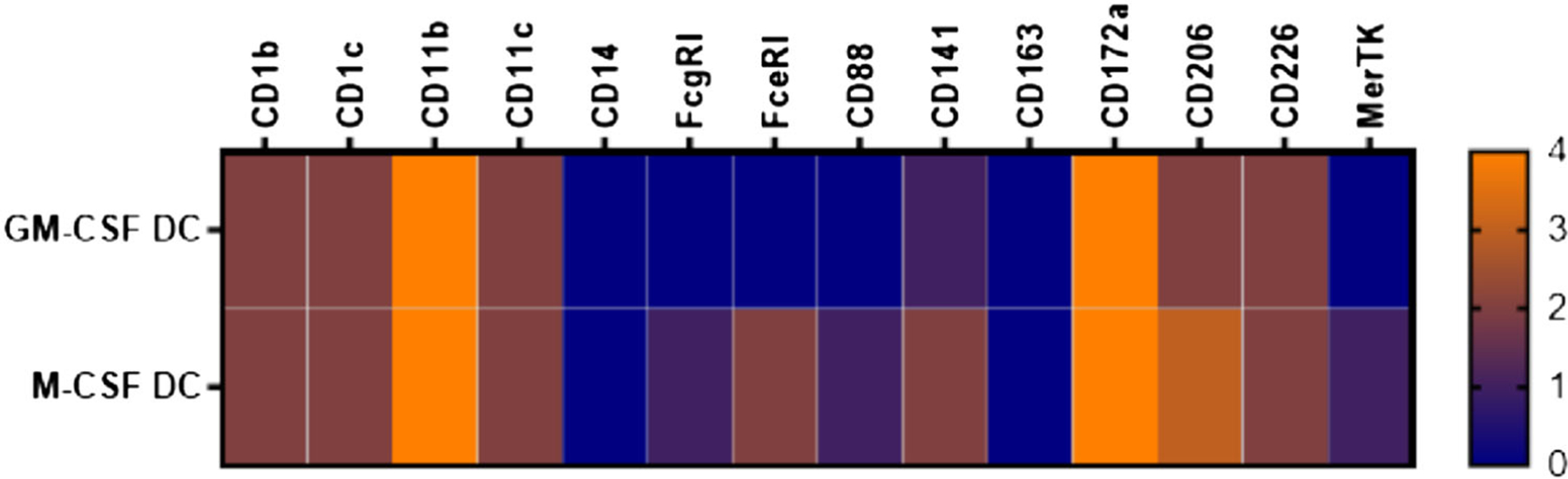
Phenotype of GM-CSF and M-CSF-generated DC. DC were generated in presence of GM-CSF and IL-4 (upper row) or M-CSF, IL-4, and TNF-α (lower row) from CD14^+^ monocytes. Then the cells were stained with antibodies specific for the indicated markers and analyzed by flow cytometry as described in [79]. Expression of the indicated proteins was categorized in 5 levels of expression (0 to 4). The corresponding histograms, except for CD1c and CD11c, were published previously by Goudot et al. [85].

Figure 20 shows a comparison between the phenotype of DC generated either with M-CSF, IL-4, and TNF-α or with GM-CSF and IL-4.

##### 2.2.1.4 Pitfalls.

The choice of cytokine cocktail should be guided by the downstream assay and the question being asked. Differentiation of monocytes using GM-CSF and IL-4 will generate a homogenous population of DC-like cells, which do not have an identified in vivo counterpart. Culturing monocytes using a cocktail of M-CSF, IL-4, and TNF-α will generate DC that resemble monocyte-derived DC found in human inflammatory clinical samples [85]. However, the outcome of this culture is a heterogenous population of macrophages, DC and undifferentiated cells.

The required concentrations of the cytokines depend on the supplier but also on the blood donor. While higher concentrations usually give more stable results, lower concentrations may be feasible for economic reasons.

Human monocytes do not proliferate in culture. The yield of these cultures is typically 60% of the number of seeded cells.

Human DC are very sensitive to LPS, hence be sure that all reagents are absolutely endotoxin-free.

When the adherence is unsatisfying, a different brand of tissue culture dishes should be tested.

##### 2.2.1.5 Top tricks.

Blood needs to be added on top of the density gradient very gently. To facilitate this step, you can use special blood separation filter tubes. These can be SepMate^™^-50 (Stemcell) or Blood-sep-filter (Dacos).

Culture medium can be refreshed at day 3 of culture by adding a small volume of complete medium containing concentrated cytokines.

Avoid frequent temperature changes, e.g. putting the cells too often from 37°C on ice and back. In our experience, handling DC at room temperature keeps them in better condition that putting them on ice.

#### 2.2.2 DC differentiation from CD34^+^ hematopoietic stem/progenitor cells (HSPC)

##### 2.2.2.1 Introduction.

This protocol uses purified CD34^+^ hematopoietic stem/progenitor cells HSPC from either peripheral blood (PB), cord blood (CB), or BM (Isolation is described in section 2.1.2) for the simultaneous generation of DC subpopulations equivalent to those found in human adult peripheral blood, namely cDC1, cDC2, and pDC. For protocols enabling to generate LCs, dermal DC, or MoDC from HSPC, please refer to the following papers [84, 86–90].

In 2004, the first protocol for simultaneous *in vitro* generation of bona fide pDC and of TLR3-expressing DC that were likely bona fide cDC1 was published [91]. In this protocol, HPSCs were cultured for up to 30 days in Yssel medium with the addition of FLT3-L and TPO. Later, Proietto et al. showed that similar culture conditions yielded cell types that had a similar phenotype to the DC subpopulations in peripheral blood, namely cDC1, cDC2, and pDC, and expressed corresponding key genes [92]. However, the yield was relatively low. Further protocols followed, combining the use of cytokines and feeder cells, and others were optimized to, among other things, recapitulate in vitro the differentiation of most immune cell lineages to study the ontogeny of these cells in terms of precursor-product relationships and underlying transcription factor networks [83, 93, 94]. In addition, protocols were adapted to increase the yield of cDC1 and pDC, firstly with the aim of facilitating the study of the functions of these subpopulations and their molecular regulation [95, 96], and secondly for clinical use for adoptive cell immunotherapy in cancer patients [97–99].

As we are not able to go into detail about the different protocols published, we here describe a protocol leading to the simultaneous differentiation of cDC1, cDC2, and pDC for their use in functional assays. Other protocols, with their recommended use, are briefly described in Table 11 and reviewed elsewhere [100].

In some of these studies, there were marked differences between the in vitro generated and PB DC subpopulations, in terms of expression of the characteristic markers that define the different DC subpopulations. These problems highlight both the need to use a uniform nomenclature and to define methods to ensure proper identification and characterization of in vitro-derived human DC subpopulations. Methodologically, in addition to accurate phenotyping, we recommend performing gene expression profiling of the sorted in vitro generated DC subpopulations, at least when initially establishing the protocol in your laboratory. In Table 12, we summarize the minimal standards recommended for ensuring the identity of the DC subsets generated in vitro from CD34^+^ HSPC.

The use of well-defined/standardized in vitro differentiated DC subpopulations will certainly contribute to our understanding of the molecular mechanisms of their development, possible manipulation, functions, and behaviors in inflammations and infections, as well as their potential clinical use as vaccines or treatments against cancer.

##### 2.2.2.2 Materials.

###### 2.2.2.2.1 Reagents and consumables.

####### 2.2.2.2.1.1 DC differentiation

MEM Alpha Medium w/o Nucleosides (αMEM) (Life Technologies, Cat no 22561–021)

Fetal Bovine/Calf Serum (FBS/FCS; test and use the same batch throughout), filtered and heat-inactivated

Mouse OP9 cell line (GFP-expressing) (ATCC, Cat no CRL-2749)

Dulbecco’s Phosphate Buffered Saline (DPBS; Sigma, Cat no D8537)

Penicillin-Streptomycin (Sigma, Cat no P0781)

Ethylenediaminetetraacetic acid (EDTA, Sigma, Cat no E7889)

Recombinant human cytokines: Flt-3 ligand (Flt3L; Immunotools, Cat no 11343305); Stem Cell Factor (SCF; Immunotools, Cat no 11343325); GM-CSF (R&D systems, Cat no CAA26822)

OP9 medium: αMEM, 20% FCS, penicillin-streptomycin

Trypsin-EDTA solution: 0.25% Trypsin, 0.53mM EDTA

DC differentiation medium: αMEM, 10% FCS, 1% penicillin-streptomycin, 100ng/ml Flt3L, 20ng/ml SCF, 20mg/ml GM-CSF

96 Well TC-Treated Microplates (round-bottom) (Corning^®^, Cat no CLS3799)

15 ml or 50 ml sterile polypropylene conical Falcon tubes (Greiner, Cat nos 188271 and 227261)

####### 2.2.2.2.1.2 Cell harvest and staining for flow cytometry or FACS purification

Dulbecco’s Phosphate Buffered Saline (DPBS; Sigma, Cat no D8537)

Ethylenediaminetetraacetic acid (EDTA, Sigma, Cat no E7889)

Fetal Bovine/Calf Serum

Ice and ice bucket

Flow buffer: PBS, 2% FCS, 2mM EDTA

FACS buffer: PBS, 0.5% FCS, 2mM EDTA

Mouse IgG from serum (Sigma, Cat no I5381)

CellTrics 50 μm, filter (Sysmex-Partec, Cat no sterile 04–004–2327; non-sterile 04–0042–2317)

Flow cytometry tubes (5 ml polystyrene or polyproylene round-bottomed tissue culture tubes) (SLS Cal no 352063; 352063)

1.5 ml Eppendorf tubes (Starlab Cat no E1415–1500)

DAPI (Sigma, Cat no 09542) - stock 1 mg/ml (use at 1:2000)

BD Horizon Brilliant Stain Buffer (BD Biosciences, Cat No 563794/566349)

Anti-mouse Ig, K/Negative Control BD^™^ CompBeads (BD Biosciences Cat no 552843)

Fluorochrome conjugated antibodies (Table 11)

BD CompBeads set anti-rat Igκ (BD Biosciences, cat. no. 552844)

Collection medium: αMEM, 10% FCS, penicillin-streptomycin

###### 2.2.2.2.2 Equipment.

Inverted Microscope

CO_2_ incubator for cell culture (37°C, 5% CO_2_)

Flow cytometer (such as LSR Fortessa X20, BD Biosciences) or Fluorescence-activated cell sorter (FACS, such as FACS Aria Fusion, BD Biosciences), for cell analysis or purification, respectively.

##### 2.2.2.3 Step-by-step sample preparation.

###### 2.2.2.3.1 OP9 Culture and plating.

1. Harvest OP9 cells: 4–24 h before isolation of CD34^+^ progenitors, defrost cryopreserved OP9 cells, or harvest cultured OP9 cells, following standard protocol for OP9 handling (available at www.atcc.org). Briefly, OP9 cells are cultured in 75cm^2^ (T75) tissue culture flasks to a density of 4 × 10^3^–1 × 10^4^ cells/cm^2^, in 15–20 ml OP9 medium at 37°C, 5% CO_2_ in air, with medium changed every 3–4 days.
2. To harvest, remove culture medium and rinse briefly with 1–2 ml Trypsin-EDTA solution to remove traces of OP9-medium (which contains serum).
3. Add 2–3 ml Trypsin-EDTA solution to the flask and observe the cell layer under an inverted microscope until the cells are dispersed (5–15 min). Add 6–8 ml OP9 medium and gently pipette to aspirate cells.
4. Transfer the cells and medium to a 15 ml Falcon tube and spin at 125 × *g* for 10 min. Discard the supernatant and resuspend the cell pellet in fresh, pre-warmed (37°C) OP9 medium at a concentration of 2.5 × 10^4^ cells/ml.
5. Aliquot 200 μl (5000 cells) per well into 96 well U-bottomed tissue-culture treated plates and incubate at 37°C, in 5% CO_2_.

###### 2.2.2.3.2 DC differentiation from human PB, BM, or cord blood CD34^+^ haematopoietic stem/progenitor cells (HSPC).

1. Following sorting or magnetic bead purification of CD34^+^ HSPC (see section 2.1.2), count and resuspend the cells in pre-warmed (37°C) DC differentiation medium at a concentration of 1.5 × 10^4^ cells/ml. A CD34^+^ expansion step may be performed before DC differentiation [95].
2. For each well of the 96-well U-bottom plate, containing pre-seeded OP9 cells, gently pipette off the medium from above the OP9 layer and replace with 200 μl of CD34^+^ cell suspension (3000 cells). Ensure the OP9 cells never dry out. Return the plate to the incubator.
3. On day 6, from each well carefully remove the top 100 μl of medium and replace with 100 μl pre-warmed (37°C) DC differentiation medium.
4. Cells can be harvested at days 12–21. If harvesting after Day 12 or Day 18, replace 100 μl of medium with 100 μl fresh DC differentiation medium on Days 12 and 18.

###### 2.2.2.3.3 Harvesting differentiated DC.

1. Prepare all reagents and cool solutions to 4°C before starting.
2. Remove the 96-well U-bottom plate containing OP9 and differentiated cells from the incubator and place on ice.
3. For each well, using a 200 μl pipette, gently aspirate the cells from the bottom of the well and pipette through a 50-micron filter into a 5 ml round bottom flow tube (polystyrene for flow cytometric analysis, polypropylene for downstream FACS purification).
4. Wash the well with 200 μl cold (4°C) DPBS and transfer through the filter to the flow tube. Repeat this step a further two times. The plate can be examined under an inverted microscope to ensure the majority of cells are harvested. Repeat for any remaining wells. Up to 5 wells can be harvested into 1 flow tube (total volume 4 ml)*.
5. To pellet the cells, spin at 500 × *g* for 5 min, and remove the supernatant.
6. For flow cytometric analysis, use cold (4°C) Flow buffer for subsequent steps. For FACS purification of DC subsets, use cold (4°C) FACS buffer with lower FCS concentration. Resuspend the cell pellet in 50 μl of the appropriate buffer.

*For flow cytometric analysis, cells may be washed and subsequently stained in the 96 well plate.

###### 2.2.2.3.4 Phenotypic identification of differentiated DC and/or purification for downstream analysis.

1. To reduce non-specific antibody binding, to each flow tube add 3 μl mouse Ig and leave at 4°C for 10 min.
2. Use a 1.5 ml Eppendorf tube to make up an antibody cocktail as detailed in Table 11, with sufficient volume to stain all tubes. BD Horizon brilliant stain buffer is used to reduce the interactions between Brilliant Violet/Ultraviolet dyes. Add the equivalent of 50 μl/tube brilliant stain buffer to the antibody cocktail.
3. Pipette the appropriate volume of antibody cocktail into the flow tubes containing cells, mix gently, and incubate for 30 min at 4°C in the dark.
4. Top up the flow tubes with 3–4 ml cold Flow or FACS buffer and spin at 500 × *g* for 5 min.
5. Remove the supernatant and resuspend in 200 μl flow buffer or 500–1000 μl FACS buffer for flow cytometric analysis or FACS purification, respectively. Keep in the cold and dark until analysis.
6. Prepare a single cell staining tubes with each conjugated anti-body and compensation beads, according to manufacturers’ instructions, and store at 4°C in the dark.
7. For FACS purification, prepare the collection tubes by coating 1.5 ml Eppendorf tubes or 5 ml flow cytometry tubes with FCS and discard the FCS. Add 250 μl or 500 μl aMEM+10% FCS to 1.5 ml Eppendorf tubes or flow tubes, respectively, and store on ice.
8. Add DAPI (1:2000 of stock) to the cell suspensions 5 min prior to flow cytometry or FACS sorting.
9. Immediately prior to FACS purification, filter the cell suspensions through 50micron filters into new flow/sort tubes.

##### 2.2.2.4 Dendritic Cell Identification.

**Figure 21.**
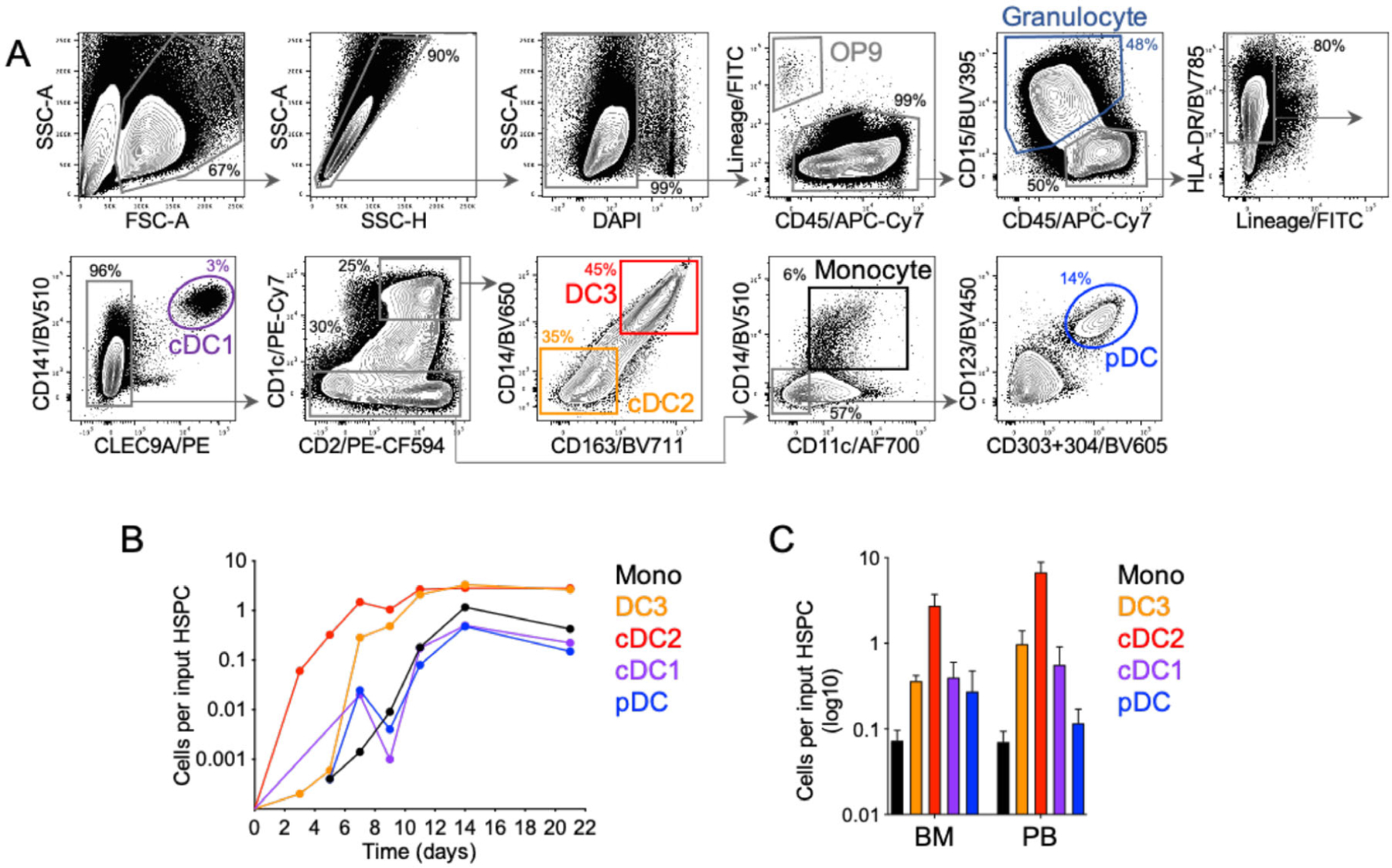
Flow cytometric analysis and DC output of CD34^+^ differentiation. (A) Flow cytometric analysis of the culture at Day 21, using LSR-Fortessa X20 (BD biosciences) and the panel described in Table 12. Debris, doublets, and dead cells are first excluded, followed by OP9 (GFP^+^ in the FITC channel and CD45^−^) and CD45^low^CD15^+^ granulocyte precursors. Selecting lineage (CD3, 16, 19, 20, 34, 56) negative HLA-DR^+^ cells excludes lymphoid lineages and undifferentiated cells and selects antigen-presenting cells. DC subsets may then be identified by sequential gating: cDC1, CLEC9A^+^CD141^+^; cDC2, CD1c^+^CD2^+^(CD163^−^CD14^−^), DC3, CD1c^+^CD2^+^CD14^+^CD163^+^; Monocytes, CD11c^+^CD14^+^ (CD1c-CD2^−^); pDC, CD123^+^CD303/304^+^. Dimensionality reduction analyses (such as tSNE or UMAP) may also be used to visualize the discrete populations. (B) DC generation in culture at Days 3, 5, 7, 9, 11, 14, and 21, expressed as the number of subset-specific DC generated per CD34^+^ HSPC seeded at Day 0. Cells were identified phenotypically as shown in (A). (C) Number of DC generated from CD34^+^ HSPC isolated from BM or peripheral blood (PB), expressed as the number of subset-specific DC generated per CD34^+^ HSPC seeded at Day 0.

Accurate identification and verification of DC subsets are critical for downstream analysis. For phenotypic analysis, each subset should ideally be identified by a minimum of two positive markers and the appropriate negative expression of other subset-specific antigens. Using the above panel, subsets can be identified as follows (Fig. 21A):

Granulocyte precursors: CD45^low^, CD15^+^

cDC1: CLEC9A^+^CD141^+^ (CD2^−^CD11c^low/+^CD123^−^CD14^−^CD1c^+^ in this system).

cDC2: CD1c^+^CD2^+^ (CD11c^+^CD14^−^CD163^−^CLEC9A^−^CD123^−^).

This population contains CD5^+^ cells, if included in the panel. DC3: CD1c^+^CD2^+^CD14^+^CD163^+^(CD11c^+^CLEC9A^−^CD123^−^).

Monocytes: CD11c^+^CD14^+^CD1c^−^CD2^−^(CLEC9A^−^CD123^−^). This population is CD88^+^ if included in the panel.

pDC: CD303/304^+^CD123^+^CD2^+/−^(CD11c^−^CD1c^−^CD14^−^CLEC9A^−^).

Additional or alternative markers may be used as summarized in Table 13.

When plating 3000 CD34^+^ cells/well, cultures should generate a two- to fivefold increase in CD45^+^ cells at day 14 and twofold to 4.5-fold at day 21, with approximately equivalent numbers of cDC2 and DC3 (approximately 1:3 and 1:2.8 CD34^+^ to DC at d 14 and d 21, respectively), cDC1 and pDC (1:0.5 and 1:0.2 at days 14 and 21, respectively) and 1:1.1 and 1:0.4 monocytes at days 14 and 21, respectively (Fig. 21B).

##### 2.2.2.5 Transcriptional and functional validation.

1. Transcriptional profiling: the gene expression profile of DC subsets generated from CD34^+^ HSPC in vitro should be verified by RNA-Seq (single cells or FACS-purified bulk populations), other gene expression platforms, or qPCR for subset-specific marker genes, summarised in Table 13
2. DC subset identity may be verified in functional assays including cytokine elaboration in response to TLR agonists and naïve T cell activation or polarisation [84, 95, 96, 101].

##### 2.2.2.6 Pitfalls.

1. FCS batch: the FCS is crucial for a good and consistent yield of DC subpopulations. We therefore recommend testing several lots of FCS for culture optimization and then using the same lot for an entire series of experiments.
2. DC identification: as stated above, it is necessary to show the exact identity of the DC types generated in vitro. Therefore, we propose the following guidelines for verifying identity as shown in Table 12. These experiments should be performed at the beginning of the DC culture comparatively with PB-DC or tissue DC subsets.
3. Feeder layer cell lines should be regularly checked to ensure they remain free from infection with mycoplasma. OP9 readily differentiate into adipocytes (visualized as larger, vacuolated cells) after large numbers of passages or long periods of culture at confluency [102]. It is therefore important to store sufficient aliquots of low passage number cells for experimental series, and to replate cells just prior to confluency.

##### 2.2.2.7 Top tricks.

1. Scaling: the culture can be scaled to larger wells, proportionally increasing the number of OP9 and CD34^+^ cells seeded.
2. Imaging the culture: the use of larger flat-bottomed wells can aid imaging of the culture, providing sufficient materials are available for scaling
3. A CD34^+^ expansion step can be added after CD34^+^ cell isolation, to increase the number of DC generated from a starting CD34^+^ population [95].
4. Similar numbers and proportions of DC subsets can be generated from CD34^+^ cells isolated from peripheral blood (Fig. 21C). As CD34^+^ cells represent *<*0.01% of peripheral blood mononuclear cells, empirically, a minimum of 40 ml blood is necessary to isolate sufficient cells for differentiation at the scale described above, without a CD34^+^ expansion step.
5. Feeder cells dramatically increase the yield of the DC subsets. However, the underlying mechanisms are not well known. The use of OP9, a murine BM stromal cell line derived from the CSF-1^−/−^Osteopetrotic (op/op) mouse, limits the exposure of the culture to M-CSF, therefore minimizing the generation of monocytes and monocyte-derived cells.
6. Cytokines: The output of generated DC is further increased by the combination of SCF, FLT3-L, and TPO, as this promotes the optimal expansion of HSC prior to DC differentiation [103]. For the differentiation of pDC and cDC from HSC, FLT3-L is the most critical cytokine. GM-CSF and IL-4 can promote the differentiation of cDC1 but also the differentiation of cMo into MoDC [78, 104]. Therefore, when adding GM-CSF and IL-4 to HSPC cultures to generate pDC and cDC, the timing and dose are critical. Too early or too concentrated addition can lead to increased differentiation toward monocytes/macrophages at the expense of DC subtypes.

##### 2.2.2.8 Summary table.

#### 2.2.3 Human cDC and pDC from PBMCs

##### 2.2.3.1 Introduction.

In human peripheral blood, at least two major subpopulations of DC can be found, conventional DC (cDC) and plasmacytoid DC (pDC). cDC can be further subdivided into type 1 cDC (cDC1) and type 2 cDC (cDC2). The blood DC subsets have unique transcriptional profiles and functional characteristics [107, 108, 118]. pDC are key effectors of innate immune responses due to their capacity to produce large amounts of type I IFNs upon TLR ligation [119]. cDC produce IL-12 in response to microbial stimulation and can induce potent Th1 responses and CTL responses [120]. cDC1 are specialized in cross-presentation of antigens derived from necrotic cells, such as tumor cells, and are potent producers of IFN*λ* in response to TLR3 ligation [121, 122]. However, all three DC subsets can induce antigen-specific CD4^+^ and CD8^+^ T cell activation and have the capacity cross-present extracellular antigens to CD8^+^ T cells [123].

Studying naturally circulating blood DC is challenging, since, compared to monocytes and other leukocytes, the frequency of circulating blood dendritic cells is extremely low, ranging range from on average 1% of peripheral blood leukocytes for cDC2, to 0.5% for pDC and as low as 0.05% for cDC1. The isolation of DC is based on the expression of (combinations of) cell surface markers. Whereas all DC subsets express high levels of HLA-DR, pDC specifically express CD123, Blood Dendritic Cell Antigen (BDCA)2, and BDCA4 (CD304). cDC express the myeloid marker CD11c, which is absent on pDC, and in addition, cDC2 express BDCA1 (CD1c) and cDC1 express BDCA3 (CD141), Clec9a, and the chemokine receptor XCR1 [124]. Circulating DC subsets can be isolated from peripheral blood by a number of distinct methodologies, all based on receptor expression profiles. Each method has its advantages and disadvantages with respect to yield and purity of the cells. The highest purity of isolated DC is obtained when PBMCs are first depleted from monocytes, B cells, T cells, and NK cells. After lineage depletion, DC subsets can be isolated either by MACS or by fluorescence-activated cell sorting (FACS). MACS sorting of pDC is generally performed with anti-BDCA4 coated magnetic beads after removal of adherent monocytes as monocytes can express intermediate levels of BDCA4 upon encountering type I IFNs. Although pDC specifically express BDCA2, this marker is often neglected for isolation purposes as it negatively affects TLR-induced type I IFN secretion. cDC1 cells are positively selected with BDCA3-coated magnetic beads. Since B cells also express the cDC2 marker BDCA1, cDC2 cells are positively selected with BDCA1-coated magnetic beads after a depletion step of CD19 positive B cells. In principle, FACS sorting of DC subsets is based on the same markers as MACS sorting. However, depending on the laser settings of the FACS sorter, more DC markers can be included to increase purity.

##### 2.2.3.2 Materials.

###### 2.2.3.2.1 Reagents.

####### Lineage depletion

- Anti-CD3 Microbeads (Miltnenyi Biotec)
- Anti-CD14 Microbeads (Miltnenyi Biotec)
- Anti-CD19 Microbeads (Miltnenyi Biotec)
- Anti-CD56 microbeads (Miltenyi Biotec)
- FcR blocking reagent (Miltnenyi Biotec)
- LD columns (Miltnenyi Biotec)
- Washing buffer consisting of PBS supplemented with 2 mM EDTA and 0.5 % human or BSA

####### MACS sorting

- Anti-CD1c biotin (Miltenyi Biotec)
- Anti-biotin microbeads (Miltenyi Biotec)
- Anti-CD141 microbeads (Miltenyi Biotec)
- Anti-CD304 microbeads (Miltenyi Biotec)
- MS columns (Miltenyi Biotec)
- LS columns (Miltenyi Biotec)
- Washing buffer consisting of PBS supplemented with 2 mM EDTA and 0.5% human or BSA

####### FACS sorting

- FITC-conjugated anti-Lin1 antibody cocktail (BD Biosciences)
- PE-Cy7-conjugated anti-HLA-DR (BD Biosciences)
- BV421-conjugated anti-CD1c (Biolegend)
- APC-conjugated anti-CD141 (Milteni Biotec)
- PE-conjugated anti-BDCA4 (Miltenyi Biotec).
- Washing buffer/staining buffer consisting of PBS supplemented with 2 mM EDTA and 0.5 % human or BSA

####### Cell culturing

- X VIVO 15 medium (Lonza)
- TexMACS medium (Miltenyi Biotec)
- Human serum (Sigma Aldrich)
- TLR ligands or other DC activation stimuli

####### Flow cytometry

- FcR blocking reagent (Miltenyi Biotec)
- Propidium iodide (Miltenyi Biotec)
- Staining buffer: any PBS-based buffer with BSA, for instance AutoMACS Running buffer (Miltenyi Biotc)
- CD370 (Clec9a)-Viobright FITC, 89F (Miltenyi Biotec)
- BDCA2-PE, AC144 (Miltenyi Biotec)
- CD14-PERCP, TÜK4 (Miltenyi Biotec)
- CD20-PERCP, LT20, (Miltenyi Biotec)
- CD1c-PE-Vio770, AD5–8E7 (Miltenyi Biotec)
- CD141 (BDCA3)-APC, REA674 (Miltenyi Biotec)
- CD123-APC-Vio770, AC145 (Miltenyi Biotec)
- FcɛRI-BioBlue, CRA1 (Miltenyi Biotec)
- CD45-VioGreen, 5B1 (Miltenyi Biotec)
- HLA-ABC-APC, REA230 (Miltenyi Biotec)
- HLA-DR,DP,DQ-APC, REA332 (Miltenyi Biotec)
- CCR-APC, REA108 (Miltenyi Biotec)
- CD80-APC, 2D10 (Miltenyi Biotec)
- CD83-APC, HB15 (Miltenyi Biotec)
- CD86-APC, FM95 (Miltenyi Biotec)
- CD141 (BDCA3)-PE, REA674 (Miltenyi Biotec)
- CD1c (BDCA1)-Viobright-FITC, AD5–8E7 (Miltenyi Biotec)

###### 2.2.3.2.2 Equipment.

Include details of the manufacturer.

- Any FACS sorter with appropriate laser and filter settings
- Any flow cytometer with appropriate laser and filter settings
- MiniMACS Separator (Miltenyi Biotec)
- MidiMACS Separator (Miltenyi Biotec)

##### 2.2.3.3 Step-by-step sample preparation.

###### 2.2.3.3.1 Lineage depletion (optional).

####### *Lineage depletion* (optional)

1. Resuspend PBMCs in a buffer containing a cocktail of anti-CD3-, anti-CD14-, anti-CD19-, and anti-CD56 microbeads and anti-FcR blocking reagent, according to dilutions recommended by the manufacturer. Do not the FcR blocking reagent if the isolated DC will be used for phenotypical or functional studies of Fc receptors.
2. Mix well and incubate the cell suspension for 30 min at 4°C. Shake every 5 min.
3. Centrifugate the cell suspension and resuspend the cell pellet in washing buffer at a concentration of 100×10^6^ cells per ml.
4. Place MACS LD columns in the appropriate magnet and prewet the columns with 1 ml of washing buffer per column; one column per 100×10^6^ PBMCs.
5. Apply the cell suspension to the column, 1 ml cell suspension containing 100×10^6^ PBMCs per column, and let the sample run through the column. Wash the column three times with 1 ml of washing buffer to completely elute the lineage-negative fraction from the column. Collect the flowthrough negative fraction, which contains the DC, in a tube.
6. Optionally, repeat steps 3–5 for an additional depletion step with new LD columns for higher purity.
7. Collect the negative fraction and proceed with MACS sorting or FACS sorting of DC subsets.

###### 2.2.3.3.2 MACS sorting.

Steps 1–7 are only performed for cDC2 isolation; for pDC and cDC1 isolation, start with step 8.

1. If lineage-depletion has not been performed, first deplete B cells with CD19 microbeads. If lineage depletion has already been performed, steps 1–7 can be skipped.
2. For B cell depletion, resuspend PBMCs in a buffer containing anti-CD19 microbeads and anti-FcR blocking reagent, according to dilutions recommended by the manufacturer.
3. Incubate the cell suspension for 30 min at 4°C
4. Centrifugate the cell suspension and resuspend the cell pellet in washing buffer at a concentration of 100×10^6^ cells per ml.
5. Place MACS LD columns in a magnet and prewet the columns with 1 ml of washing buffer per column; one column per 100×10^6^ PBMCs.
6. Apply the cell suspension to the column, 1 ml cell suspension containing 100×10^6^ PBMCs per column, and let the sample run through the column. Wash the column three times with 1 ml of washing buffer to completely elute the CD19-negative fraction from the column. Collect the flowthrough negative fraction, which contains the DC, in a tube.
7. Optionally, repeat steps 4–6 for an additional depletion step with new LD columns for higher purity.
8. Centrifugate the negative fraction after lineage depletion or B cell depletion and add anti-CD1c-biotin beads (for cDC2 isolation), anti CD141 microbeads (for cDC1 isolation), or anti-CD134 microbeads (for pDC isolation) in a concentration recommended by the manufacturer.
9. Incubate the cell suspension for 15 min at 4°C.
10. Wash the cells with 50 ml washing buffer and centrifugate the cell suspension.
11. Add anti-biotin microbeads in the concentration recommended by the manufacturer and mix well.
12. Incubate the cell suspension for 15 min at 4°C. Shake every 5 min.
13. Add washing buffer to a total volume of 50 ml and centrifugate the cell suspension.
14. Resuspend the cell pellet in 1 ml of washing buffer.
15. Place a MACS LS column in an appropriate magnet and prewet the column with 1 ml of washing buffer.
16. Apply the cell suspension to the LS column and let the cells pass through the column. If only one DC subset is isolated, discard the supernatant. Otherwise, collect the supernatant for subsequent isolation of other DC subsets.
17. Wash the LS column three times with 1 ml washing buffer.
18. Remove the column from the magnet and flush the DC out of the column with 1 ml washing buffer. Collect the cells in a tube.
19. To increase purity, repeat steps 4–8 with a MACS MS column (optional).

###### 2.2.3.3.3 FACS sorting.

1. Label the remaining cell fraction after lineage depletion in staining buffer with FITC-conjugated anti-Lin1 antibody cocktail, PE-Cy7-conjugated anti-HLA-DR, BV421-conjugated anti-CD1c, APC-conjugated anti-CD141, and PE-conjugated anti-BDCA4. Fluorochromes can be adapted, depending on the laser and filter settings of the FACS sorter.
2. Incubate the cell suspension for 30 min at 4°C
3. Wash the cell suspension with washing buffer
4. Resuspend the cell suspension at a concentration of 15×10^6^ cells/ml in washing buffer.
5. Sort pDC based on the expression of HLA-DR and BDCA4 and absence of Lin1-staning, cDC1 cells based on the expression of HLA-DR and CD141 and absence of Lin1-staining, and cDC2 cells based on the expression of HLA-DR and absence of Lin-1 staining.
6. Collect the sorted DC fractions in culture medium. For culturing of DC subsets, X VIVO 15 medium with 2% human serum is very suitable.

###### 2.2.3.3.4 Cell culturing.

Generally, the yield of DC subset isolation from PBMCs is low, due to the low frequencies. It is recommended to culture the cDC in flat bottom wells plates, the size of the well depending on the yield and number of cells to be cultured per condition, and pDC in round bottom well plates. All three DC subsets can be cultured in X VIVO 15 medium supplemented with 2% human serum. PDC and cDC2, but not cDC1, also reside well in TexMACS medium with 2% human serum. It is recommended to culture the cells at a concentration of 0.5 – 2×10^6^ cells per ml.

For proper DC maturation, yielding DC with a mature phenotype that produce proinflammatory cytokines and induce strong T cell activation, DC should be matured with synthetic or natural TLR ligands. Each DC subsets expresses a unique repertoire of TLRs and the outcome of DC maturation is dependent on the TLR ligand used [125, 126]. Overnight maturation with TLR ligands is sufficient to induce a fully mature phenotype and proinflammatory cytokine production by blood DC subsets. It is important to note that pDC that are not stimulated with a TLR ligand should be cultured in the presence of IL-3 to maintain a good viability.

###### 2.2.3.3.5 Sample preparation for purity analysis and phenotype analysis by fiow cytometry.

The purity of FACS sorted DC can be determined based on the cell labeling for sorting. Purity of MACS isolated DC subsets can be assessed for each DC subset separately, or for all three subsets simultaneously. We here give an example of purity analysis of all three DC subsets simultaneously in one sample using CD141 and Clec9A to stain cDC1, CD1c and FcɛRI to stain cDC2, and CD123 and BDCA2 to stain pDC.

To study if cultured and stimulated DC are phenotypically mature, the expression of MHC class I and class II, costimulatory molecules CD80 and CD86, DC maturation marker CD83, and lymph node homing receptor CCR7 can be assessed by flow cytometry. When the purity of the isolated cells is not very high, a DC marker could be added to the antibody-mix to enable gating for DC. When analyzing the phenotype of cDC1, which are very scarce and generally have an extremely low yield, a selection of phenotype markers could be analyzed, for instance CD80, CD83, and CD86.

1. Add at least 5.000 cells to a 96 wells V-bottom plate or FACS tubes.
2. Wash the cells with staining buffer
3. For purity analysis, add FcR blocking reagent and 25 μl of an antibody cocktail containing CD370 (Clec9a)-Viobright FITC, BDCA2-PE, CD14-PERCP, CD20-PERCP, CD1c-PE-Vio770, CD141 (BDCA3)-APC, CD123-APC-Vio770, FcɛRI-BioBlue, and CD45-VioGreen in staining buffer.
4. For phenotype analysis, add 25 μl of antibody diluted in staining buffer per sample. Antibodies used for phenotype analysis are HLA-ABC-APC (MHC class I), HLA-DR, DP, DQ-APC (MHC class II), CCR7-APC, CD80-APC, CD83-APC, and CD86-APC.
5. Mix well and incubate for 30 min at 4°C.
6. Wash twice with staining buffer.
7. Measure marker expression with a flow cytometer. Add Propidium Iodide just before measuring.

##### 2.2.3.4 Data analysis.

###### 2.2.3.4.1 Analysis of purity after MACS isolation.

**Figure 22.**
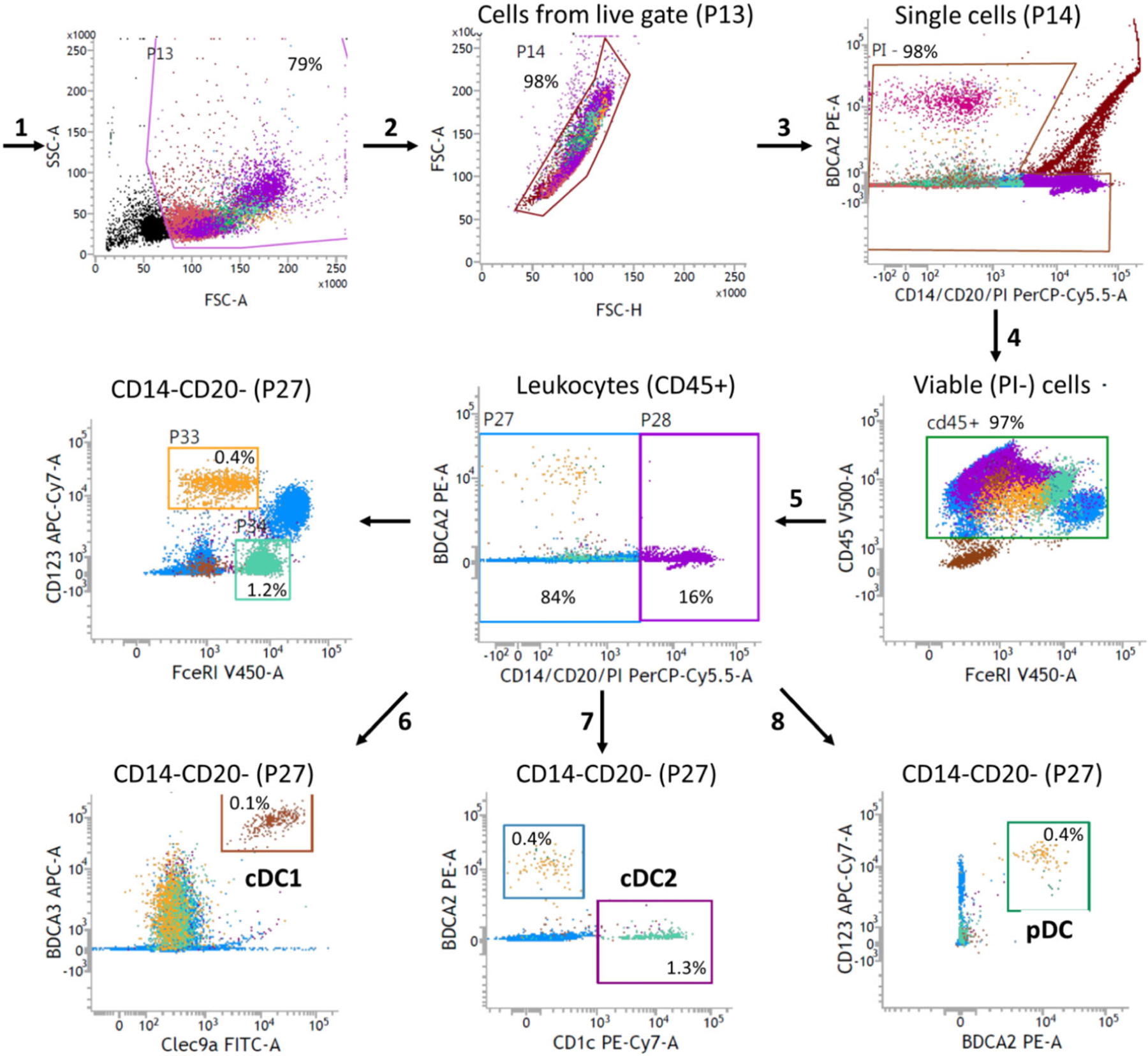
Gating strategy for purity analysis of naturally circulating DC subsets. In this example, the staining was performed on aphaeresis material before DC isolation.

The gating strategy for simultaneous analysis of the purity of pDC, cDC1, and cDC2 is shown in Fig. 22.

1. Gate the viable cell population in the FSC/SSC plot
2. Gate single cells and exclude doublets in the FSC-H/FSC-A plot.
3. Select viable cells by gating PI-negative cells.
4. Gate CD45-positive leukocytes.
5. Exclude B cells and monocytes by gating CD14/CD20 double negative cells in a PE/PerCP dotplot.
6. Select cDC1 cells by gating Clec9a/BDCA3 double positive cells.
7. Select cDC2 cells by gating CD1c positive cells within the CD14/CD20 negative population.
8. Select pDC by placing gating BDCA2/CD123 double positive cells.

###### 2.2.3.4.2 Analysis of phenotype after DC culturing.

**Figure 23.**
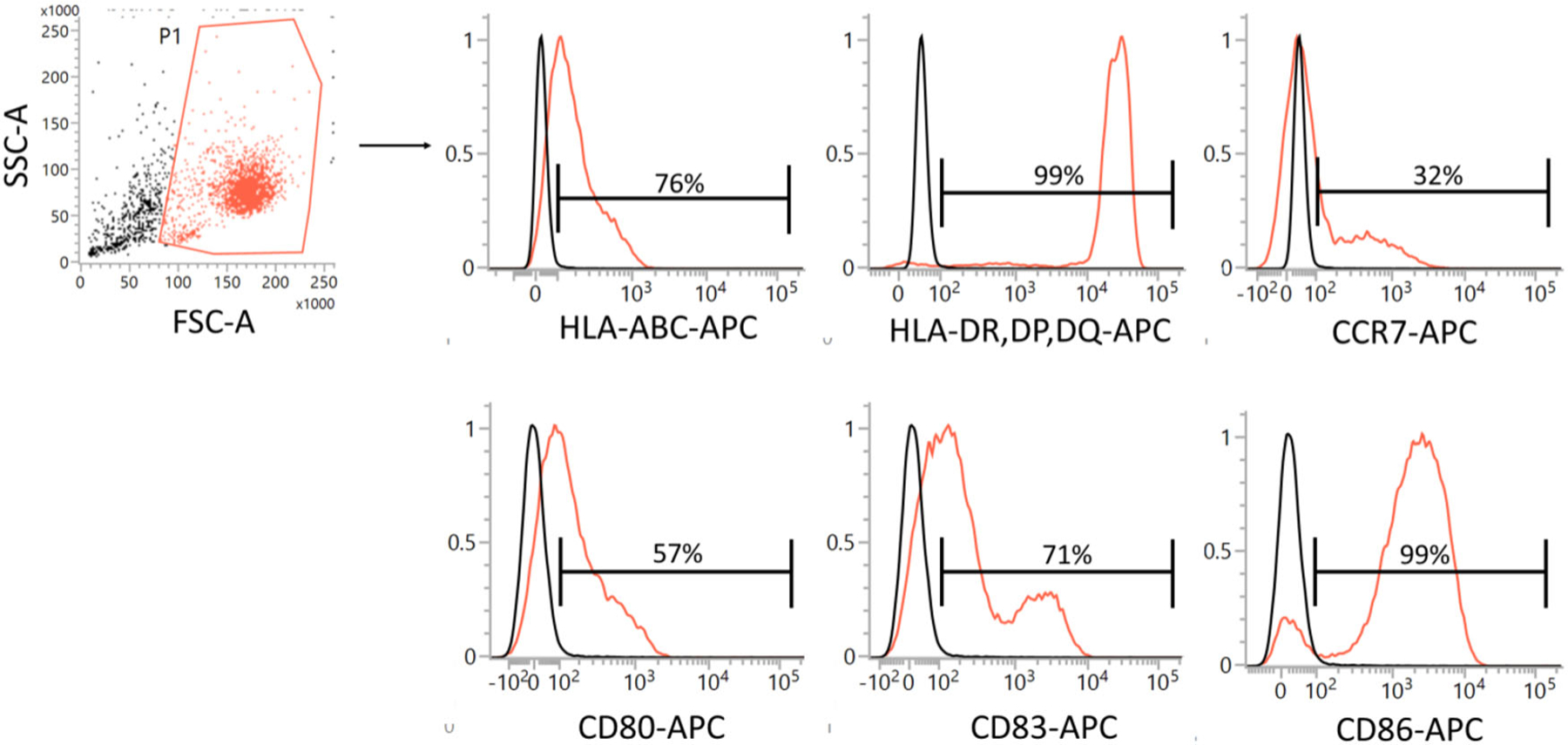
Gating strategy for phenotype analysis of cultured DC subsets. An example is given for cDC2. The same gating strategy can be followed for cDC1 or pDC.

An example of phenotype analysis of blood DC subsets is shown in Fig. 23.

1. Gate the viable cell population in the FSC/SSC plot.
2. Gate CD141-PE-positive cDC1, CD1c-Viobright FITC-positive cDC1, or BDCA2-positive pDC in a dot plot (optional; not shown in figure).
3. Determine the percentage of APC-positive cells or fluorescence intensity for each phenotype marker in histogram plots.

##### 2.2.3.5 Pitfalls.

In the past decades, a wealth of information has been generated by labs worldwide that study the behavior and functionality of DC subsets in health and disease. Although technical advances are continuously being developed, a major challenge remains the low frequencies of natural DC circulating in the blood. The average yields after isolation procedures from buffy coats are 0.1–0.5×10^6^ cDC1, 1–2×10^6^ cDC2, and 0.5–1.5×10^6^ pDC. However, these numbers vary greatly between donors and experiments as donor variability and buffy coat yield is very large. On occasion contrasting studies distinctly attribute cellular functions to isolated DC subsets, which fuels the discussion whether observed responses are possibly caused by contaminating cells. When lineage depletion is not complete, especially after MACS sorting impurities of CD3^+^ T cells CD20^+^ B cells and CD14^+^ monocytes can be found. However, in general purities of DC isolations are *>*90% for pDC and cDC2 and *>*70% for cDC1. Impurities in the isolated cell product could also include other DC subsets. This is relevant when studying functional characteristics of the DC subsets, as the subsets can cross-activate each other [127–129].

An advantage of FACS sorting over MACS-based isolation techniques is that all three subsets can be isolated simultaneously from the same donor with very high purity. However, to obtain a good yield, the sorting process takes a long time. Furthermore, and perhaps more importantly, FACS sorting can also be stressful for cells, thereby possibly impacting functional behavior. Isolation of alle three subsets from a single buffy coat or aphaeresis product by MACS sorting is also possible; then the subsets need to be sorted sequentially, which is also very time consuming (Florez-Grau et al, manuscript in preparation and [130]. Generally, pre-depletion of lineage-positive cells reduces the isolation time and increases the purity.

Another booming field to dissect the functional abilities of DC subsets and to probe their transcriptome is single cell RNA sequencing (scRNAseq). For these types of studies, it is imperative to ensure that the analyzed cell population does not contain any impurities. One approach to overcome this challenge is to make use of index sorting when cells are sorted single cell in single wells by FACS. In this way, the sorted events and the accompanying phenotype can be traced back after transcriptome analysis to warrant cell purity and to exclude the possibility that results are derived from contaminating cells. The number of protocols and approaches to perform scRNAseq is rapidly increasing and the current format precludes to deal with all the pros and cons of that field.

#### 2.2.4 Human DC for clinical use

DC for clinical use are classified as Advanced Therapy Medicinal Products (ATMPs). According to European legislation, their manufacturing must therefore be in compliance with good manufacturing practice (GMPr). The generation of complex cellular products under full GMPr-compliance is a challenging task. A large number of reagents and substances is used, which all need to be obtained in pharmaceutical quality and all equipment, procedures, and facilities need to be in compliance with the respective regulations as well. Proper documentation is another topic, filling complete books. Hence, we cannot provide any ready-to-use GMPr protocols here since any individual protocol, intended for use in clinical application needs to be discussed and agreed upon with the regulatory authorities, who’s advise should be sought early in the establishing process. Hence, the following paragraphs are rather intended as an advise for translational researchers, who intend to develop processes and protocols, which shall later be applied in clinical application and hence need to be transferred to GMPr. Find below some dos and don’ts, which should be adhered to from early on.

All reagents, that may end up in the final product need to have GMPr quality. The easiest source of such reagents, like e.g. cytokines are approved drugs. These are readily available, often cheaper than GMPr-reagents, and usually accepted by the authorities. An example for DC maturation is PGE_2_. In opposite, animal-derived substances like fetal bovine serum or BSA are to be avoided due to the issues with transmissible spongiform encephalopathies (TSE). In this context, it is important to arrange with all suppliers of kits and reagents that full content lists are available (if necessary after confidentiality agreements). Any substance that may be contaminating the final product (termed drug product) needs to be either analyzed for, or the maximum possible contamination needs to be evaluated by a risk assessment team and justified to the authorities. For example, when DC are pulsed with peptides, dissolved in DMSO, the final DMSO-contamination of your drug product will be way below any toxic level; nevertheless, it will convince the authorities more easily, if a proper calculation is provided. It is also very helpful if all providers offer lot-specific certificates of analysis for all goods that enter the production process, otherwise these analyses will have to be performed upon entry.

The second topic relevant for GMPr-production is quality control. All relevant properties of the final product need to be defined, although many values can be set arbitrarily as long as they can be justified and are accepted by the regulatory authorities. A number of surface markers to define mature DC are summarized in Table 14. The specifications in the table are indicative; they vary between DC subtypes and their expression can be dependent on the maturation stimulus used. In addition to phenotype analysis, potency testing is required for DC for clinical use. The potency assay should demonstrate the biological activity of the DC and should be based on the intended biological effect, i.e. T-cell activation. To warrant a stable production process, in-process controls are also strongly advised, i.e. purity of monocytes (when used for DC generation) or freshly isolated blood DC, phenotype of immature DC, etc. In this context clearly quantifiable values are preferable: A “percentage above 80 %” is good, a “typical DC-morphology in the microscope” is poor. However, keep in mind that all assays used need to be validated beforehand, unless described in the European Pharmacopoeia.

## Figures and Tables

**Table 1.** Reagents, antibodies, chemicals, and solutions

Reagent	Manufacturer	Ordering Number
Antibodies		
B220 APC-Cy7 (RA3-6B2)	Biolegend	103223
CD11b BV510 (M1/70)	Biolegend	101245
CD11c PE-Cy7 (N418)	Biolegend	117317
CD115 APC (AFS98)	eBioscience	17-1152-80
CD117 (c-Kit) PE-Cy7 (ACK2)	eBioscience	25-1172-82
CD135 (Flt3) PerCP-Cy5.5 (A2F10)	eBioscience	46-1351-82
Gr1 BV421 (RB6-8C5)	Biolegend	108433
Gr1 PerCP-Cy5.5 (RB6-8C5)	eBioscience	45-5931-80
MHCII PerCP-Cy5.5 (M5/114.15.2)	Biolegend	107625
XCR1 BV421 (ZET)	Biolegend	148216
Chemicals & Solutions		
β-estradiol (E2)	Sigma	E2758
β-mercaptoethanol (β-Me)	Gibco	31350010
Calcium chloride (CaCl_2_)	Merck	1.02382.0500
Chondroitin sulfate sodium salt from shark cartilage (CSC)	Sigma	C4384
Dimethysulfoxide (DMSO)	Sigma	D8418
DMEM	Gibco	41965039
EDTA	Gibco	15575-038
Fetal bovine serum (FBS)	PAA	A01125-499
Fetal bovine serum (FBS)	Gibco	10270106
Recombinant human Flt3-Ligand (Flt3L)	Peprotech	300-19
Recombinant murine SCF	Peprotech	250-03
Human IGF-1 long range	Sigma	85580C
Recombinant mouse IL-6/IL-6R alpha protein chimera	R&D Systems	9038-SR
HEPES	Sigma	H4034
L-glutamine	Gibco	25030081
Pancoll human, density 1.077g/ml (Ficoll)	PAN-Biotech	P04-601000
Penicillin/streptomycin	Gibco	15140122
Phosphate buffered saline (PBS)	Gibco	10010023
Polybrene (PB, Hexadimethrine bromide)	Sigma	H9268
RPMI 1640	Gibco	31870025
Sodium chloride (NaCl)	Merck	7647-15-5
Sodium phosphate dibasic (Na_2_HPO_4_)	Merck	7558-79-4

**Table 2.** Equipment

Equipment	Company	Purpose
Cell counter (CASY)	OMNI Life Science	Cell counting
Centrifuge (Sorvall X Pro Series)	Thermo Scientific	Centrifugation of 15 ml tubes and U-bottom plates
CO_2_ incubator (HERAcell 240)	Heraeus	Cell culture
Evos cell imaging systems (fl)	AMG	Cell imaging
FACS tube (#551579)	SARSTEDT	Regular FACS tubes for acquisition of single cell suspensions
Laminar flow hood (HERAsafe)	Thermo Scientific	Performance of all aseptic procedures
LSR Fortessa	BD	Flow cytometry analysis of single cell suspensions
Multi-channel pipette (20-200 μl)	VWR	Pipetting of cell suspension into 96-well plate for limiting dilution
Phase contrast microscope (DM IL)	Leica	Screening of single cell colonies from 96-well plate
Pipetboy	INTEGRA	Pipetting
Pipette tips	Greiner bio-one	Pipetting
Serological pipettes (#606180)	Greiner bio-one	Pipetting
96-well U-bottom plate (#650185)	Greiner bio-one	Limiting dilution for single cell colonies
48-well plate (#677180)	Greiner bio-one	Cell expansion from 96-well plate
24-well plate (#662160)	Greiner bio-one	Cell expansion from 48-well plate
12-well plate (#665180)	Greiner bio-one	Cell expansion from 24-well plate
6-well plate (#353046)	Corning	Cell expansion from 12-well plate
15 ml tubes (#188271)	Greiner bio-one	Centrifugation of cell suspensions
45 μm cellulose acetate membrane filter (#10462100)	Whatman	Sterile filtration
Vortex (Genie-2)	Scientific Industries	Sample mixing before flow cytometric analysis

**Table 3.** Reagents, enzymes, chemicals, and solutions^a)^

Reagent	Manufacturer	Ordering Number
Chemicals & Solutions		
RPMI 1640	Thermo Scientific	31870025
Dulbecco’s Modified Eagle Medium (DMEM), high glucose	Thermo Scientific	41965062
Dulbeccós Phosphate Buffered Saline (PBS), no calcium, no magnesium	Thermo Scientific	14190169
l-Glutamine (200 mM)	Thermo Scientific	25030-024
Penicillin-Streptomycin (10,000 U/mL)	Thermo Scientific	15140122
2-Mercaptoethanol (50 mM)	Thermo Scientific	31350010
Fetal bovine serum (FBS)	Gibco	10270106
UltraPure 0.5 M EDTA, pH8.0	Thermo Scientific	15575020
Bovine Serum Albumin (BSA)	Sigma	A8806
Trypan Blue solution	Sigma	T8154
Recombinant Human Flt3-Ligand	Peprotech	300-19
Propidium Iodide (PI) - 1.0 mg/mL Solution in Water	Thermo Scientific	P3566
Rainbow Calibration Particles	BD	556286
Antibodies		
CD11c-APC (clone N418)	BD	550261
CD11c-PE (clone N418)	eBioscience	12-0114-81
Siglec-H-PE (clone 551)	BD	129606
MHC-II-BV650 (clone M5/114.15.2)	Biolegend	107641
CCR9-PEcy7 (clone eBioCW1.2)	eBioscience	25-1991-82
XCR1-APCcy7 (clone REA707)	Milteny	130-111-375
Sirp-α-PerCPeF710 (clone p84)	eBioscience	46-1721-80
Sirp-α-APC (clone p84)	Biolegend	144013
CD24a-FITC (clone M1/69)	eBioscience	11-0242-81
CD11b-eF450 (clone M1/70)	eBioscience	48-0112-80
B220-PerCP-cy5.5 (clone RA3-6B2)	eBioscience	45-0452-80

a) Reagents can be purchased from other vendors. For antibodies, alternative clones and fluorochromes can be used but should tested in advance.

**Table 4.** Necessary equipment^a)^

Equipment	Company	Purpose
Centrifuge “Heraeus megafuge 16 series”	Thermo Scientific	Centrifugation of 50 ml tubes
CO_2_ incubator (HERAcell 240)	Thermo Scientific	Cell culture (5% CO_2_)
Laminar flow hood (HERAsafe)	Thermo Scientific	Performance of all aseptic procedures
Vortex (Genie-2)	Scientific Industries	Sample mixing
Counting chamber, Neubauer, improved, BLAUBRAND	Brand	Determination of cell concentration
Microscope “Primovert Series”, with Axiocam 208 color camera	Zeiss	Observation of cells and acquisition of images
PipetteBoy	Integra	Pipetting
Micropipettes	Eppendorf	Pipetting
Pipette tips	Greiner bio-one	Pipetting
Serological pipettes 5 ml (#606180)	Greiner bio-one	Pipetting
Serological pipettes 10 ml (#607180)	Greiner bio-one	Pipetting
Serological pipettes 25 ml (#760160)	Greiner bio-one	Pipetting
Falcon 15 mL Conical Centrifuge Tubes	Fisher Scientific	Centrifugation of cell suspensions
Falcon 50mL Conical Centrifuge Tubes	Fisher Scientific	Centrifugation of cell suspensions
70μm Cell Strainer for 50 ml tubes (#431751)	Corning	Filtration of cell suspension
Cellstar 6-well plate (#657160)	Greiner bio-one	Cell culture
Flow cytometry tubes (#551579)	Sarstedt	Flow cytometry
LSRFortessa Cell Analyzer (4 lasers)	BD	Flow cytometry

a) Equipment can be purchased from other vendors.

**Table 5.** 

Flt3L culture of mouse BM progenitors	cDC1	CD11c^+^ MHC-II^+^ XCR1^+^ CD24^hi^ CD11b^−/lo^ Sirp-α^−^
	cDC2	CD11c^+^ MHC-II^+^ Sirp-α^+^ CD11b^+^ CD24^lo^ XCR1^−^
	pDC	CD11c^low/+^ MHC-II^low^ B220^+^ CD11b^−/low^ Siglec-H^+^ CCR9^+^
	CDP	CD11c^−^ Siglec-H^−^ MHC-II^−^
	Pre-DC	CD11c^+^ Siglec-H^−^ MHC-II^−^

**Table 6.** iCD103-DC generation

Reagent or resource	Company	Catalog
RPMI 1640	Thermo Fisher Scientific	61870
Fetal calf serum (FCS) heat inactivated	Biochrom	S0115
2-Mercaptoethanol	Gibco	31350
Penicillin/streptomycin	Biochrom/Merck	A2213
DPBS	Sigma	D8537
Murine GM-CSF culture supernatant	Made in-house	N/A
Murine FLT3L-flag culture supernatant	Made in-house	N/A
Mouse GM-CSF duoset ELISA	R&D	DY415
Mouse FLT3L duoset ELISA	R&D	DY427
Petri dishes 10 cm, sterile	Sarstedt	821473001
50 ml tubes	Sarstedt	62547254
Cluster tubes (FACS) polypropylene 1.2 ml	Corning	4401
96-well cell culture plate, PS, F-bottom	Greiner Bio-one	655180
ODN 1826-TLR9 ligand (Class B CpG)	Invivogen	Tlrl-1826-5
Trypan Blue stain	Thermo Fisher Scientific	15250061

**Table 7.** Flow cytometry reagents

Reagent	Fluorochrome	Clone	Company	Catalog
Buffer				
Bovine serum albumin (BSA)	N/A	N/A	Carl Roth	80763
Paraformaldehyde (PFA)	N/A	N/A	Merck	158127
Ethylenediaminetetraacetic acid (EDTA)	N/A	N/A	Roth	8040
Sodium azide	N/A	N/A	Carl Roth	4221.1
Antibodies				
Live/dead fixable aqua dead stain	N/A	N/A	Invitrogen	L34957
Fc block (anti CD16/32)	N/A	N/A	Made in-house	N/A
CD11c	Alexa Fluor 488	N418	eBioscience	53-0114
CD11c	APC eFluor-710	N418	eBioscience	47-0114
Clec9A	PE	42D2	eBioscience	12-5975
Clec9A	PercP-eF710	42D2	eBioscience	46-5975
B220	PE-Cy7	RA3-6B2	eBioscience	25-0452
CD103	APC	2E7	eBioscience	17-1031
CD172a (SIRP-α)	Biotin	P84	eBioscience	13-1721
Streptavidin	eF450	N/A	eBioscience	48-4317
MHC-II	eF450	M5/114.15.2	eBioscience	48-5321
CD86	FITC	GL1	eBioscience	11-0862
CD80	FITC	16-10A1	eBioscience	11-0801
CD40	PE-Cy5	1C10	eBioscience	15-0401
CCR7	PE	4B12	eBioscience	12-1971

**Table 8.** List of necessary equipment

Equipment	Manufacturer	Model
Laminar flow hood	Thermo Fisher Scientific	Herasafe 2030i
Centrifuge	Thermo Fisher Scientific	Megafuge ST Plus Series
Inverted microscope	Zeiss	Primovert
Flow cytometer	Beckman Coulter	Cytoflex S V4-B2-Y4-R3 (13 detectors, 4 lasers)
Inverted microscope	Olympus	IX81

**Table 9.** Summary of subpopulations analyzed

Subpopulation	Phenotype
iCD103-DC	Live-Dead^−^ CD11c^+^ B220^−^ Clec9a^+^ CD103^(hi)^ SIRP-α^−^
Immature iCD103-DC	Live-Dead^−^ CD11c^+^ B220^−^ Clec9a^+^ CD103^(hi)^MHC-II^low^CD86^low^
Immature iCD103-DC	Live-Dead^−^ CD11c^+^ B220^−^ Clec9a^+^ CD103^(hi)^CD40^low^CD80^low^

**Table 10.** Staining for CD34^+^ purification by FACS

Antibody			
Fluorochrome	Antigen	Clone	Manufacturer
DAPI	live/dead		Sigma-Aldrich
BV510	CD3	OKT3	Biolegend
	CD19	HIB19	Biolegend
	CD20	2H7	Biolegend
	CD56	HCD56	Biolegend
APC	CD34	581	BD Biosciences

**Table 11.** Fluorochrome-conjugated antibodies used for phenotypic identification of differentiated DC subsets

Antibody			
Fluorochrome	Antigen	Clone	Manufacturer
FITC	CD3	SK7 (Leu-4)	BD Biosciences
	CD16	3G8	BD Biosciences
	CD19	4G7	BD Biosciences
	CD20	L27	BD Biosciences
	CD34	8G12	BD Biosciences
	CD56	NCAM16.2	BD Biosciences
APC	CD303/CD304	201A/12C2	BioLegend
APC-Cy7	CD45	2D1	BD Biosciences
AF700	CD11c	B-ly6	BD Biosciences
BV421	CD123	6H6	BioLegend
BV510	CD141	1A4	BioLegend
BV650	CD14	M5E2	BioLegend
BV711	CD163	GHI/61	BioLegend
BV785	HLA-DR	L243	BioLegend
PE	CLEC9A	8F9	BioLegend
PE-CF594	CD2	TS1/8	BioLegend
PE-Cy7	CD1c	L161	BioLegend
BUV395	CD15	HI98	BD Biosciences
DAPI	live/dead		Sigma

**Table 12.** Selected protocols for *in vitro* simultaneous generation of human cDC1, cDC2, pDC, and eventually DC3 from CD34^+^ HSC

References	Culture protocol	Generated cell types	Yields^a)^ thereof	Remarks and recommended use
Proietto et al. 2012 [92]	M: Yssel’s + 10% human AB serum C: huFLT3-L (100 ng/ml) + huTPO (50 ng/ml) No feeder cells Time: 21 d (medium exchange every 5 d)	**cDC1**: CD11c^int^HLA− DR^+^CLEC9A^+^CD14^−^CD172a^−/low^CD11b^−^*XCR1*^*−*b)^ **cDC2**: CD11c^+^HLA-DR^+^CD1c^+^CD14^−^CD172a^+^CD11b +**pDC**: CD11c^−^HLA-DR^+^CD123^+^CD14^−^CD172a^low/+^ CD11b^−^ **monocytes**: CD14^+^HLA-DR^+^	2×10^4^ 3×10^4^ 3×10^4^ 30×10^4^	• Differentiation analysis
Breton et al. 2015 [83]	M: α-MEM 1 with 10% FCS C: huFLT3-L (200 ng/ml), huGM-CSF, huSCF (40 ng/ml each) F: MS5 Time: 7 d	**cDC1:** **CD11c**^**+**^**HLA-DR**^**+**^**CD141**^**+**^**CLEC9A**^**+**^**CD1c**^**−**^**CD14**^**−**^**CD123**^**low**^ **cDC2: CD11c**^**+**^**HLA-DR**^**+**^**CD1c**^**+**^**CD141**^**−**^**CD14**^**−/low**^**CD123**^**low**^ **pDC: CD11c**^**−**^**HLA-DR**^**+**^**CD123**^**+**^**CD303**^**+**^**CD14**^**−**^**CD1c**^**−**^ **monocytes: CD45**^**+**^**CD14**^**+**^**CD16**^**−**^**CD66b**^**−**^	0.3×10^4^ 9×10^4^ 5×10^4^ 14×10^4^	• Differentiation analysis for DC types and several other hematopoietic lineages
Balan et al. 2018 [95]	M: a-MEM with 10% FCS C: expansion: huFLT3-L (25 ng/ml), huTPO (5 ng/ml), huSCF (2.5 ng/ml), hIL7 (5 ng/ml), hGM-CSF (1 ng/ml); differentiation: hFLT3L (15 ng/ml), hIL7 (5 ng/ml) hTPO (2.5 ng/ml), huGM-CSF (1 ng/ml) F: OP9 : OP9_DLL1 – 3:1 Time: 14–21 d (expansion 7 d + differentiation 7–14 d)	**cDC1**: CD11c^+^CD141^+^CLEC9A^+^CADM1^+^XCR1^+^CD1c^+^CD1a +**cDC2**: lin-HLA-DR^+^; *CLEC10A*^+^*CX3CR1*^+^*CD1A* +**pDC**: CD123^+^CD303^+^LILRA4^+^BTLA^+^CLEC9A^−^CADM1^−^XCR1^−^ **monocytes**: CD45^+^CD14^+^CD16^−^CD66b^−^	3×10^5^ 5×10^5^ 10×10^5^ 1×10^5^	• High frequency^c)^ and yields of pDC & cDC1 • in vitro-generated DC subsets match with their natural PB counterparts based on single cell RNA sequencing, phenotype and TLR responses • Differentiation analysis for the cDC1 and pDC lineages • Functional assays, including to evaluate impact of the genetic manipulation of amplified HSC
Kirkling et al. 2018 [96]	M: a-MEM with 10% FCS C: huFLT3-L (100 ng/ml), huGM-CSF, huSCF (20 ng/ml each) F: OP9_DL1 Time: 14–21 d (medium exchange every 7 d)	**cDC1**: lin-HLA-DR^+^CD141^+^CLEC9A^+^CD14^−^ **cDC2**: lin-HLA-DR^+^CD11c^+^CD1c^+^CD141^+/−^CLEC9A^−^CD14^−^ **pDC**: lin-HLA-DR^+^CD11c^−^CD123^+^CD14^−^ **monocytes**: lin-HLA-DR^+^CD14^+^	5×10^4^ 2×10^4^ 2×10^3^ 8×10^3^	• Predominantly cDC1 • Differentiation analysis
Cytlak et al. 2020 [101]	M: a-MEM with 10% FCS C: huFLT3-L (100 ng/ml), huGM-CSF, huSCF (20 ng/ml each) F: OP9 Time: 14–21 d (medium exchange every 7 d)	**cDC1**: CD45^+^CD15^−^CD34^−^HLA-DR^+^CD141^+^CLEC9A +**cDC2**: CD45^+^CD15^−^CD34^−^HLA- DR^+^CD123^−^CD1c^+^CD2^+^CD14^−^CD5^+/−^ **DC3**: CD45^+^CD15^−^CD34^−^HLA-DR^+^CD123^−^CD1c^+^CD2^+^CD14^+^CD5^−^ **pDC**: CD45^+^CD15^−^CD34^−^HLA-DR^+^CD123^+^CD303/4 +monocytes: CD45^+^CD15^−^CD34^−^HLA-DR^+^CD123^−^CD1c^−^CD2^−^CD14^+^	1×10^3^ 4×10^4^ 2×10^4^ 1×10^3^ 2×10^3^	• Predominantly cDC2 & DC3 • Differentiation analysis of the cDC2 versus DC3 lineages
Thordardottir et al. 2014 [97]	M: Glycostem Basal Growth Medium C: huFLT3-L, huTPO, huSCF, IL-6 (100 ng/ml each) + 1μM StemRegenin 1 (SR1) Time: 21 d (medium exchange every 2–4 d)	**cDC1**: HLA-DR^+^CD141^+^CD1c^−^CD14^−^CD123^low^ **cDC2**: HLA-DR^+^CD1c^+^CD141^−^CD14^−^CD123^low^ **pDC**: CD11c^−^HLA-DR^+^CD123^+^CD303^+^ CD14^−^	1.2×10^5 d)^ 5.3×10^5^ 3.8×10^5^	• Functional assays • Clinical application
**I** Thordardottir et al. 2017 [98]	M: Cellgro DC medium (GMPr compliant) with 2% human serum, 50 μg/ml ascorbic acid, 1 μM SR1 C: huFLT3-L (100 ng/ml), huTPO, huSCF (100 ng/ml each for 7 d then 50 ng/ml) Time: 14–20 d	**cDC1**: HLA-DR^+^CD141^+^CLEC9A^+^CD1c^−^CD14^−^CD123^low^ **cDC2**: HLA-DR^+^CD1c^+^CD141^−^CD14^−^CD123^low^ **pDC**: CD11c^−^CD123^+^CD303^+^ CD14^−^	0.1×10^5^ 1.8×10^5^ 1.6×10^5^	• High frequency of pDC and cDC2 • GMPr-compliant clinical application
**II** Thordardottir et al. 2017 [98]	M: Cellgro DC medium (GMPr compliant) with 2% human serum, 50 μg/ml ascorbic acid, 1 μM SR1 C: expansion: huFLT3-L, huTPO, huSCF (100 ng/ml each); differentiation: huGM-CSF (800 IU/ml), huIL-4 (500 IU/ml) Time: 14–21 d (expansion 7–13 d + differentiation 7 d)	**cDC1**: HLA-DR^+^CD141^+^CD1c^−^CD14^−^CD123^low^ **cDC2**: HLA-DR^+^CD1c^+^CD141^−^CD14^−^CD123^low^ **pDC**: CD11c^−^CD123^+^CD303^+^ CD14^−^	0.1×10^5^ 1.8×10^5^ 0.2×10^5^	• High frequency of cDC2 • Increased expression of CD11c & HLA-DR on cDC1 &cDC2 • GMPr-compliant clinical application
van Eck van der Sluijs et al. 2021 [99]	M: Cellgro DC medium (GMPr compliant) with 2% human serum, 50 μg/ml ascorbic acid, 1μM SR1 C: expansion: huFLT3-L, huTPO, huSCF (100 ng/ml each); differentiation: huGM-CSF (800 IU/ml), huFLT3-L (100 ng/ml), huIFN-a (1000 IU/ml) Time: 14 d (expansion 7 d + differentiation 7 d)	**cDC1**: HLA-DR^+^CD141^+^CD1c^−^CD14^−^CD123^low^ **cDC2**: HLA-DR^+^CD1c^+^CD141^−^CD14^−^CD123^low^ **pDC**: CD11c^−^CD123^+^CD303^+^ CD14^−^	0.5×10^5^ 3×10^5^ 1.6×10^5^	• High frequency of cDC2 • Match in vitro-generated DC subsets with their natural counterparts from peripheral blood, based on transcriptome, phenotype and function • G-CSF mobilized CD34^+^ HPCs • GMPr-compliant clinical application

M: medium; C: cytokines; F: feeder cells.

a) Yield refers to 10^4^ CD34^+^ input HSC.

b) Italic letters refer to marker gene expression;

c) “high frequency” refers to more than 10% of all the cells present in the culture;

d) Overestimation since a significant fraction of the CD141^+^ DC are shown to be negative for CLEC9A

**Table 13.** Proposed guidelines to ensure of the identity of the DC types generated in vitro, as compared to cells of the monocyte/macrophage lineages

Cell type	Phenotypic identity^a)^	Positive transcriptomic signature^b)^	Negative transcriptomic signature^c)^	Key functions that can be tested *in vitro*	Additional characteristics	References
cDC1	**HLA-DR^+^ CD11c^+^** CD33^+^ CD141^+^ **CLEC9A^+^** XCR1^+^ CADM1^+^ BTLA^hi^	***C1orf54***, ***CADM1***, ***CLEC9A***, ***CLNK***, ***CPNE3***, ***GCSAM***, ***NAAA***, ***WDFY4***, RAB32, ***XCR1***, *BATF3*, *BTLA*, *HLA-DOB*, *ID2*, *IDO1*, *IRF8*	NN	• IFN-III production upon TLR3 stimulation. • IL-12p70 production upon TLR8 stimulation. • High efficacy for cross-presentation of (dead-) cell-associated Ag.	• BATF3- and IRF8-dependent differentiation.	[78, 89, 95, 96, 106–110]
cDC2	**HLA-DR^+^ CD11c^+^** CD33^+^ **CD1c^+^ CLEC10A^+^** FCER1A^+^ CD5lo_to_hi BTLAlo_to_hi CD14– **CD163^−^** CX3CR1^+^ **CD88^−^**	***CD1C***, ***CD1E***, ***CLEC10A***, ***FCER1A***, *BTLA*, *CD5*, *FLT3*, *HLA-DOB*, *IRF4*	***C3AR1***, ***C5AR1***, ***CD14***, ***CD163, S100A8***, ***S100A9***, ***FCN1***, ***VCAN***	• IL-12p70 production upon triggering of CD40 and TLR8, IL-23 production. • Functional polarization of CD4^+^ T cells toward Th17 or Th2?	• IRF8-dependent differentiation.	[96, 101, 105, 107–111]
DC3	**HLA-DR^+^ CD11c^+^** CD33^+^ **CD1c^+^ CD163^+^** CD14lo_to_hi **CD5–** BTLA^−^ **CD88^−^**	***CD14***, ***CD163***, ***CD1C***, ***CLEC10A***, ***FCER1A***, ***S100A8***, ***S100A9***, *CDKN1A*, *CLEC4E*, *F13A1*, *FCN1*, *LMNA*, *VCAN*	***C1QA***, ***C1QB***, ***C1QC***, ***C3AR1***, ***C5AR1***, ***MERTK***	• TGFβ and IL-1β production. • T_RM_ differentiation.	• Differentiation does not require as high IRF8 expression as for cDC2.	[101, 111, 112]
ASDC	**HLA-DR^+^ CD11c^low/+^ CD33^+^** CD123^low/+^ **CLEC4C^+^ SIGLEC6^+^ AXL^+^** CX3CR1^+^ CD2^+^ CD5^+^	***AXL***, ***CD123***, ***SIGLEC1***, ***SIGLEC6***, ***TCF4***, *BCL11A*, *KLF12*, *LYZ*	***GZMB***, ***LILRA4***, ***NLRP7***, ***PACSIN1***	• Lack of IFN-I/III production upon TLR7 or TLR9 stimulation • High antigen presentation capacity, similar to that of cDC	• Dendritic morphology • TCF4-dependent differentiation. • High susceptibility to HIV-1 infection	[107–109, 113]
pDC	**HLA-DR^+^ CD11c^−^ CD33^−^** CD123^+^ **CLEC4C^+^ CD2^−^** CD5^−^	***CD123*, *CLEC4C***, ***GZMB***, ***IRF7***, ***LILRA4***, ***NLRP7***, ***PACSIN1***, ***TCF4***, ***TLR7***, ***TLR9***, *BCL11A*, *EPHB1*, *PTPRS*, *RUNX2*, *SPIB*	***ZBTB46***	• IFN-I/III production upon TLR7 or TLR9 stimulation. • Lower steady state antigen presentation capacity than cDC, but that is strongly boosted upon adequate stimulation.	• Plasmacytoid morphology. • TCF4-dependent differentiation.	[95, 105, 107–109, 114, 115]
MoDC	**HLA-DR^+^ CD11c^hi^** CD11b^hi^ **CD172a^hi^ CD163^−^** CD1a^+^ **CD206low/+ CD209low/+**	***CD1A***, ***CD1C***, ***CD209***, ***FCER1A***, ***FCER2***, ***FCGR2A***, ***FCGR2B***, ***FCGR2C***, ***FCGR3A***, ***IRF4***, ***ITGAM***, ***LILRB2***, ***MRC1***, ***S100A8***, ***S100A9***, ***SIRPA***, ***STAB1***, ***TLR2***, ***TLR4***, ***VCAN***, *CD14*, *CD226*, *CD68*, *CLEC10A*, *CSF1R*, *EBI3*, *LAMP3*, *MMP12*	***C1QA***, ***C1QB***, ***C1QC***, ***CD163***, ***FLT3***, ***MERTK***	• Production of high levels of TNF, CXCL1, CCL7 and IL-10 in response to TLR4 triggering. • IL-23 production upon CD40 triggering. • Functional polarization of CD4^+^ T cells toward Th17 or Tfh (or Th2?).	• Dendritic morphology • IRF4-dependent differentiation.	[30, 85, 89, 105, 116, 117]
LC	**HLA-DR^+^ CD11c^hi^** CD11b^+^ **CD1a^+^ CD207^+^** E-Cad^+^	***CD207***, ***EPCAM***, *ABCC4*, *AXIN2*, *BMPR1A*, *MICAL2*, *MYO6*, *TJP1*	NN	• High efficacy for CD8^+^ T cell priming.	• Birbeck granules.	[87, 106]
CD14^+^ DDC	**HLA-DR^+^ CD11c^+^** CD11b^hi^ **CD1a^−^ CD14^hi^** CD207^−^ E-Cad^−^	***CD14***, ***CEBPB***, ***MAFB***, ***MSR1***, ***TLR4***		• Functional polarization of CD4^+^ T cells toward Tfh.	• Dendritic morphology.	[87, 106]
cMo	**CD88^+^** CD11b^hi^ **CD172a^hi^ CD14^+^ CD163^−^ CD206^−^**	***CD14***, ***CEBPB***, ***FCN1***, ***LYZ***, ***S100A8***, ***S100A9***, ***VCAN***	***C1QA***, ***C1QB***, ***C1QC***, ***CD163***, ***FLT3***, ***MERTK***, ***ZBTB46***	• Direct B cell stimulation. • Low phagocytosis compared to macrophages • Low antigen presentation ability compared to cDC	• Non-dendritic and non foamy morphology	[105, 110]
MoMac	**CD88^+^** CD11b^hi^ **CD172a^hi^ CD16^+^ CD163^+^ CD206^+^** CD1a^−^	***APOE***, ***C1QA***, ***C1QB***, ***C1QC***, ***C3AR1***, ***C5AR1***, *CD163*, *FCGR3A*, *FOLR2*, *MERTK*	NN	• High phagocytosis. • Production of high levels of TNF in response to TLR4 triggering.	• Foam cell morphology. • MAFB-dependent differentiation.	[30, 85, 105, 110]

a) The minimal set of phenotypic markers recommended to identify the cell types is highlighted in bold. – −

b) The minimal set of positive marker genes recommended to identify the cell types is highlighted in bold.

c) The minimal set of negative marker gene recommended to discriminate the target cell type from the other types of mononuclear phagocytes that harbor the closest gene expression profiles is highlighted in bold. NN, not necessary, the use of negative marker genes is not necessary.

**Table 14.** Suggested parameters for quality control of DC for clinical use

Parameter	Specification
*Content*	
Cell count	Dependent on dose
Viability	*>*80%
*Purity*	
CD11c + HLA-DR on all cells	*>*90%
*Impurities*	
Small cells	*<*10%
CD14 on all cells	*<*20%
*Identity (phenotype)*	
CD80 on large cells	*>*70%
CD83 on large cells	*>*70%
CD83 on all cells	*>*60%
CD86 on large cells	*>*90%
*Potency*	
T cell activation	Dependent on assay
*Contaminations*	
Sterility	Sterile
Endotoxin	Dependent on volume and dose
Mycoplasma	Negative

## Data Availability

The data that support the findings of this study are available from the lead author(s) upon reasonable request.

## Abbreviations:

ANR: Agence Nationale de la Recherche
ATMPs: Advanced Therapy Medicinal Products
BDCA: Blood Dendritic Cell Antigen
BM MNC: BM mononuclear cells
cDC: conventional DC
CDP: common dendritic cell progenitor
cMoP: common monocyte progenitors
CSC: Chondroitin sulfate C
CSC: Chondroitin sulfate sodium salt from shark cartilage
CTV: Cell Trace Violet
DPBS: Dulbecco’s Phosphate Buffered Saline
FACS: Fluorescence Activated Cell Sorting
FC: flow cytometry
FLT3L: fms-like tyrosine kinase 3 ligand
FSC-A and SSC-A: forward and side scatter area
GMPr: good manufacturing practice
GMP: granulocyte macrophage progenitors
HoxB8 MPP: HoxB8 multipotent progenitors
HoxB8-ER: HoxB8 estrogen receptor fusion gene
HSC: hematopoietic stem cells
HSPC: hematopoietic stem/progenitor cells
LC: Langerhans cells
MDP: monocyte DC progenitors
MDSC: monocytic myeloid-derived suppressor cells
moDC: monocyte-derived DC
MPP: Multipotent progenitors
MSC: mesenchymal stem cells
NAF: non-adherent fraction
OP9-DL1: OP9 stromal cells expressing Delta-like 1
PB: Polybrene
PB/CSC: polybrene/chondroitin sulfate C
pDC: plasmacytoid DC
PFA: Paraformaldehyde
SCF: Stem Cell Factor
scRNAseq: single cell RNA sequencing
SIRP-α: signal regulatory protein α
SSC-A and SSC-H: area vs the height of SSC
TLR: Toll-like receptor
TSEs: transmissible spongiform encephalopathies
WEHI: Walter and Eliza Hall Institute
β-Me: β-mercaptoethanol
